# Unveiling the wound-healing potential of *Pisum sativum* aerial biomass through integrated bioassay-guided, LC–MS/MS, and network pharmacology approaches

**DOI:** 10.1186/s12906-026-05311-8

**Published:** 2026-03-23

**Authors:** Noha M. Kadash, Abdullah A. Elgazar, Mona El-Aasr, Ramadan A. El-Domany, Marwa Balaha, Fardous F. El-Senduny, Viviana di Giacomo, Mai H. ElNaggar

**Affiliations:** 1 Department of Pharmacognosy, Faculty of Pharmacy, Kafrelsheikh University, Kafrelsheikh, P.O. Box 33516, Egypt; 2 Department of Pharmacognosy, Faculty of Pharmacy, Tanta University, El- Geish Street, Tanta, 31111 Egypt; 3 Department of Microbiology and Immunology, Faculty of Pharmacy, Kafrelsheikh University, 33516, Kafrelsheikh, Egypt; 4 Department of Pharmaceutical Chemistry, Faculty of Pharmacy, Kafrelsheikh University, P.O. Box 33516, Kafrelsheikh, Egypt; 5 Department of Pharmacy, “G. d’Annunzio” University of Chieti-Pescara, P.O. Box 66100, Chieti, Italy; 6 Biochemistry division, Chemistry Department, Faculty of Science, Mansoura University, Mansoura, 35516 Egypt; 7 Department of Pathology and Laboratory Medicine, Sylvester Comprehensive Cancer Center, University of Miami, Miami, FL 33136 USA; 8 UdA Tech Lab, G. d’Annunzio University of Chieti-Pescara, P.O. Box 66100, Chieti, Italy

**Keywords:** *Pisum sativum* L., Anti-inflammatory properties, Wound healing, IL-6 suppression, LC–MS/MS analysis, PI3K pathway

## Abstract

**Background:**

While *Pisum sativum* L. is primarily valued for its seeds, its aerial parts have received limited attention. This study aimed to explore the anti-inflammatory and wound-healing potential of pea leaves and stems to promote their broader utilization.

**Methods:**

A bioassay-guided strategy was applied to various fractions of *P. sativum* aerial parts to evaluate their immunomodulatory activity using leukocyte accumulation assay. Active fractions were further assessed in lipopolysaccharide (LPS)-stimulated RAW 264.7 macrophages for their effects on TNF-α gene expression, interleukin-6 (IL-6), and nitric oxide production. The bioactive petrol eum ether (PSP) and ethyl acetate (PSE) fractions were further analyzed through GC-MS analysis, LC-MS/MS analysis, and chromatographic purification. The purified compounds were evaluated in human monocytes for their effects on IL-6 secretion and intracellular reactive oxygen species (ROS) production, and in HaCaT keratinocytes for their wound-healing potential using a scratch assay. Network pharmacology and molecular docking analyses were employed as in-silico tools to predict key targets and mechanistic pathways.

**Results:**

Both PSE and PSP fractions promoted leukocyte accumulation and significantly decreased TNF-α expression, IL-6, and nitric oxide production levels. LC-MS/MS analysis of PSE revealed 42 compounds, predominantly flavonoids and phenolics, potentially accounting for its anti-inflammatory activity. Among seven isolated constituents, pisatin exhibited superior activity by markedly suppressing IL-6 and ROS in human monocytes and promoting 73% wound closure in keratinocytes within 48 h.

**Conclusions:**

These findings establish *P. sativum* aerial parts as an accessible and valuable source of natural products with potential wound-healing activity, supporting the sustainable use of these underexploited agricultural materials.

**Supplementary Information:**

The online version contains supplementary material available at 10.1186/s12906-026-05311-8.

## Introduction

*Pisum sativum* L. (family Fabaceae), mostly known as green pea, or dry pea, is a substantial legume crop valued for its high protein content, essential vitamins, minerals, and bioactive constituents that contribute to human health [1]. Peas are grown in nearly every country worldwide and are considered a vital component of the human diet [2]. In addition to its nutritional benefits, consuming pea seeds is linked to various health-promoting properties, such as antifungal, anti-obesity, anti-cancer, and cardio-protective effects [3]. The therapeutic benefits of legumes is due to their rich content of secondary metabolites, particularly phenolics like flavonoids, isoflavonoids, catechins, anthocyanins, tannins, and coumarins [4].

While most studies have focused on the seeds and sprouts of *P. sativum* [5], other aerial parts such as leaves, stems, and pods also possess notable nutritional and medicinal potential [6]. These parts represent a valuable source of bioactive compounds and are especially promising due to their large biomass [7]. However, the majority of studies have concentrated on pea seeds, whereas other aerial parts have been comparatively neglected in terms of biological investigation, resulting in underutilization of the plant’s full potential.

Wound healing is a multifaceted biological process consisting of several interrelated phases: hemostasis, inflammation, proliferation, and remodeling [8]. Inflammation is a biological process in response to infection, injury or irritation [9]. It is a common feature of many diseases [10], and plays an essential role in healing wounds. It helps remove damaged cells and extracellular matrix (ECM), eliminate pathogens, and secrete mediators that regulate key processes such as cell proliferation, re-epithelialization, and tissue remodeling [11]. However, excessive or prolonged inflammation can impede healing, causing fibrosis and chronic wounds with a persistent inflammatory microenvironment, high pro-inflammatory macrophages, inflammatory mediators such as TNF-α, and increased activity of matrix metalloproteinases (MMPs) and reactive oxygen species (ROS) [12]. Accordingly, the identification of bioactive agents capable of modulating inflammatory signaling pathways is essential for improving wound healing outcomes.

Although the anti-inflammatory properties of different parts of *P. sativum* L. have been previously reported [13, 14], their potential role in wound healing, particularly for the aerial parts, remains insufficiently explored. Therefore, the present study aimed to investigate the anti-inflammatory and wound-healing potential of *P. sativum* L. aerial parts. Furthermore, phytochemical bio-guided isolation and in-silico studies were employed to identify the bioactive constituents and elucidate their underlying mechanisms of action.

## Materials and methods

### Plant material, extraction and preparation of different fractions

The dried aerial parts of *Pisum sativum* L. were collected from cultivated private agricultural fields in Seberbay area, Tanta, Gharbia Governorate, Egypt (30.81368° N, 31.01526° E), in November 2020, after obtaining prior permission from the field operator. The plant was identified by Prof. Yassin M. Al-Sodany, Prof. of plant Ecology and Flora, Department of Botany and Microbiology, Faculty of Science, Kafr Elsheikh University, Egypt. A voucher sample was placed in the department of pharmacognosy, Kafr Elsheikh University under ID number 2020-KFS-1. The aerial parts of *P. sativum* L. were extracted with methanol to obtain the total methanolic extract (PST), which was subsequently fractionated using different organic solvents, yielding four fractions: petroleum ether (PSP), methylene chloride (PSM), ethyl acetate (PSE), and *n*-butanol (PSB), as detailed in the supplementary material.

### Determination of total flavonoid and total phenolic contents in different fractions of *P. sativum* L.

Total flavonoid content was assessed using the aluminum chloride colorimetric assay [15]. While, the total phenolic content was determined using the Folin-Ciocalteu method [16]. Additional details are provided in the supplementary material.

### Evaluation of the biological activity of the obtained fractions

The immunomodulatory activity of PST, PSP, SPM, PSE, and PSB were initially evaluated on human peripheral blood mononuclear cells (PBMCs) using water-soluble tetrazolium salt-1 (WST-1) assay [17]. Fractions achieving more than 80% relative leukocyte accumulation were considered biologically active. Based on this criterion, PSP and PSE were identified as the most active and were selected for further investigation of their cytokine-modulating effects. For this purpose, RAW 264.7 murine macrophages were pretreated with PSP or PSE fractions (25 µg/mL), or dexamethasone (1 µM), dissolved in DMSO (final concentration ≤ 0.01% v/v) for 3 h, then stimulated with lipopolysaccharide (LPS, 5 µg/mL) for 24 h. Total RNA was then extracted, and TNF-α gene expression was analyzed by quantitative real-time PCR (qPCR) [18]. Supernatants were collected for further cytokine and nitric oxide (NO) analysis, where IL-6 levels were measured via ELISA [19], and NO levels were quantified using the Griess reagent assay [20] as detailed in the supplementary material.

### Chemical investigation and isolation of major compounds from the bioactive fractions of *Pisum sativum* L.

**Scheme 1:**
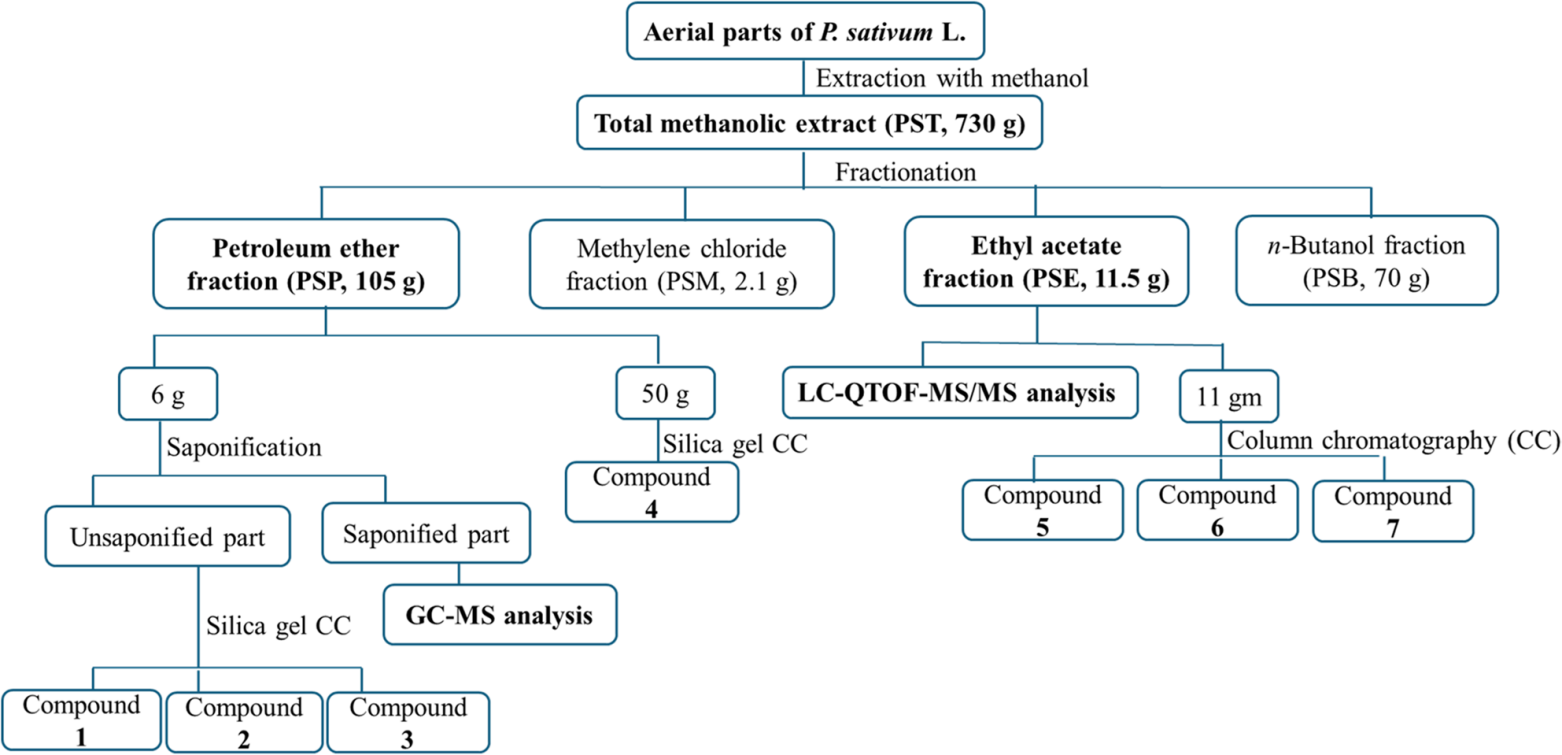
Bio-guided fractionation and isolation of pure compounds from the arial parts of *P. sativum* L

The petroleum ether (PSP) and ethyl acetate (PSE) fractions of the aerial parts of *P. sativum* L., showing the highest anti-inflammatory and immunomodulatory activities, were further subjected to chromatographic isolation and chemical analysis, as detailed in the supplementary material. Briefly, the saponifiable matter in the petroleum ether fraction (PSP) was analyzed using GC-MS, while LC-MS/MS was employed for the metabolic profiling of the ethyl acetate fraction (PSE). Major compounds from both fractions were purified using column chromatography to give compounds (1–7, Scheme 1).

### Evaluation of the immunomodulatory activity of pure compounds 3, 4, 6, and 7 isolated from PSP and PSE fractions of *P. sativum* L.

#### Impact on cell viability, IL-6 Secretion, and Reactive Oxygen Species (ROS) generation in human monocytes

Human monocytes (CRL-9855™, obtained from the American Type Culture Collection, ATCC) were seeded at (1.5 × 10^4^) cells/well in 96-well plates. Cells were then pretreated with LPS (50 ng/mL) for one hour, followed by treatment with test compounds (3, 4, 6, and 7) at 1, 5, and 10 µM, all dissolved in DMSO (final concentration ≤ 0.01% v/v). Dexamethasone (40 ng/mL) served as a positive control. After 24 h, cell metabolic activity was evaluated using the MTS assay. MTS reagent (0.5 mg/mL) was introduced to each well, and plates were incubated for one hour at 37 °C. Absorbance was measured at 490 nm with a Multiscan GO spectrophotometer (Thermo Fisher Scientific). Cell viability was calculated relative to untreated controls.

To evaluate the impact of the tested compounds on IL-6 secretion, their non-toxic doses, as determined from the previous cytotoxicity assay, were applied to LPS-stimulated monocytes. After 24 h, supernatants were collected, and IL-6 levels were quantified using an ELISA kit (Enzo Life Sciences Inc., Lausen, Switzerland). Results were normalized to MTS assay data to account for variations in cell viability. The intracellular ROS production in monocytes following treatment with the tested compounds was assessed using flow cytometry. Monocytes were pre-treated with LPS (50 ng/mL) for one hour, followed by treatment with the tested compounds at 5 and 10 µM for 24 h. Cells were labeled with CM-H2DCFDA (5 µM, Molecular Probes, Invitrogen) and incubated for one hour at 37 °C. ROS production was quantified using a Cytoflex flow cytometer, where an increase in green fluorescence intensity corresponded to higher ROS levels (Fig. S78). Data were expressed as the median fluorescence intensity (MFI) ratio relative to controls.

#### Impact on cell viability and wound healing in HaCaT Keratinocytes

HaCaT keratinocytes (obtained from CLS Germany) were seeded at (6 × 10^3^) cells per well in 96-well plates and permitted to adhere for 24 h. Cells were then treated with the tested compounds, dissolved in DMSO (final concentration ≤ 0.01% v/v), at concentrations ranging from 0 to 80 µM for 24 and 48 h. Cytotoxicity was evaluated using crystal violet assay. After treatment, cells were washed with phosphate-buffered saline (PBS), fixed with 1% glutaraldehyde, and stained with 0.02% crystal violet solution for 30 min at room temperature. Stained cells were carefully rinsed, and the crystal violet dye was solubilized with 70% ethanol. Absorbance was measured at 600 nm using a Multiskan GO spectrophotometer (Thermo Fisher Scientific), and cell viability was calculated relative to untreated controls.

To assess the wound-healing effects of compounds 3, 4, 6, and 7, a scratch assay was performed using HaCaT keratinocytes. Cells were seeded in 6-well plates at a density of (1.5 × 10^5^) cells per well. Upon reaching confluence, a scratch was made in the cell monolayer, and cells were treated with the tested compounds at 10 µM concentration. Images were captured at 0, 24, and 48 h using a Leica Dmi1 inverted microscope. The wound area was analyzed using Image J software, and results were presented as the percentage reduction in wound area relative to the initial scratch width.

### In-silico prediction of pisatin molecular targets and molecular docking analysis

Given the remarkable wound healing activity of pisatin, in-silico tools were employed to gain insights into its potential mechanism of action. This involved predicting the molecular targets of pisatin and correlating them with known molecular targets associated with wound healing. Additionally, a molecular docking study was conducted to explore pisatin’s interactions with the most significantly identified target, PI3K, as explained in the supplementary material.

### Statistical analysis

Statistical analyses were performed using GraphPad Prism 8.0.1 (GraphPad Software, Inc., San Diego, CA, USA). Data are presented as mean ± standard error of the mean (SEM) or standard deviation (SD), depending on the experiment. “n” refers to the number of biological replicates; each performed with technical replicates as indicated in the figure legends. Statistical significance was determined using one-way ANOVA followed by Tukey’s or Dunnett’s multiple comparisons test, as appropriate. A p-value of < 0.05 was considered statistically significant.

## Results and discussion

### Plant material, extraction and preparation of different fractions

Methanol extraction of 5 kg of the dried aerial parts of *P. sativum* L. yielded 730 g of total methanolic extract (PST), corresponding to an extractive yield of 14.6% (w/w). Subsequent fractionation with solvents of increasing polarity produced four fractions: 100 g of petroleum ether fraction (PSP), 2.1 g of methylene chloride fraction (PSM), 11.5 g of ethyl acetate fraction (PSE), and 70 g of n-butanol fraction (PSB). Saponification of a portion of the PSP fraction afforded 1.3 g of unsaponifiable matter (21.6%) and 3.7 g of saponifiable free fatty acids accounting for 61.66% of the total lipoidal content.

### Determination of the total flavonoid and total phenolic contents in different fractions of *P. sativum*

**Fig. 1:**
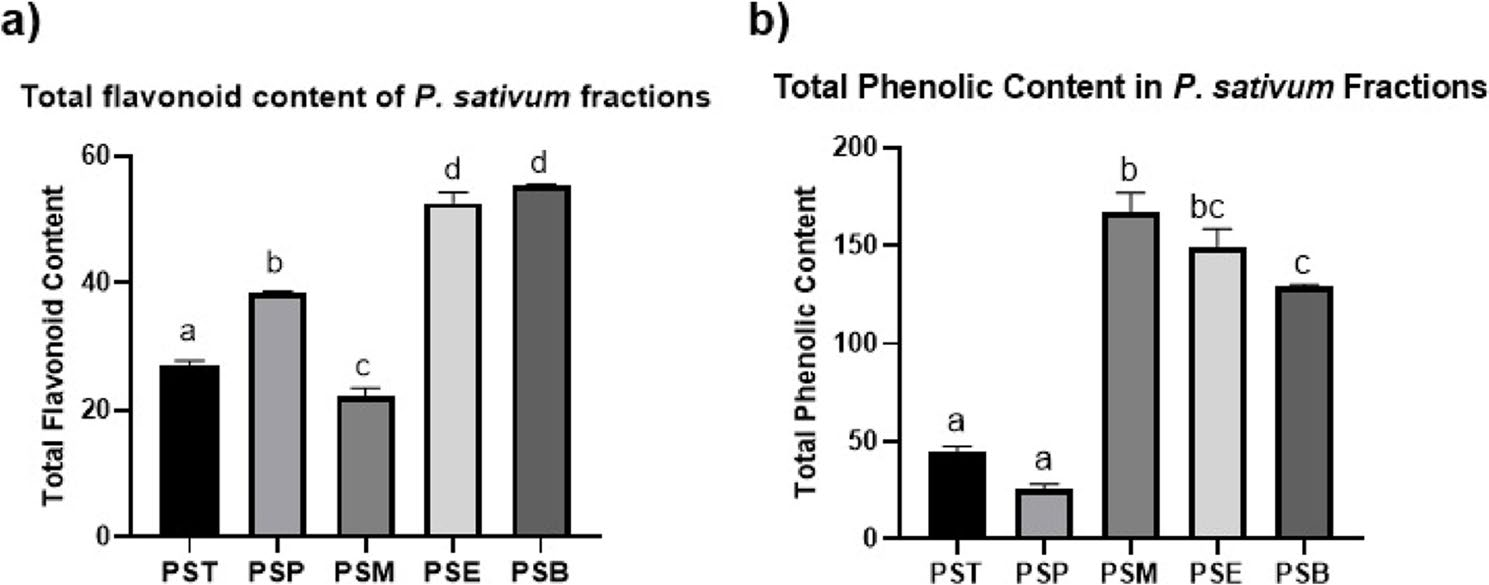
**a** Total flavonoid content, and (**b**) Total phenolic content in different fractions of *Pisum sativum*, PST: The total methanolic extract, PSP: petroleum ether fraction, PSM: methylene chloride fraction, PSE: ethyl acetate fraction, PSB: n-butanol fraction. Data are presented as mean ± SD (n=3). Different letters above bars indicate significant differences between fractions tested by one-way ANOVA followed by Tukey’s multiple comparisons test, (p < 0.05)

Phenolics and flavonoids are widely recognized for their potent antioxidant, antimicrobial, and anti-inflammatory properties [21]. They often work synergistically to enhance the overall anti-inflammatory activity of plant extracts, making their quantification vital for understanding and optimizing the plant activity [22]. Therefore, total flavonoid and phenolic contents were measured in the *P. sativum* fractions to explore their relation to the observed biological activity. The PSB fraction had the highest total flavonoid content (55.38 ± 0.24 mg quercetin Equivalents (QE)/g), closely followed by PSE (52.62 ± 1.71 mg QE/g), with no significant difference between them (*P* = 0.1111), indicating that both fractions are flavonoid-rich. The PSP fraction exhibited the lowest flavonoid content (38.48 ± 0.24 mg QE/g). Regarding total phenolic content, PSM showed the highest level (167.44 ± 9.43 mg GAE/g), followed by PSE (149.7 ± 9.43 mg GAE/g), with no significant difference (*P* = 0.1410), indicating that both fractions are phenolic-rich (Table S2 and Fig. 1). As expected, the PSP fraction displayed the lowest total phenolic content; however, it ranked third in total flavonoid content (38.48 ± 0.24 mg QE/g).

### Evaluation of the bioactivity of the obtained fractions

#### Immunomodulatory activity of *P. sativum* extract and fractions

**Fig. 2:**
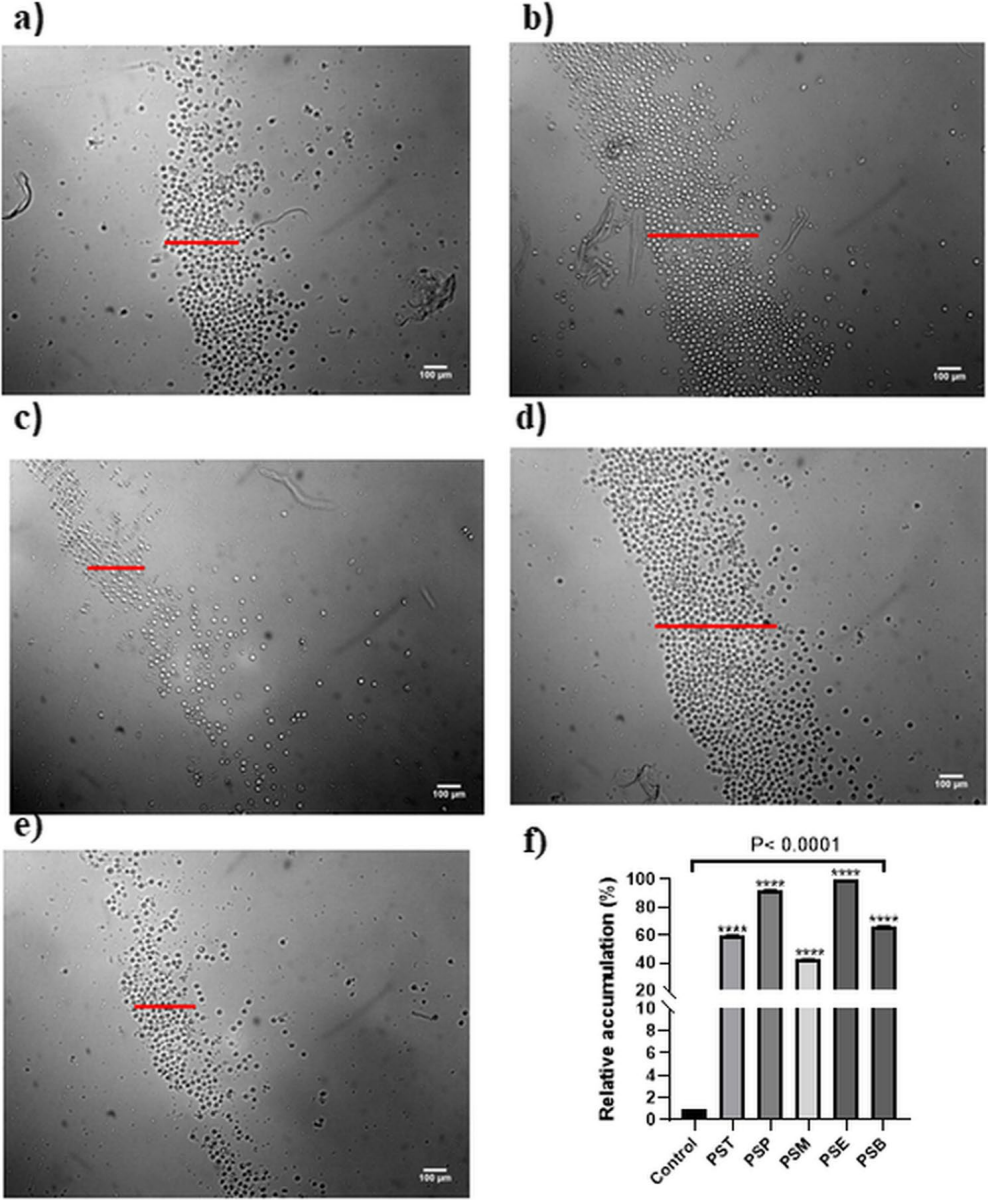
The effect of treatment of the peripheral blood mononuclear cells (PBMCs) for 24 h with 25 µg/mL of different fractions of *Pisum sativum* including (**a**) the total methanolic extract (PST), **b** petroleum ether fraction (PSP), **c** methylene chloride fraction (PSM), **d** ethyl acetate fraction (PSE), and (**e**) *n*-butanol fraction (PSB). The images were captured by inverted microscope at 10X magnification. **f** Relative leukocyte accumulation by different fractions of *Pisum sativum*, Data are presented as mean ± SD (*n* = 3). ****p < 0.0001, vs. control group. Statistical differences were assessed using one-way ANOVA followed by Dunnett’s multiple comparisons test versus control

The WST-1 assay showed that the PBMC (peripheral blood mononuclear cells) proliferation was found to be reduced by concentration over 50 µg/mL. On the other hand, the cells maintained their viability at 25 µg/mL. Hence, all the subsequent experiments were performed at this concentration. In the same context, the effects of different fractions obtained from *P. sativum* on leukocyte accumulation were evaluated using microscopic analysis. Among all the tested fractions, the ethyl acetate fraction (PSE) exhibited the highest leukocyte accumulation activity with a relative accumulation of 100% (accumulation score: 2.10), followed closely by the petroleum ether fraction (PSP) which demonstrated 92.4% relative accumulation (accumulation score: 1.94). The butanol fraction (PSB) showed moderate activity with 66.7% relative accumulation, while the total extract (PST) displayed slightly lower activity at 60.0% relative accumulation. The methylene chloride fraction (PSM) exhibited the least potent effect with 42.9% relative accumulation as depicted in Fig. 2. Collectively, leukocyte accumulation was increased after treating PBMCs with 25 µg/mL of PSE and PSP fractions for 24 h indicating an immune activity [23]. It is worth noting that increased leukocyte recruitment observed for PSE and PSP reflects immune activation that can contribute to the initiation of repair processes such as angiogenesis, re-epithelialization, and tissue remodeling [24, 25]. Once these cells have performed their functions, they are expected to undergo programmed cell death and are cleared by macrophages, preventing excessive or prolonged inflammation [26]. These results suggest that the bioactive compounds responsible for leukocyte accumulation are predominantly concentrated in PSE and PSP fractions. The observed activity of PSE fraction could be attributed to its high flavonoid and phenolic contents as determined by the total flavonoid and total phenolic content assays.

#### Anti-inflammatory effect of PSE and PSP fractions on LPS-stimulated RAW 264.7 cells

**Fig. 3:**
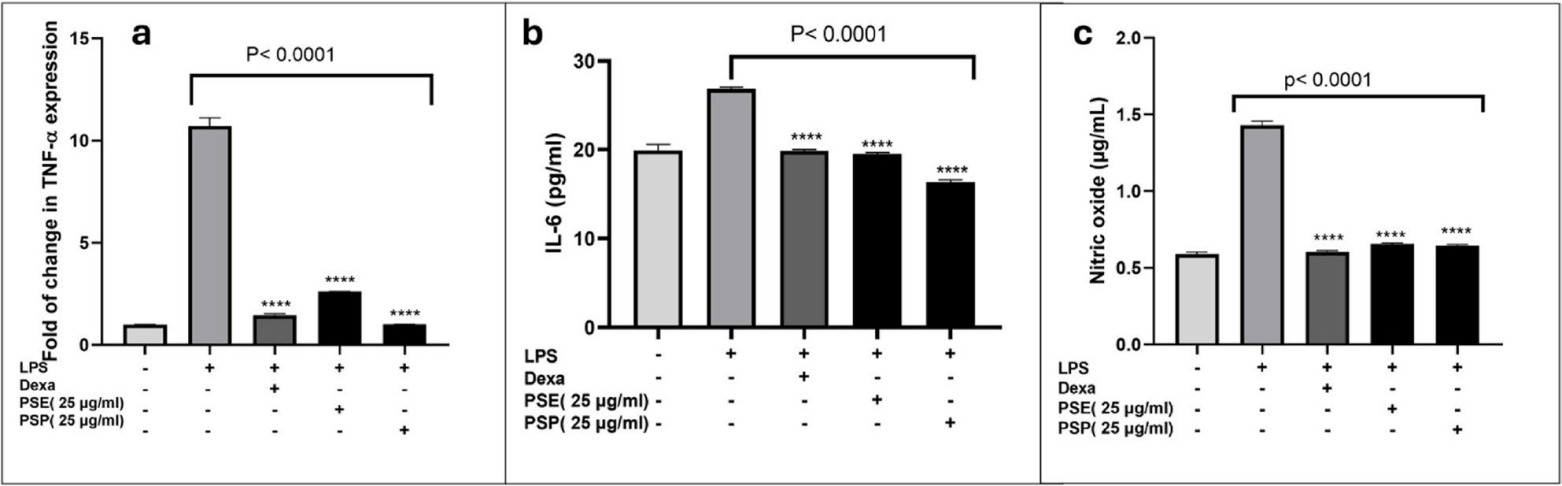
The effects of dexamethasone (Dexa), *P. sativum* ethyl acetate fraction (PSE), and *P. sativum* petroleum ether fraction (PSP) on LPS-induced inflammation of RAW 264.7 cells. **a** TNF-α gene expression; **b** IL-6 production; **c** nitric oxide (NO) production. Data are presented as mean ± SEM (*n* = 3). **** means *p* value < 0.0001 compared to LPS group. Statistical differences were assessed using one-way ANOVA followed by Dunnett’s multiple comparisons test versus control

Since PSE and PSP fractions exhibited the highest activity in leukocyte accumulation, their anti-inflammatory effects were evaluated in LPS-stimulated RAW 264.7 cells at a non-cytotoxic concentration of 25 µg/mL. Firstly, the ability of the tested fractions to downregulate gene expression of TNF-α was investigated. Following LPS treatment, TNF-α expression increased from 0.995 to 10.71042, representing a 10.75-fold increase. Remarkably, treatment with dexamethasone reduced TNF-α expression by 86.35% compared to LPS-treated cells. Similarly, PSP treatment resulted in an 87.23% reduction in TNF-α expression, while PSE treatment decreased TNF-α expression by 84.91%, as shown in Fig. 3a.

Furthermore, the production levels of IL-6 and NO were measured. In the case of IL-6, LPS-stimulation increased its production to 26.85 pg/mL. Treatment with 1 µM dexamethasone reduced IL-6 production to 19.95 pg/ml, a 25.6% decrease, bringing levels closer to those observed in the non-treated DMSO control cells. Similarly, treatment with 25 µg/mL of PSE resulted in a 27.3% reduction, lowering IL-6 production to 19.52 pg/mL. The most significant reduction was observed with 25 µg/mL of PSP, which decreased IL-6 production by 38.9%, reducing its level to 16.37 pg/mL. These results demonstrate that both PSE and PSP effectively reduce IL-6 production compared to dexamethasone in LPS-stimulated cells, with PSP exhibiting the strongest inhibitory effect, nearly restoring IL-6 to baseline, as shown in Fig. 3b.

The Griess assay showed that untreated cells produced 0.59 µg/mL nitric oxide. Upon LPS stimulation, NO production increased by 142.37%, reaching 1.43 µg/mL, which is equivalent to approximately 47.6 µM. Treatment with 1 µM dexamethasone, 25 µg/mL PSE, or 25 µg/mL PSP significantly reduced NO production to 0.605 µg/mL, 0.655 µg/mL, and 0.645 µg/mL, respectively. These reductions correspond to 57.69%, 54.2%, and 54.9% decreases, respectively, compared to the LPS-treated group, as depicted in Fig. 3c. The results obtained from this study together with the increased leukocyte recruitment indicate that PSE and PSP fractions possess immunomodulatory and anti-inflammatory properties.

### Chemical investigation and isolation of major compounds from the bioactive fractions of *Pisum sativum* L.

PSP and PSE fractions of the aerial parts of *P. sativum* L., showing the highest anti-inflammatory and immunomodulatory activities, were further analyzed by chromatographic isolation and chemical composition techniques. The saponifiable matter in the petroleum ether fraction was analyzed using GC-MS, while LC-MS/MS was employed for the metabolic profiling of the ethyl acetate fraction. Major compounds from both fractions were purified by column chromatography, yielding compounds (1–7, Scheme 1**)**.

#### GC-MS analysis of the saponifiable matter in the petroleum ether fraction of *P. sativum*

PSP showed significant anti-inflammatory activity by enhancing leukocyte accumulation, suppressing TNF-α expression, and reducing NO and IL-6 levels. These findings prompted further investigation into the chemical composition of the PSP fraction. Thus, GC-MS analysis was conducted to examine the saponified portion of the fraction. Three major components, accounting for 63.73% of the total fatty acid methyl esters (FAMEs) in the tested fraction, were tentatively identified. Their predominance was evident from their characteristic peaks in the GC-MS total ion chromatogram at retention times of 16.15, 20.05, and 20.22 min (Fig. S3a). Saturated fatty acids accounted for 22.26%, while unsaturated ones made up 41.47% of the total identified FAMEs. The major identified compounds were the methyl esters of hexadecanoic acid (palmitic acid, 22.26%), linoleic acid (11.89%), and linolenic acid (29.58%) (Table S3). Their tentative identification was based on comparing their relative retention times and fragmentation patterns (Figs. S3b-d) with those available in the NIST and WILEY mass spectral libraries. The presence of these major components has already been reported in the seed oils of *P. sativum*, lending further support to their identification in our study [27]. It is worth noting that the tentatively identified linolenic acid may correspond to either of the two positional isomers: α-linolenic acid (9,12,15-octadecatrienoic acid, ALA) or g-linolenic acid (6,9,12-Octadecatrienoic acid, GLA), both of which have been previously reported in *P. sativum* [27]. The high proportion of unsaturated fatty acids (41.47%) in the saponifiable matter, including α-linolenic acid (ALA), γ-linolenic acid (GLA), and conjugated linoleic acid (CLA), suggests a potential contribution to the observed anti-inflammatory and immunomodulatory effects, which are crucial for regulating the early inflammatory phase of wound healing [28]. Specifically, α-linolenic acid (ALA) exerts potent anti-inflammatory and immunomodulatory effects that contribute to tissue repair and wound healing. In murine RAW 264.7 macrophages, ALA was reported to downregulate key inflammatory markers, including NO, iNOS, COX-2, and TNF-α, through the inhibition of NF-κB and MAPK signaling [29]. Similarly, in human corneal epithelial (HCE) cells, it reduced TNF-α, IL-6, IL-1β, and IL-8, demonstrating its role in controlling inflammatory responses [30]. Beyond these effects, ALA also supports wound healing, particularly in conditions of impaired tissue repair, by promoting tissue regeneration, improving scar quality, and contributing to processes related to nerve recovery at the wound site [31]. Collectively, these findings highlight ALA’s multi-faceted role in skin repair and its potential therapeutic relevance in enhancing tissue healing and regeneration. Similarly, dietary supplementation with CLA was reported to enhance early wound closure in cutaneous wound models by regulating oxidative stress and inflammatory responses [32]. Linolenic fatty acids, in general, have been documented to modulate nitric oxide production, cytokine release, and immune cell function, thereby contributing to an environment conducive to tissue regeneration and effective wound repair [28].

#### LC-ESI-MS/MS analysis of the ethyl acetate fraction of *P. sativum* L. (PSE)

**Fig. 4:**
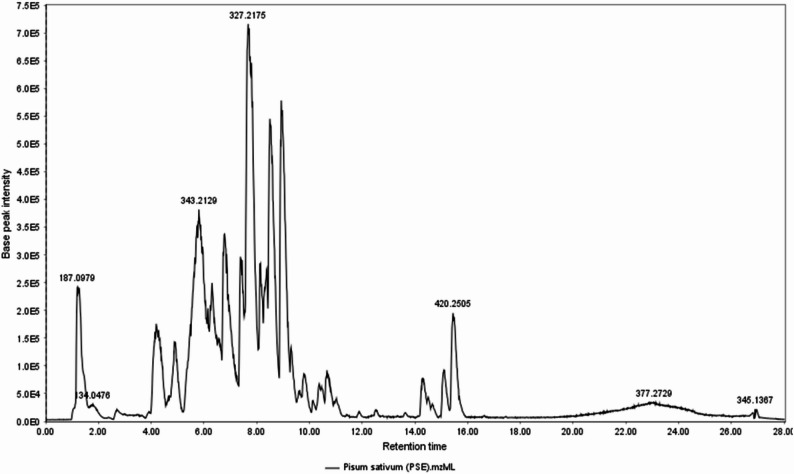
Base peak chromatogram from LC-MS/MS analysis of the ethyl acetate fraction of *P*isum *sativum* L. (PSE) in negative ESI mode

Liquid chromatography-tandem mass spectroscopy (LC-MS/MS) technique was used for the metabolic profiling of the ethyl acetate fraction of *P. sativum* (PSE) to tentatively identify the secondary metabolites potentially associated with its observed anti-inflammatory activity. The analysis was done using electrospray ionization (ESI) in negative ion mode **(**Fig. 4**)** due to the high phenolic content of the examined fraction. It led to tentative identification of 42 secondary metabolites of various chemical classes. These tentatively identified compounds can be broadly categorized into flavonols, flavones, flavanones, isoflavones, anthocyanidins, flavanols, simple phenols, and phenolic acids. The molecular formula, mode of ionization, retention time, mass error (*≤* 10 ppm), as well as bibliographic references of each phytochemical are presented in Table S4. Furthermore, the ESI-MS/MS fragmentation spectra supporting the tentative identification of these metabolites are provided in the supplementary information (Figs. S4 - S45).

**Fig. 5:**
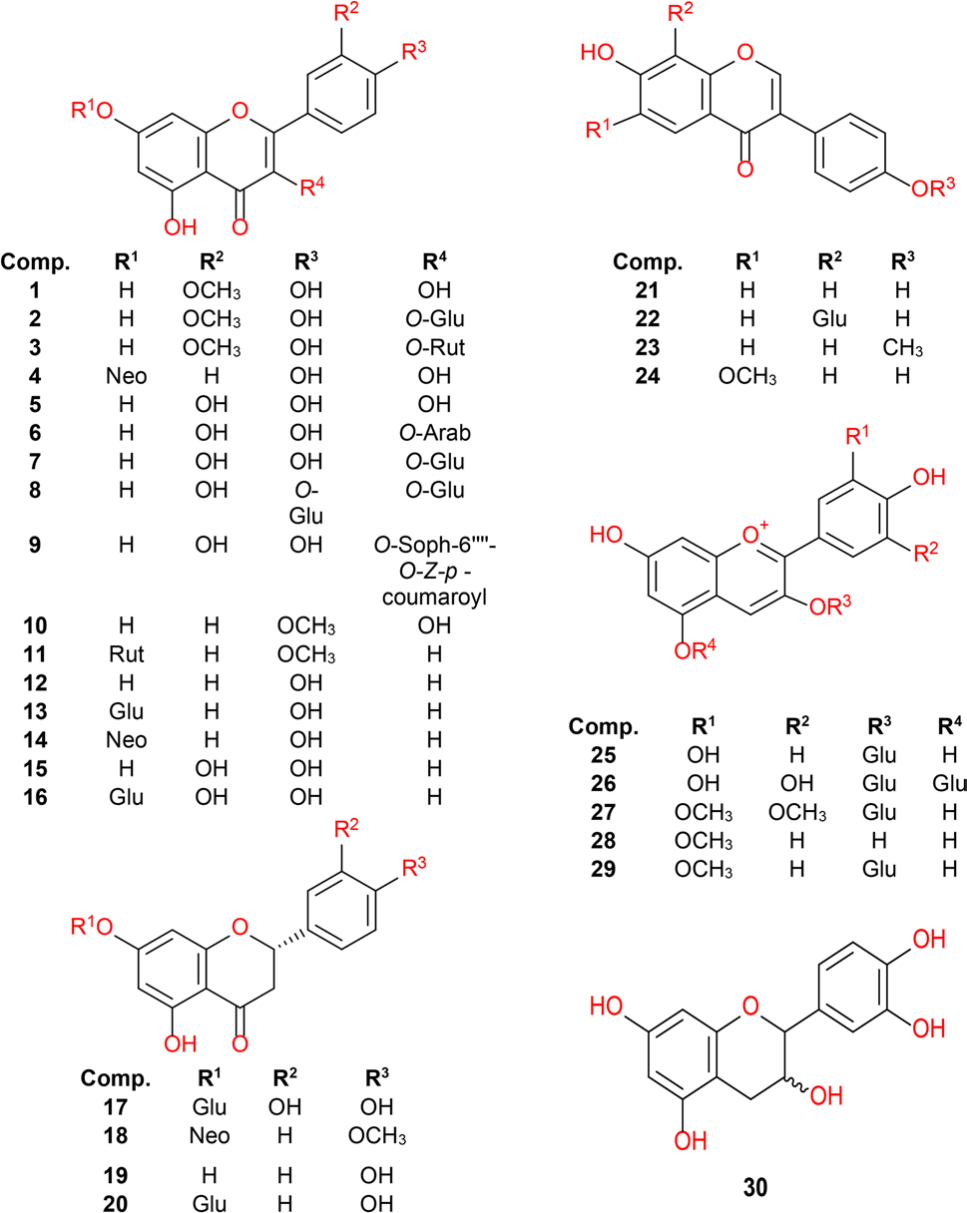
Structures of flavonols, flavones, flavanones, isoflavones, anthocyanidins, flavanols, and their glycosides tentatively identified by LC-MS/MS analysis in the ethyl acetate extract of *Pisum sativum* L. (Arab = arabinose, Glu = glucose, Neo = neohesperidoside, Rut = Rutinoside, Soph = sophorotrioside)

The majority of the tentatively identified compounds (30 out of 42) belong to the flavonoid family (Fig. 5; Table S4), which is one of the largest and most studied groups of plant secondary metabolites. The study identified multiple flavanols, including isorhamnetin (1–3), kaempferol (4), and quercetin derivatives. Quercetin derivatives (5–9) comprised the highest number of the identified flavonols. Their structural diversity is attributed to different glycosidic linkages, hydroxylation, and methylation patterns. Flavones such as apigenin (12), luteolin (15), and acacetin glycosides were tentatively identified. The analysis also revealed various flavanones such as naringenin (19), eriodyctyol, and isosakuranetin glycosides. Daidzein (21), its 8-*C*-glucoside (22), along with formononetin (23), and glycitein (24) comprised the identified isoflavones which are characteristic of the Fabaceae family [33]. Five anthocyanidins including cyanidin, malvidin and peonidin derivatives (e.g., cyanidin-3-*O*-glucoside (25), Cyanidin-3,5-di-*O*-glucoside (26), peonidin-3-*O*-glucoside (29) were detected, along with one flavanol, possibly catechin or epicatechin (30).

Interestingly, the flavonoids annotated in PSE fraction can be linked to the significant anti-inflammatory and immunomodulatory activities, as supported by several previous in vitro and in vivo studies. Moreover, many of these flavonoids also exhibit wound-healing potential in experimental models, suggesting additional benefits beyond inflammation suppression. For instance, quercetin (5) has been shown to inhibit the production of NO, prostaglandin E2 (PGE2), and key pro-inflammatory cytokines such as TNF-α, interleukin-1β (IL-1β), and interleukin-6 (IL-6) in lipopolysaccharide (LPS)-stimulated macrophages (RAW 264.7) [34, 35]. It has also been reported to promote wound healing by enhancing fibroblast proliferation, collagen deposition, angiogenesis, and re-epithelialization through activation of the Wnt/β-catenin signaling pathway [36]. Similarly, cyanidin-3-glucoside (25) has demonstrated the ability to suppress NO production and significantly reduce TNF-α and IL-6 expression in THP-1 macrophage cells [37, 38]. Moreover, it promotes wound healing by enhancing fibroblast and keratinocyte migration, stimulating VEGF production, and preventing excessive inflammation through inhibition of ROS accumulation and NF-κB activation, indicating its potential role in tissue repair and regeneration [39]. Apigenin, luteolin, and kaempferol are reported to contribute to inflammation suppression by downregulating IL-6, TNF-α, IL-1β, inducible nitric oxide synthase (iNOS), and cyclooxygenase-2 (COX-2) expression in macrophage cells and other inflammatory models [40–42]. In addition, they enhance wound healing: apigenin was reported to accelerate excisional wound closure, promotes macrophage M2 polarization, and increases endothelial cell migration and VEGF secretion [43]; luteolin improved epithelial regeneration, granulation tissue formation, and angiogenesis in topical wound models [44]; and kaempferol promoted collagen deposition, re-epithelialization, angiogenesis, antioxidant defense, and tensile strength in both diabetic and nondiabetic wound models [45]. Isorhamnetin and acacetin have also been reported to significantly inhibit TNF-α, IL-1β, and IL-6 secretion in both in vitro and in vivo studies, supporting their potential role in mitigating inflammation-related conditions [46, 47]. Additionally, naringenin reduced pro-inflammatory cytokines such as TNF-α and IL-6 in immune cells and promoted wound healing by decreasing inflammation and supporting faster tissue repair [48, 49]. These findings confirm the strong anti-inflammatory and immunomodulatory effects of the PSE fraction. Furthermore, the combined presence of these flavonoids may provide complementary or synergistic actions, contributing to enhanced wound healing and tissue repair.

**Fig. 6:**
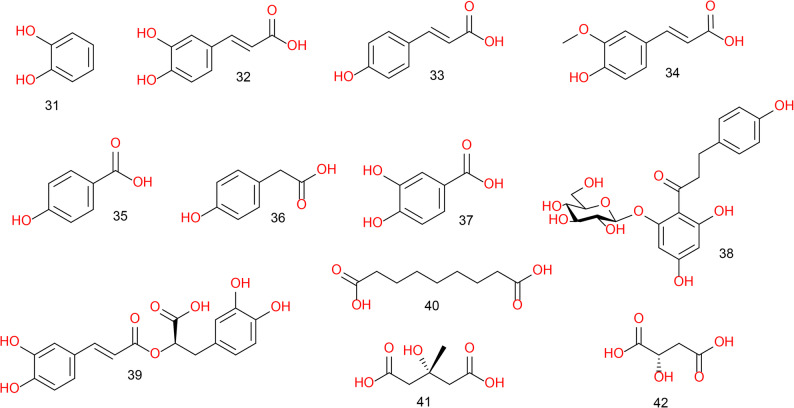
Structures of phenolics, and aliphatic acids tentatively identified by LC-MS/MS analysis in the ethyl acetate fraction of *Pisum sativum* L

Furthermore, LC–MS/MS profiling revealed the presence of various phenolic compounds (Fig. 6; Table S4), including catechol (31), hydroxycinnamic acids like caffeic acid (32), *p*-coumaric acid (33), and ferulic acid (34), and hydroxybenzoic acids such as *p*-hydroxybenzoic acid (35), *p*-hydroxyphenyl acetic acid (36), and protocatechuic acid (37). Additionally, other phenolic derivatives including phloridzin (38), and rosmarinic acid (39) were identified. In addition, this analysis tentatively identified various aliphatic acids represented by azelaic acid (40), 3-hydroxy-3-methylglutaric acid (41), and D- (+)-malic acid (42), (Fig. 6; Table S4).

#### Characterization of the pure compounds isolated from the petroleum ether (PSP) and ethyl acetate (PSE) fractions of Pisum sativum L.

**Fig. 7:**
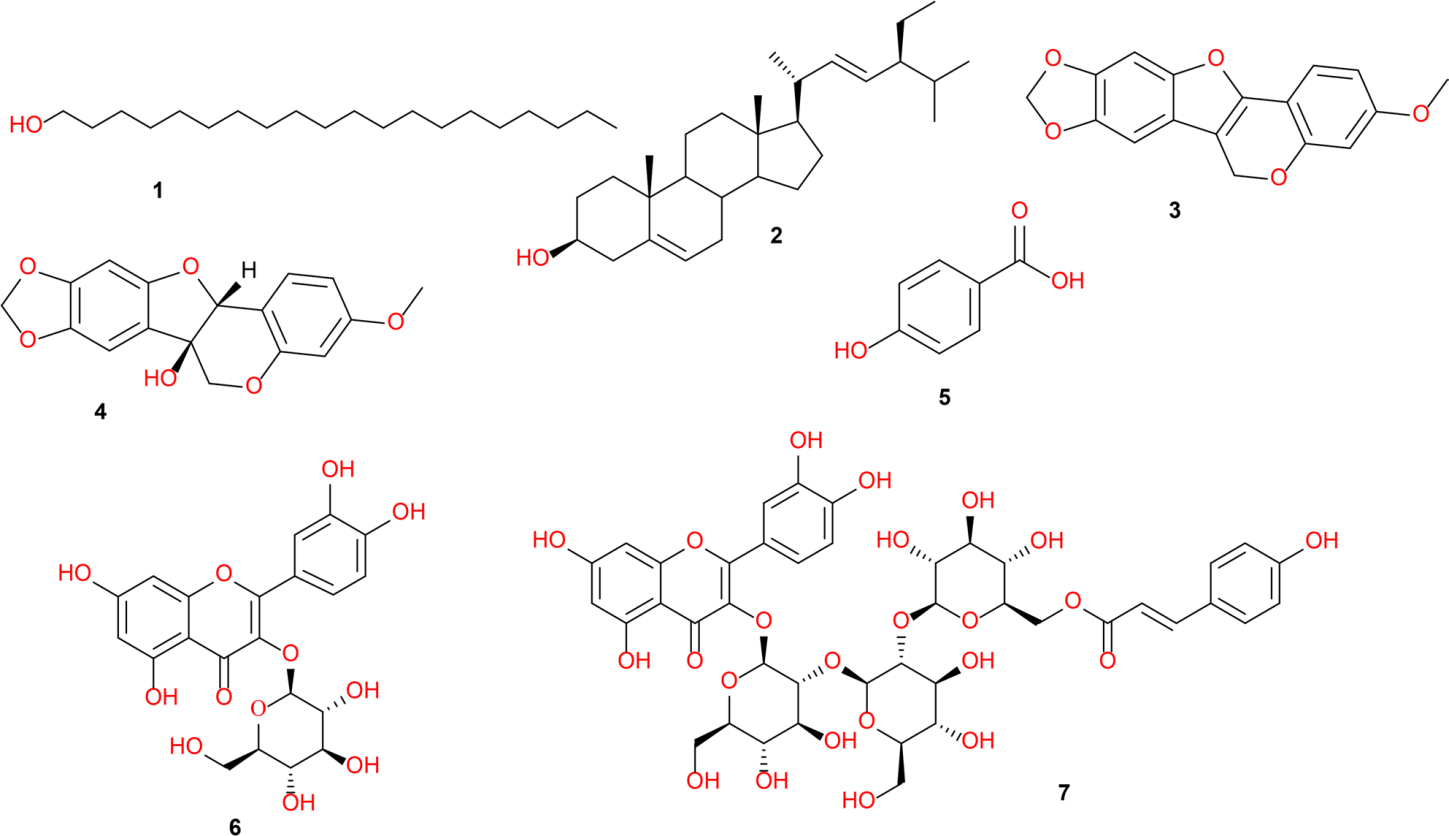
Structures of the compounds isolated from the petroleum ether and ethyl acetate fractions of *Pisum sativum*, and fully characterized by spectroscopic analysis, 1-eicosanol (1), stigmasterol (2), anhydropisatin (3), pisatin (4), *p*-hydroxy benzoic acid (5), quercetin-3-*O*-*β*-D-glucopyranoside (6), and quercetin-3-*O*-(6’’’’-*O*-*cis*-*p*-coumaroyl)-sophorotrioside (7)

The petroleum ether and ethyl acetate fractions demonstrated the highest immunomodulatory activity, leading to their selection for the isolation of bioactive compounds. Consequently, seven distinct compounds were isolated (Fig. 7). The chemical structures of these compounds were fully characterized and elucidated based on their spectroscopic data (Figs. S46-S76) and comparison with literature. Following saponification of the petroleum ether extract, the chromatographic separation of the unsaponified portion resulted in the isolation of 1-eicosanol (1) [50], stigmasterol (2) [51], and anhydropisatin (3) [52]. Additionally, pisatin (4) [53], was identified in the unprocessed petroleum ether fraction, suggesting the dehydration of pisatin to anhydropisatin probably due to thermal treatment and liberation of fatty acids during the saponification reaction [54]. Meanwhile, the ethyl acetate fraction (PSE) yielded three compounds: *p*-hydroxybenzoic acid (5) [55], quercetin-3-*O*-*β*-D-glucopyranoside (6) [56], and quercetin-3-*O*-(6’’’’-*O*-*cis*-*p*-coumaroyl)-sophorotrioside (7) [57]. Compounds 5–7, isolated from PSE and fully characterized, were also detected in the metabolomic LC–MS/MS analysis of the same fraction, indicating their relatively high abundance in PSE.

### Evaluation of the immunomodulatory activity of pure compounds 3, 4, 6, and 7 isolated from PSP and PSE fractions of *P. sativum*

#### Impact on cell viability, IL-6 secretion, and reactive oxygen species (ROS) generation in human monocytes

Given the significant immunomodulatory and anti-inflammatory effects of the PSE and PSP fractions, further biological investigations were conducted to evaluate the activities of their main isolated compounds (3, 4, 6, and 7) and determine their contribution to the observed effects. The MTS assay revealed that the tested compounds had no significant impact on the viability of human monocytes at concentrations up to 10 µM, suggesting a favorable safety profile at these levels. However, at 20 µM, only Compound 4 exhibited notable cytotoxic effects (Fig. S77). Consequently, all subsequent experiments were conducted using human monocyte cells treated with 5 and 10 µM concentrations.

**Fig. 8:**
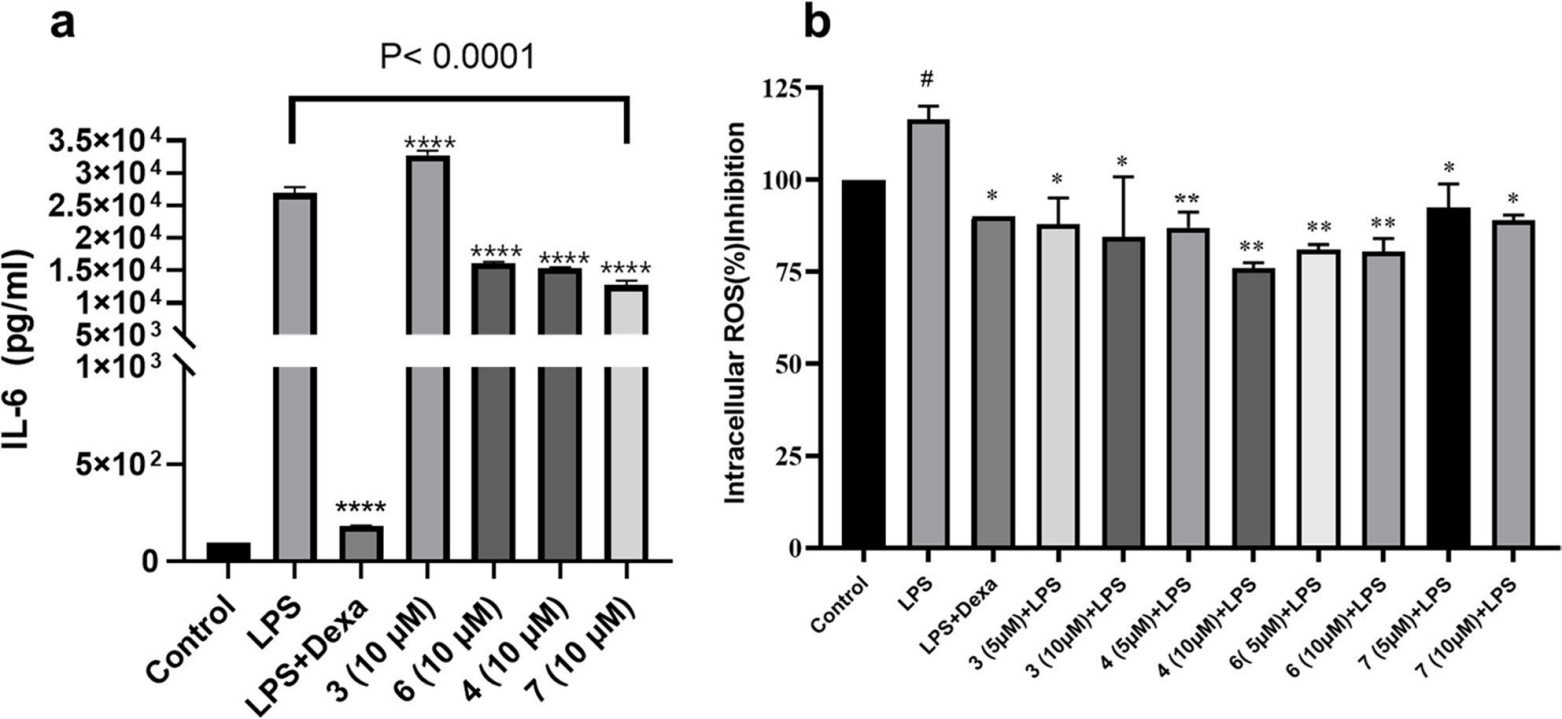
Effect of compounds 3, 4, 6, 7 and dexamethasone (Dexa) on LPS-stimulated human monocytes. **a** IL-6 production levels in culture supernatants after treatment with LPS alone or in combination with (DEXA) or tested compounds (10 µM). Compounds 4, 6, 7, and Dexa significantly reduced IL-6 levels, whereas compound 3 significantly enhanced IL-6 production. **b** Intracellular ROS levels after treatment with LPS alone or in combination with tested compounds at indicated concentrations. Data are presented as mean ± SEM (*n* = 3). #*p* < 0.05 vs. control: *p < 0.05, **p < 0.01 and ****p < 0.0001, vs. LPS group. Statistical differences were assessed using one-way ANOVA followed by Dunnett’s multiple comparisons test versus control

LPS significantly induced IL-6 secretion in human monocytes, increasing its level from 0.42 pg/mL in untreated control cells to 113.1 pg/mL. Among the tested compounds, Compound 7 was the most effective inhibitor, reducing IL-6 levels by 47.1%, from 113.1 pg/mL to 53.2 pg/mL. Compound 4 was the second most effective, lowering IL-6 to 64.8 pg/mL, a reduction of 42.7%. Compound 6 showed a moderate effect, decreasing IL-6 levels to 68.3 pg/mL (39.6% reduction), making it less effective than Compounds 7 and 4. In contrast, Compound 3 increased IL-6 levels to 137.4 pg/mL, suggesting a stimulatory effect rather than inhibition. These results highlight Compound 7 as the strongest IL-6 inhibitor, followed by Compound 4 and Compound 6, while Compound 3 appears to enhance IL-6 production as illustrated in Fig. 8a. It is worth noting that IL-6 is a pivotal cytokine in wound healing, playing a dual role in orchestrating the early inflammatory response while supporting tissue repair processes such as keratinocyte proliferation, re-epithelialization, and angiogenesis [24, 25]. While excessive IL-6 can lead to chronic inflammation and impaired repair, its controlled regulation is essential for efficient wound healing [26, 58, 59]. Our observations suggest that compounds 4, 6, and 7 modulate IL-6 signaling, restraining excessive pro-inflammatory activity while allowing early immune-mediated repair processes. This highlights the modulatory, rather than strictly inhibitory role of IL-6, consistent with its well-established dual function in wound healing.

Results indicated that Compound 4 (10 µM) was the most effective in reducing intracellular ROS levels, achieving a 40.5% reduction, making it the most potent inhibitor among the tested compounds. Followed closely by compound 6, decreasing ROS levels by 35.5%. Compound 3 also exhibited a strong inhibitory effect, reducing ROS levels by 32% at 5 µM and 28.5% at 10 µM. While, Compound 7 showed the weakest effect, lowering ROS levels by 27.5% at 10 µM and 20% at 5 µM. The results are illustrated in Fig. 8b.

#### Impact on cell viability and wound healing of HaCaT Keratinocytes

The ability of the tested compounds to modulate the key markers, ROS and IL-6, prompted us to evaluate their potential to enhance wound healing. To assess this, a scratch assay was performed using the HaCaT keratinocyte cell line, along with a crystal violet assay to measure cell proliferation. Notably, all the tested compounds demonstrated proliferative activity in keratinocytes across a concentration range of 0.5 to 80 µM, suggesting both their safety and their potential to promote cell proliferation (Fig. S79).

**Fig. 9:**
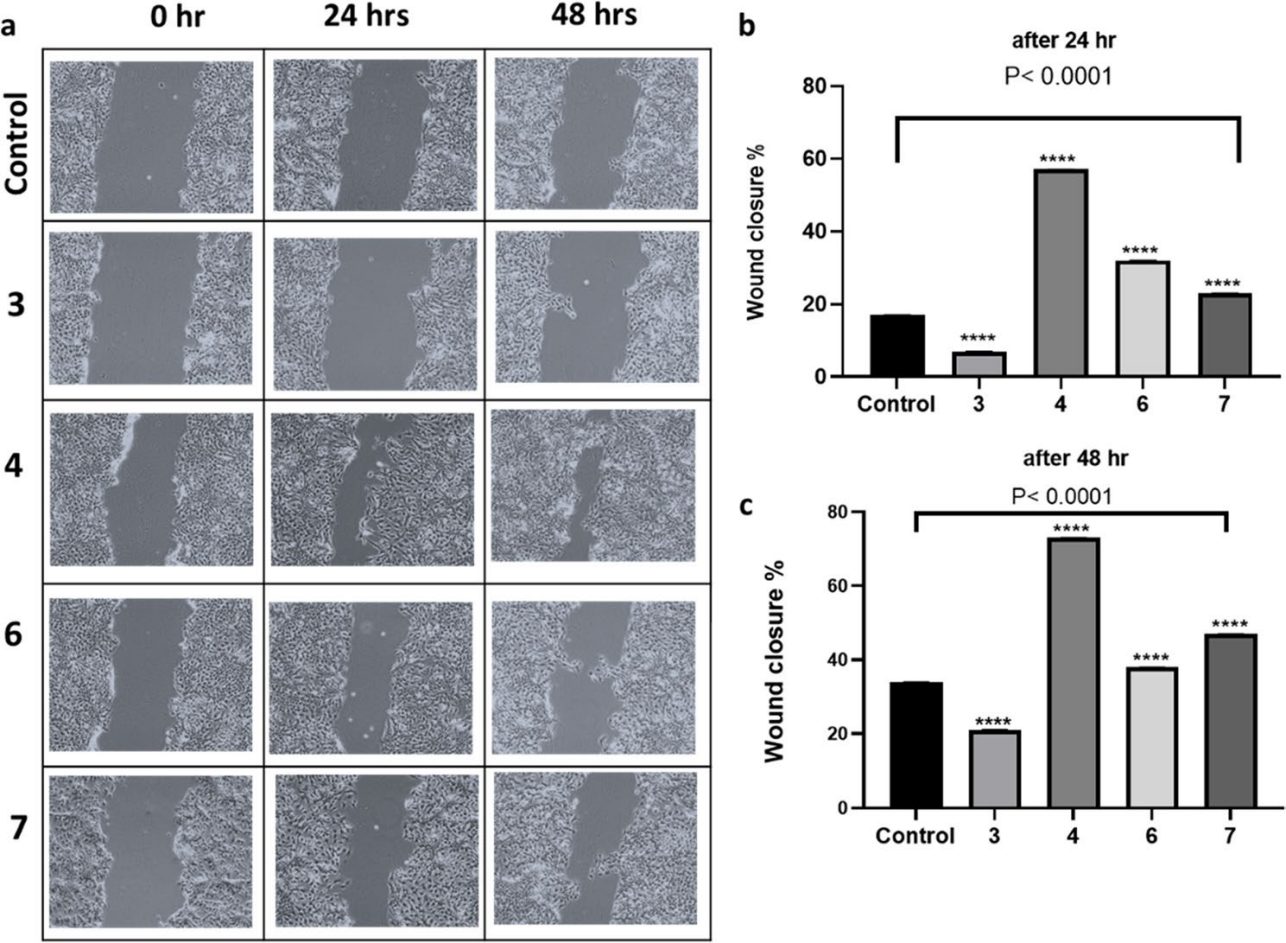
**a** Effect of compounds on keratinocyte wound healing in a scratch assay model. Representative phase-contrast images of HaCaT keratinocytes at 0-, 24-, and 48-hours post-scratch treatment with different compounds. The images were captured at 10X magnification. **b** percentage of wound closure at 24. **c** percentage of wound closure at 48 h for each treatment condition, normalized to the untreated control. Data are presented as mean ± SEM (*n* = 3). ****p < 0.0001, vs. control group. Compounds 4, 6 and 7 significantly improved wound closure, whereas compound 3 significantly impaired wound healing. Statistical differences were assessed using one-way ANOVA followed by Dunnett’s multiple comparisons test versus control

Following wound induction, the percentage of wound closure for each compound was assessed at 24 and 48 h in comparison to untreated controls. After 24 h, most compounds significantly enhanced wound healing, with compound 4 demonstrating the strongest effect (Fig. 9a), increasing wound closure by 57%, followed by compound 6 (32%) and compound 7 (23%). In contrast, compound 3 exhibited an inhibitory effect, reducing wound healing by 7% (Fig. 9b). By 48 h, the healing effects became more pronounced. Compound 4 continued to show the greatest enhancement, increasing wound closure by 73%, followed by compound 7 (47%) and compound 6 (38%). Notably, although compound 3 initially impaired wound healing, it exhibited a modest improvement of 21% at the 48-hour mark (Fig. 9c**)**. The behavior of compound 3 could be explained in the light of its ability to increase the levels of IL-6 in contrast to other compounds which may lead to sustained inflammatory response halting the wound repair mechanisms in the cells [60]. These findings suggest that with the exception of compound 3, the tested compounds, particularly compound 4, may serve as promising agents for promoting wound healing. Notably, pisatin, the most active compound isolated from the aerial parts of *P. sativum*, is reported here for the first time to promote wound healing in keratinocytes.

Interestingly, compound 3 (anhydropisatin) displayed markedly different behavior from compound 4 (pisatin), despite the sole structural difference being the presence of a double bond. Historically, this class of compounds had limited pharmacological application due to concerns about mitogenic activity [61, 62]. Our observations suggest that while pisatin may have potential applications in wound healing, anhydropisatin reduces keratinocyte proliferation. This finding indicates that anhydropisatin could have therapeutic implications for conditions characterized by keratinocyte hyperproliferation, such as psoriasis and lichen planus.

No prior studies have specifically examined the effects of compounds 3 and 4 on human keratinocytes. However, quercetin, the primary scaffold of compounds 6, and 7, has been documented to reduce inflammation and promote wound healing in vitro, particularly in models of atopic dermatitis-induced skin inflammation [63]. Additionally, quercetin has been identified as a key component responsible for the wound healing properties of *Sedum telephium* L. leaves [64].

### In-silico prediction of pisatin molecular targets and molecular docking analysis

#### Molecular target prediction and network pharmacology

Network Pharmacology has been utilized extensively to identify molecular targets of natural products [65]. In this study, it was employed to predict the molecular targets of pisatin associated with its wound-healing activity. A total of 396 potential targets for pisatin were identified, with the top 115 selected for network pharmacology analysis. Simultaneously, 473 wound-healing-related targets were retrieved from GeneCards. Using the Venny tool, 26 common targets were identified and selected for enrichment analysis (Fig. S80).

This enrichment analysis identified positive regulation of the response to external stimuli as the most significantly enriched pathway (Fig. S80b). This pathway plays a crucial role in enhancing cellular sensitivity to growth factors and chemokines, promoting the recruitment of immune cells and fibroblasts to the wound site. Additionally, the positive regulation of epithelial cell migration highlights the essential role of re-epithelialization, where keratinocytes must migrate effectively to restore the epidermal barrier and accelerate wound closure. The top enriched wound-healing targets, ranked by enrichment score, are SRC, VEGFA, JAK2, mTOR, and KDR. SRC, a non-receptor tyrosine kinase, regulates cell adhesion, migration, and proliferation, promoting keratinocyte movement, fibroblast activation, and extracellular matrix remodeling, all essential for tissue repair [66]. VEGFA is a key driver of angiogenesis, ensuring oxygen and nutrient supply, particularly in chronic wounds with poor vascularization [67]. JAK2, a major component of the JAK/STAT pathway, modulates cytokine-driven inflammation and has been linked to chronic wound healing, especially via photobiomodulation [68]. MTOR governs cell proliferation, protein synthesis, and metabolism, supporting fibroblast function, extracellular matrix production, and angiogenesis, particularly under hypoxic and oxidative conditions [69]. KDR (also known as VEGFR-2) is crucial for endothelial recovery, mediating angiogenesis and vascular remodeling to promote effective revascularization and tissue oxygenation [70].

Cellular component (CC) analysis (Fig. S80) showed significant enrichment of the PI3K complex (class IB), cytoplasmic vesicle lumen (*p* = 0.008), and membrane raft. These components are critical for processes such as cell survival, proliferation, migration, and vesicle-mediated cytokine secretion, all of which are essential for tissue repair. Notably, enriched targets like SRC, JAK2, PIK3CG, mTOR, and F2 indicate that pisatin influences vital intracellular signaling and membrane organization. In parallel, molecular function (MF) analysis (Fig. S80) identified metalloendopeptidase activity, protein tyrosine kinase activity, and growth factor receptor binding as top functions. These activities support extracellular matrix remodeling, cell migration, and re-epithelialization, with key targets including SRC, VEGFA, PIK3CA, and MMP8. To further explore the pathways involved in pisatin’s wound-healing effects, a protein-protein interaction (PPI) network was constructed using a confidence score threshold of 0.7 to ensure the reliability and relevance of the identified connections. Analysis of the network underscored the central role of the phosphatidylinositol 3-kinase (PI3K) pathway in mediating these interactions (Fig. S81), solidifying its importance in pisatin’s therapeutic mechanism for wound healing.

Our study is the first to demonstrate the wound-healing activity of pisatin, a pterocarpan structurally related to flavonoids, and to predict PI3K as potential target for its activity. These findings align with previous research highlighting the well-established wound-healing properties of flavonoids, which may act through the PI3K/Akt signaling pathway to promote key processes of cell survival, proliferation, and angiogenesis that are required for effective tissue repair [71, 72]. Studies on *Calendula officinalis* tincture, rich in flavonol glycosides, demonstrate that these compounds stimulate fibroblast proliferation and migration via a PI3K-dependent pathway [73]. Recent findings indicate that flavonoids play a positive role in managing diabetic wounds by regulating MMP-8 and PI3K/Akt pathways, suggesting their potential as therapeutics to prevent the devastating effects of diabetic wounds [74].

#### Molecular docking analysis

Given the central role of PI3K in the wound repair process, the potential interaction between pisatin and this key molecular target was investigated. A molecular docking study was conducted to evaluate the binding affinity and interaction patterns between pisatin and PI3K. PI3K contains several active sites (Fig. S82), and the specific binding site of a compound determines its effect on PI3K activity. Site 1, the ATP-binding site, governs the catalytic function of PI3K, with compounds targeting this site typically acting as inhibitors [75]. In contrast, Site 2 is located at the interface between the catalytic subunit p110α and the regulatory subunit p85α. Binding at this site can disrupt their interaction, enhancing enzymatic activity and promoting the membrane recruitment of p110α, thereby activating the PI3K-associated cell proliferation pathway [76].

**Fig. 10:**
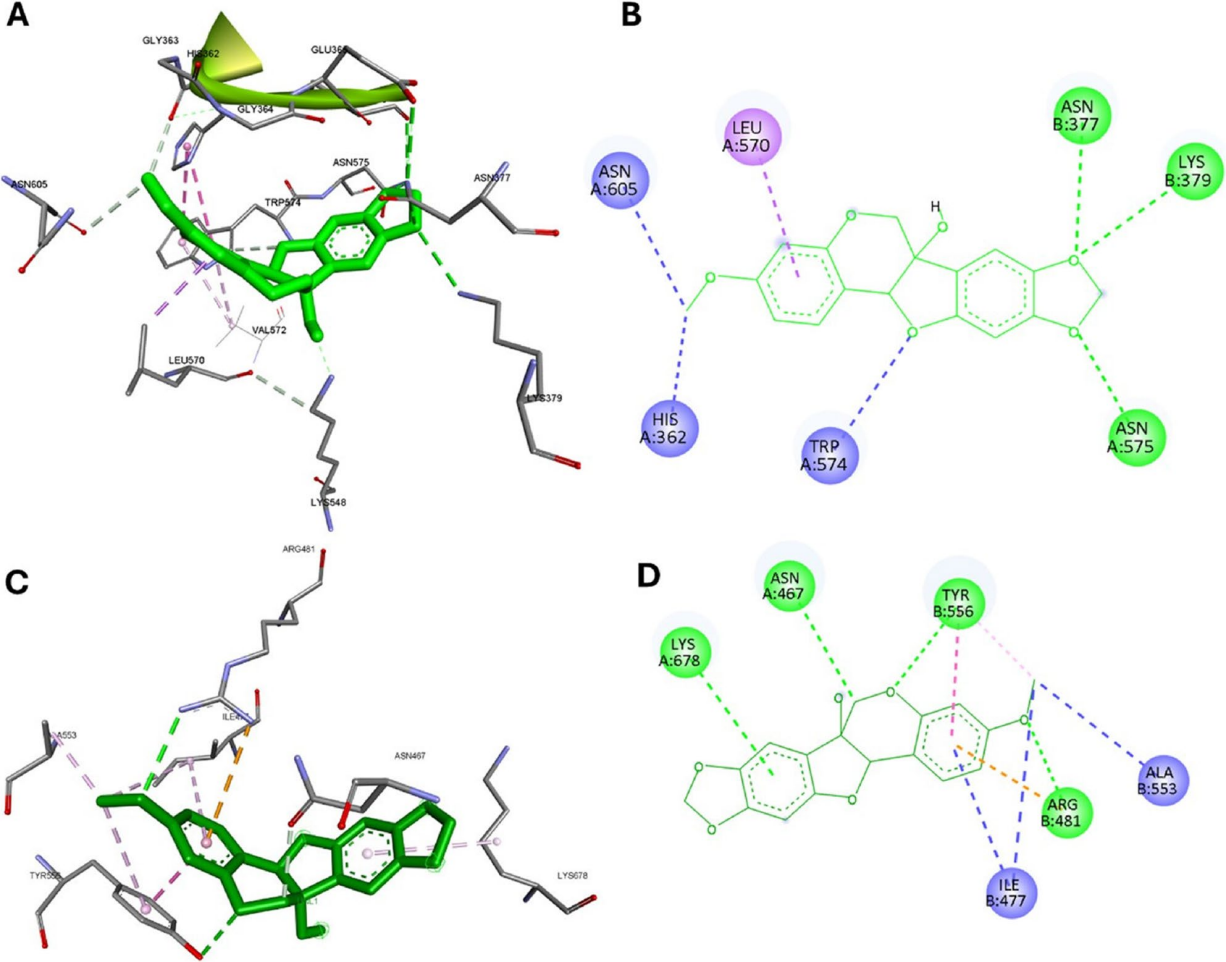
Molecular interactions of pisatin with the PI3K complex (PDB: 3HHM). **a** Three-dimensional (3D) representation of pisatin (green) bound to position-1 of site-2 in the PI3K complex. **b** Two-dimensional (2D) interaction diagram of pisatin (green) with site-2, highlighting key residues and binding interactions. **c** Three-dimensional (3D) representation of pisatin (green) bound to position-2 of site-2 in the PI3K complex. **d** Two-dimensional (2D) interaction diagram of pisatin (green) with site-2, position-2, detailing specific residues and interaction types

Molecular docking analysis revealed that pisatin binds preferentially to two positions within site 2, with additional, although weaker, binding observed at the ATP-binding site, as indicated by the low binding energy values (Table S5). These findings suggest that pisatin would exert its activity through binding to PI3K, consistent with the network pharmacology in-silico prediction. This prediction could provide insights into its potential role in wound-healing mechanisms; however, further experimental investigation is needed to confirm these effects. Analysis of the pisatin-PI3K complex at the first position of site 2, at the interface of the two units of PI3K, identified several key interactions stabilizing the ligand within the binding pocket (Fig. 10a&b). Hydrogen bonds were observed with ASN (B:377) and ASN (A:575), while LYS (B:379) participated in a polar interaction, further stabilizing the ligand-protein complex. Hydrophobic interactions were also prominent, involving residues such as LEU (A:570) and TRP (A:574), which likely contribute to the ligand’s binding affinity. Additionally electrostatic interactions with HIS (A:362) and ASN (A:605) were observed, potentially enhancing the overall binding stability of pisatin.

At the second position within Site 2, pisatin occupied the interface between the p110α and p85α subunits, forming hydrogen bonds with ASN (A:467), ARG (B:481), and LYS (A:678). These interactions are likely critical for stabilizing the ligand within the enzyme’s active site. Furthermore, TYR (B:556) engaged in a π-π stacking interaction with the benzofuran moiety of pisatin, further reinforcing binding affinity. Significant hydrophobic interactions were also observed with ILE (B:477) and ALA (B:553), suggesting a strong contribution from van der Waals forces to the overall binding stability (Fig. 10c&d). 

## Conclusion

Bio-guided fractionation and isolation were employed to explore potential new applications of the aerial parts of *Pisum sativum* L., attributed to their rich content of bioactive compounds exhibiting immunomodulatory, anti-inflammatory, and wound-healing activities. Notably, the petroleum ether (PSP) and ethyl acetate (PSE) fractions exhibited significant immunomodulatory effects by enhancing PBMC viability and reducing inflammatory markers such as TNF-α, IL-6, and NO. Chromatographic and metabolomic analyses identified seven major compounds, among which pisatin (4), pisumflavonoside I (7), and quercetin-3-*O*-*β*-D-glucopyranoside (6) exhibited the most promising anti-inflammatory and immunomodulatory properties. Notably, compounds 4 and 7 also demonstrated the strongest wound-healing effects, highlighting the therapeutic potential of *P. sativum* aerial parts. Network pharmacology analysis suggested that pisatin enhances wound healing by modulating the PI3K pathway, which plays a key role in cell survival, proliferation, and tissue regeneration. Molecular docking studies further revealed strong binding interactions between pisatin and PI3K, suggesting its potential involvement in PI3K-related signaling and wound healing. These findings suggest *P. sativum* L. aerial parts as a promising source of bioactive compounds with potential wound-healing activity. Further in vivo studies and enzymatic assays should be conducted to validate the mechanism of action of pisatin (4) and assess its potential for clinical application.

## Supplementary Information

Supplementary Material 1.

## Data Availability

All data generated or analyzed in this study are fully presented in this article and its accompanying supplementary information.
