# Supplementary material for: Unveiling the wound-healing potential of Pisum sativum aerial biomass through integrated bioassay-guided, LC–MS/MS, and network pharmacology approaches
Source: BMC Complement Med Ther. 2026 Mar 23;26:130. doi: 10.1186/s12906-026-05311-8 (PMC13064283; doi:10.1186/s12906-026-05311-8)
Supplement: Supplementary file 1 — Supplementary Material 1. [file 12906_2026_5311_MOESM1_ESM.docx]

SUPPLEMENTARY INFORMATION

**Unveiling the Wound-Healing Potential of *Pisum sativum* Aerial Biomass through integrated Bioassay-Guided, LC–MS/MS, and Network Pharmacology Approaches**

Noha M. Kadash ^1^, [Abdullah A. Elgazar](https://www.tandfonline.com/author/Elgazar%2C+Abdullah+A) ^1^, Mona El-Aasr^2^, [Ramadan A. El-Domany](https://www.tandfonline.com/author/El-Domany%2C+Ramadan+A)^3^, Marwa Balaha^4,5,^^†^, Fardous F. El-Senduny^6,7^ ,Viviana di Giacomo^5,8^, Mai H. ElNaggar ^1,*^

^1^ Department of Pharmacognosy, Faculty of Pharmacy, Kafrelsheikh University, Kafrelsheikh, P.O. Box 33511, Egypt

^2^ Department of Pharmacognosy, Tanta University, College of Pharmacy, El-Guish Street, Tanta 31527, Egypt

^3^ Department of Microbiology and Immunology, Faculty of Pharmacy, Kafrelsheikh University, Kafrelsheikh, Egypt

^4^ Department of Pharmaceutical Chemistry, Faculty of Pharmacy, Kafrelsheikh University, Kafrelsheikh, P.O. Box 33516, Egypt

^5^ Department of Pharmacy, “G. d’Annunzio” University of Chieti-Pescara, Chieti, P.O. Box 66100, Italy

^6^ Biochemistry division, Chemistry Department, Faculty of Science, Mansoura University, Mansoura 35516, Egypt

^7^ Department of Pathology and Laboratory Medicine, University of Miami, Sylvester Comprehensive Cancer Center, Miami, FL 33136, USA

^8^ UdA Tech Lab, G. d’Annunzio University of Chieti-Pescara, Chieti, P.O. Box 66100, Italy

**^*^Corresponding author:** Mai H. ElNaggar

**Email address:** [mai_elnaggar@pharm.kfs.edu.eg](mailto:galal_magdy@pharm.kfs.edu.eg)

^†^**Co-corresponding author:** Marwa Balaha

**Email address:** marwa.balaha@unich.it

**1. Materials and Methods**

**1.1. General experimental procedures**

^1^H-NMR and APT spectra were recorded on BRUKER Ascend^TM^ 400 spectrometer or a JEOL ECA 500 NMR Spectrometer (500 MHz ^1^H and 126 MHz ^13^C NMR) using different deuterated solvents such as CDCl_3_ or CD_3_OD solvents and TMS as internal standard for chemical shifts. Chemical shifts (δ) were expressed in ppm with reference to the TMS resonance. ESI-MS data were determined using Advion compact mass spectrometer (NY, USA) or EI-MS using TSQ Quantum Access MAX Triple Quadrupole Mass Spectrometer (Thermo Fisher Scientific, USA). Normal phase chromatography was carried out using silica gel 60-230 mesh (Merck, Germany) packed by the wet method in the specific solvents. Vacuum liquid chromatography (VLC) was carried out using silica gel GF_254_ for TLC (SIGMA-ALDRICH) and packed using a dry packing method. The solvents used for extraction and chromatographic separation were purchased from Sigma-Aldrich (Missouri, USA). Analytical thin layer chromatography was performed on precoated silica gel 60 GF_254_ on aluminum sheets (Merck, Germany). Plates were developed in different solvent mixtures and the developed chromatograms were visualized under UV light 254 nm and 366 nm. Sephadex LH-20 (Sigma-Aldrich chemical Co.) and Reversed phase octadecylsilyl-silica gel (RP-C18, Merck, Germany) were used for purification. Visualization of the compounds was carried out using vanillin‐sulfuric acid spray reagent [1]. Gallic acid, Foline-Ciocalteu reagent, quercetin, aluminum chloride, sodium carbonate was purchased from Sigma-Aldrich, USA. HPLC methanol was purchased from Merck (Merck, Germany).

**1.2. Plant extraction and preparation of different fractions**

**1.2.1. Extraction of plant material**

Five kilograms of the aerial parts of *P. sativum* were shade-dried at room temperature for approximately two weeks. The dried plant material was then reduced to a fine powder. The powdered aerial parts (5 kg) were extracted by maceration in a glass jar with 95% methanol (4×6 L) at room temperature. The combined methanol extract was then concentrated at 40°C under reduced pressure using a rotary evaporator, producing 730 g of crude total methanolic extract.

**1.2.2. Fractionation of the total methanolic extract**

The total methanolic extract of the aerial parts of *P. sativum* (PST) was sequentially fractionated using several organic solvents (petroleum ether 60/80, methylene chloride, ethyl acetate, and *n*-butanol, respectively). Each resulting fraction was evaporated to dryness, yielding 100 g of petroleum ether fraction (PSP), 2.1 g of methylene chloride fraction (PSM), 11.5 g of ethyl acetate fraction (PSE), and 70 g of and *n*-butanol fraction (PSB).

Part of the petroleum ether fraction (6 g) was saponified under reflux using 10% alcoholic potassium hydroxide in order to prepare the saponifiable and unsaponifiable matter of the plant [2]. The unsaponifiable matter was extracted with diethyl ether and evaporated to dryness after the removal of excess alkali and kept for column chromatography. The saponifiable free fatty acids were liberated by acidification, then extracted with diethyl ether, dried in the vacuum oven, and weighed. Methylation of the obtained fatty acids was carried out with anhydrous methanol and concentrated sulfuric acid to be further used for Gas chromatography-mass spectrometry (GC-MS) analysis.

**1.3. Determination of the total flavonoid and total phenolic contents in different fractions of *P. sativum***

The total flavonoid content (TFC) was assessed using the aluminum chloride colorimetric assay as described by Ebrahimzadeh et al. [3] with slight modifications. Quercetin was used as a standard compound and prepared in different concentrations including 1000, 500, 250, 125, 62.5, 31.2, 15.6, and 7.8 μg/mL in 70% methanol to establish a calibration curve (**Figure S1a**). Briefly, 250 µL of the stock solution (1 mg/1 mL) of each fraction of *P. sativum* or the standard quercetin solutions was mixed with 750 µL of spectroscopic methanol followed by 50 µL of 10% aluminum chloride. 50 µL, 1 M potassium acetate and 1400 µL distilled water were added to the mixture. For each fraction, a blank experiment was conducted by substituting aluminum chloride with distillated water. 70% methanol was used as the blank. All reagents were mixed and incubated for 30 min at room temperature and protected from light. Each measurement was performed in experimental triplicate, and the assay was repeated on different days to ensure repeatability and reproducibility. The absorbance was measured at λ 415 nm with a microplate reader (BioTek Epoch 2, BioTek Instruments, Inc., Winooski, Vermont, USA). The total flavonoid contents were expressed as mg quercetin Equivalents (QE) per g of the plant extract.

The total phenolic content (TPC) was determined using the 96-well microplate Folin–Ciocalteu method [4] with some modifications. A total of 40 μL of each fraction (1 mg/1 mL) was mixed with 1800 μL of (10% v/v) Folin-Ciocalteu reagent. The mixture was left for 5 min. and then 1200 µL of (7.5% w/v) sodium carbonate solution was added. The mixture was kept for one hour in a dark area at room temperature. Each measurement was performed in experimental triplicate, and the assay was repeated on different days to ensure repeatability and reproducibility. The absorbance was measured at λ765 nm using the microplate reader (BioTek Epoch 2, BioTek Instruments, Inc., Winooski, Vermont, USA). 70% methanol was used as the blank while gallic acid was used as the standard phenolic compound. Serial dilutions of gallic acid (1000–7.8 μg/mL) were used for the establishment of the calibration curve (**Figure S1b**). Total phenolic contents were expressed as mg gallic acid equivalents (GAE) per g of the plant extract.


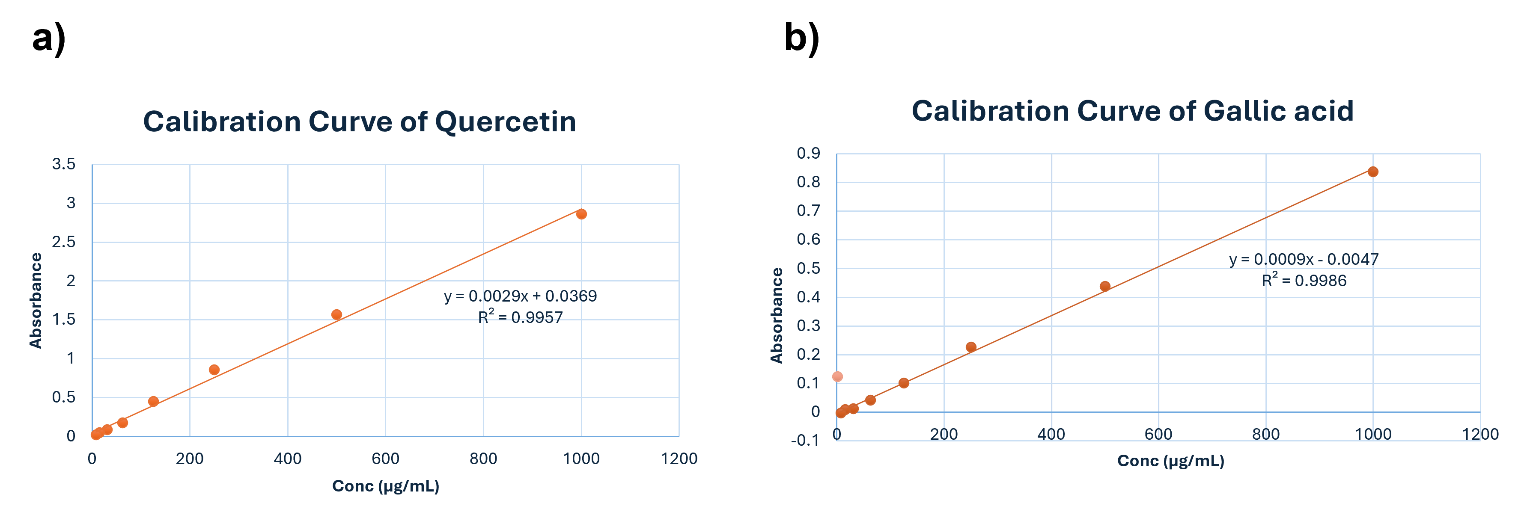


**Figure S1. a)** Calibration curve of quercetin at concentrations of 1000-7.8 µg/mL, **b)** Calibration curve of Gallic acid at concentrations of 1000-7.8 µg/mL.

**1.4. Evaluation of the biological activity of the obtained fractions**

*1.4.1. Isolation of PBMCs*

The Peripheral blood mononuclear cells (PBMCs) were isolated from a blood sample obtained from a participant at Kafr Elsheikh University Hospital. All procedures were conducted in accordance with the Declaration of Helsinki and complied with local Institutional Review Board (IRB) requirements. Ethical approval was granted under approval number KFSIRB200-893. The purpose and nature of the study were fully explained to the participant, and written informed consent was obtained prior to sample collection [5]. PBMCs were isolated according to the instructions from Eppendorf company by using Ficoll Plus 1.077 (Solarbio, Cat. No. P4350) [6, 7]. Cells were maintained in RPMI-1640 medium with L-glutamine (Biowest, L0500) supplemented with 10% fetal Bovine serum (Life Science Production, S-001-AU) and 1% penicillin-streptomycin solution in a culture plate suitable for the experiment at 37°C in a humidified atmosphere of 5% CO_2_, and 95% air for 24 hours.

*1.4.2. Immunomodulatory activity evaluation on PBMCs*

The immunomodulatory effects of PST, PSP, PSM, PSE, and PSB were initially evaluated on human peripheral blood mononuclear cells (PBMCs). PBMCs were seeded at (5 x 10^4^) cells/mL in 96-well plates and treated with serial dilutions of each extract (100, 50, 25, 12.5, 6.25, and 3.125 µg/mL), dissolved in DMSO, with a final solvent concentration not exceeding 0.01% (v/v). After 24 hours of incubation at 37°C in a 5% CO₂ atmosphere, cell viability was evaluated using the WST-1 reagent, water-soluble tetrazolium salt-1 (Roche, CELLPRO-RO, Cat. No. 5015944001) as described by Ishiyama et al. [8]. After 1 hour of incubation with WST-1, 10 µL of 1% sodium dodecyl sulfate (SDS) was added to terminate the reaction, and the absorbance was measured at λ420 nm. Morphological changes were observed using an Optika inverted microscope.

*1.4.3. Cytokine and nitric oxide analysis in RAW 264.7 cells*

RAW 264.7 murine macrophages (obtained from Nawah scientific, Cairo, Egypt) were seeded at a density of 5 x 10^5 cells per well in 6-well plates and incubated overnight to allow for cell adherence. The cells were then pretreated for 3 hours with either PSP or PSE fractions (25 µg/mL) or 1 µM of dexamethasone, dissolved in DMSO (final concentration ≤ 0.01% v/v), followed by stimulation with 5 µg/mL of lipopolysaccharide (LPS) for an additional 24 hours. This pretreatment allowed for the assessment of the fractions' ability to modulate the expression of the inflammatory cytokine, TNF-α.

For gene expression analysis, total RNA was isolated from the cells using TRIzol reagent (Thermo Fisher Scientific), following the manufacturer's protocol. The concentration and purity of RNA were evaluated using Thermo Scientific™ NanoDrop™ One Microvolume UV-Vis Spectrophotometer. 4 µg of RNA was used for cDNA synthesis using a TopScript™ RT DryMix (dN18/dN6 plus, Cat. No. RT220, Enzynomics) kit. The relative expression of TNF-α was measured using quantitative PCR (qPCR) with the SensiFAST™ SYBR® Hi-ROX Kit on a StepOne™ Real-Time PCR System (Applied Biosystems™). TNF-α expression levels were normalized to the housekeeping gene β-actin and calculated using the 2^-ΔΔCT^ method. The primer for TNF-α and β-actin were synthesized by Eurofins Scientific and listed in **Table S1**.

**Table S1.** Primer sequence and their reference in NCBI

| **Gene** | **Primer Sequence** | **Reference Sequence** |
| --- | --- | --- |
| *TNF-α* | **F**: 5'-GCC TCT TCT CAT TCC TGC TTG-3' | [NM_001278601.1](https://www.ncbi.nlm.nih.gov/entrez/viewer.fcgi?db=nucleotide&id=518831588) |
|  | **R**: 5'-CTG ATG AGA GGG AGG CCA TT-3' |  |
| *β-Actin* | **F**:5-GGG AAA TCG TGC GTG ACA T-3 | [NM_007393.5](https://www.ncbi.nlm.nih.gov/entrez/viewer.fcgi?db=nucleotide&id=930945786) |
|  | **R**:5-GCG GCA GTG GCC ATC TC-3 |  |

After confirming the ability of the PSP and PSE fractions to modulate TNF-α gene expression, cell culture supernatants were collected for further cytokine and nitric oxide (NO) analysis. IL-6 levels were quantified using a 100 µL aliquot of the supernatant and an enzyme-linked immunosorbent assay (ELISA) kit (FineTest® Company, Cat. No. EM0121), following the manufacturer's instructions. Nitric oxide (NO) levels were measured using the Griess reagent assay [9], where 100 µL of the supernatant was mixed with 100 µL of Griess reagent, incubated for 30 minutes at room temperature in the dark. The absorbance was then measured at 490 nm and plotted against concentration to establish a calibration curve (Figure S2).


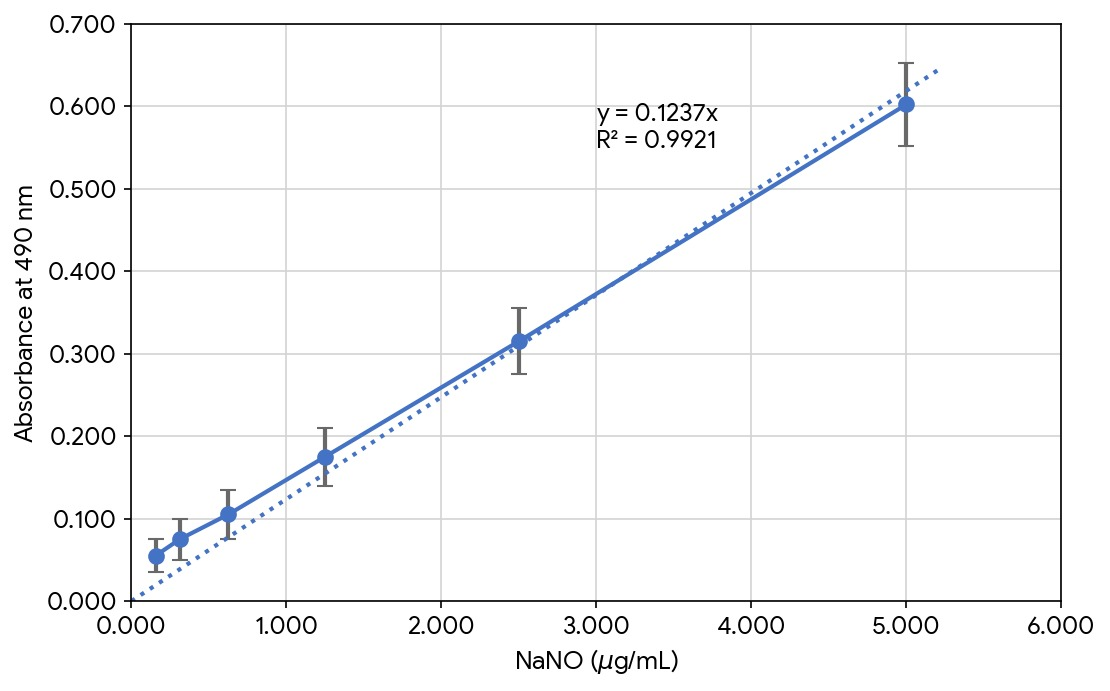


**Figure S2.** Standard curve for nitric oxide quantification. Mean values (n = 3). Sample concentrations were determined by interpolation from the standard curve.

**1.5. Chemical investigation and isolation of major compounds from the bioactive fractions of *Pisum sativum* L.**

*1.5.1. GC-MS analysis of the saponifiable matter in the petroleum ether fraction of* *P. sativum L. (PSP).*

The GC-MS analysis was conducted using a Thermo Scientific, Trace GC Ultra/ISQ Single Quadrupole MS system equipped with a TG-5MS fused silica capillary column (30 m, 0.251 mm, 0.1 mm film thickness). For GC-MS detection, an electron ionization was employed with an ionization energy of 70 eV, and Helium served as the carrier gas at a constant flow rate of 1 mL/min. The injector and mass spectrometer transfer line temperatures were both maintained at 280°C. The oven temperature was initially set to 150°C and maintained for 4 min., then increased at a rate of 5°C per min. until reaching 280°C, where it was held for an additional 4 min. The quantification of all identified components was performed using a percentage relative peak area. Tentative identification of the compounds was achieved by comparing their relative retention times and mass spectra with entries in the NIST and WILEY mass spectral libraries.

*1.5.2. LC-MS/MS analysis for metabolite profiling of the ethyl acetate fraction of P. sativum L. (PSE)*

A weighed portion of PSE (50 mg) was dissolved in 1 mL of a solvent mixture comprising deionized water, methanol, and acetonitrile in a 50:25:25 ratio. The solution was vortexed for 2 minutes, subjected to ultrasonic treatment for 10 minutes, and then centrifuged at 1000 rpm for 10 minutes. The solution was diluted with the same solvent mixture, and 10 µL of the sample, at a concentration of 2.5 µg/µL, were used for injection. Liquid chromatography-electrospray ionization-tandem mass spectrometry (LC-ESI-MS/MS) analysis was conducted using negative ion mode at the Children’s Cancer Hospital’s Proteomics and Metabolomics Unit (57357) following the method adopted in research studies [10, 11].

*1.5.3. Compounds isolation and purification*

The unsaponified petroleum ether fraction (1.3 g) was subjected to silica gel column chromatography (CC), (*ϕ* 2×100 cm, 39 g), and eluted with a gradient mixture of *n-*hexane- ethyl acetate (EtOAc). The eluted fractions were concentrated, screened by TLC and similar fractions were collected based on their composition to provide ten groups (G1-G10). Group G4 (44-48, 40 mg) eluted with *n*-hexane-EtOAc (97:3 v/v) was purified by successive washing with *n*-hexane to obtain compound **1**, 25 mg. Group G6 (72-77, 50 mg) eluted with *n*-hexane-EtOAc (95:5, v/v) was also purified by successive washing with n-hexane to afford compound **2**, 30 mg. Group G8 (128-136, 63 mg) eluted with *n*-hexane-EtOAc (87:13, v/v) was re-chromatographed over a normal silica gel CC (*ϕ* 1×70 cm, 7.6 g silica) using a gradient mixture of *n*-hexane-CH_2_Cl_2_ to obtain compound **3,** 30 mg.

50 g of the petroleum ether fraction of *P. sativum* (PSP) were subjected to VLC (*ϕ* 7×20 cm) containing 150 g of silica gel and packed in petroleum ether (PE) (100%), adopting a gradient elution method with CH_2_Cl_2_ (from 0:100%), followed by EtOAc (from 0:100%). Fractions of 200 mL were collected, concentrated, and screened by TLC. Similar fractions were pooled together to provide 10 groups (P1-P10). Group P4 (3.2 g, eluted with PE-CH_2_Cl_2_ (60:40, v/v), was further subjected to VLC (*ϕ* 7×20 cm, 150 g silica) using gradient elution with PE-CH_2_Cl_2_ solvent system resulting in sex fractions (P4-1: P4-6). Fraction P4-4, 0.5 g, eluted with PE-CH_2_Cl_2_ (40:60, v/v) was re-chromatographed on silica gel using VLC (*ϕ* 1.5×6 cm, 15 g) and gradient elution with PE- CH_2_Cl_2_ (from 0:100%). The effluents (70 mL each) were evaporated and grouped based on their TLC screening. A major compound containing fraction (130 mg) eluted with PE-CH_2_Cl_2_ (40:60 v/v) was further chromatographed on a normal silica gel CC, (*ϕ* 1×70 cm, 6.5 g), using gradient elution with PE-EtOAc to afford compound **4** (50 mg).

The ethyl acetate fraction (PSE, 11 g) was chromatographed over silica gel VLC (*ϕ* 7×20 cm, 150 g), packed in CH_2_Cl_2_ (100%), and eluted by gradient elution of EtOAc (from 0:100%), followed by methanol (MeOH) (from 0:50%). The effluents (200 mL each) were evaporated and grouped based on their composition to yield 12 groups (E1: E12). Fraction E2 (1.7 g), eluted with CH_2_Cl_2_- EtOAc (85:15, v/v) was subjected to normal silica gel CC (*ϕ* 2×70 cm, 50 g silica), packed in CH_2_Cl_2_ (100%), and eluted with gradient elution of EtOAc (from 0:20%). Similar fractions were combined based on TLC screening to afford 5 sub-fractions (E2-1: E2-5). Sub-fraction E2-2, 140 mg, eluted with CH_2_Cl_2_- EtOAc (90:10, v/v) was further purified on reversed phase medium pressure CC (*ϕ* 1×25 cm, 20 g) using gradient elution of H_2_O-MeOH to afford compound **5** (30 mg) eluted with H_2_O-MeOH (88:12 v/v). Fraction E7 (2.7 g eluted with 7% methanol in ethyl acetate) was applied to isocratic CC with 100% EtOAc to give a yellow residue that was further purified on Sephadex LH-20 with 100% MeOH as a mobile phase to give compound **6** (15 mg). Compound **7** (30 mg) was obtained by purifying fraction E10 (427 mg, eluted with 20% methanol in ethyl acetate) on Sephadex LH-20 with MeOH.

**1.6. *In-silico* prediction of pisatin molecular targets and molecular docking analysis**

*1.6.1. Molecular target prediction and Network pharmacology*

To predict the potential targets of pisatin, we utilized the PharmMapper server (<http://www.lilab-ecust.cn/pharmmapper/>) and the SwissTargetPrediction server (<http://www.swisstargetprediction.ch/>). After consolidating the predicted targets, duplicate entries were removed, and the final target list was saved as a CSV file. Additionally, a dataset of molecular targets associated with wound healing, was retrieved from GeneCards (http://www.genecards.org). Using the Venny 2.1.0 tool (https://bioinfogp.cnb.csic.es/tools/venny/), we identified the overlapping targets between pisatin’s predicted targets and the wound-healing dataset.

Following target identification, an enrichment analysis was conducted using Input 2.0 server (<https://cbcb.cdutcm.edu.cn/INPUT>) to elucidate their functional significance. Gene ontology (GO) analysis was performed to categorize the targets into biological processes (BP), molecular functions (MF), and cellular components (CC). Furthermore, the Kyoto Encyclopedia of Genes and Genomes (KEGG) pathway analysis was carried out to map the relevant signaling pathways. A protein–protein interaction (PPI) network was then constructed by applying a confidence score threshold of 0.7 to ensure the reliability of interactions. Gene distribution analysis was conducted to identify key targets that show significant correlations with the biological activity of pisatin.

*1.6.2. Molecular Docking analysis*

PI3K was identified as the primary target for pisatin based on systems pharmacology analysis. Consequently, its binding mode was explored using molecular docking. The PDB file of PI3K (3HHM) and the 3D chemical structure of pisatin were submitted to the protein and ligand optimization panels integrated within the CB-Dock2 online server (<https://cadd.labshare.cn/cb-dock2>). A blind docking approach was employed to examine the interaction of pisatin with the identified active sites. Subsequently, docking poses were ranked according to their scores. Finally, the best docking pose was visualized using the Discovery Studio Visualizer to analyze its interaction with the binding site [12].

**2. Results and Discussion**

**2.1. Determination of** **the total flavonoid and total phenolic contents in** **different fractions of *P. sativum***

**Table S2.** Total phenolic content (TPC) and total flavonoid content (TFC) of the total extract and different fractions of P. sativum aerial parts at a concentration (1 mg/mL).

| **Extract** | **TFC** **(mg QE/ g)** | **Average absorbance at 415 nm** | **TPC** (**mg GAE/ g)** | **Average absorbance at 765 nm** |
| --- | --- | --- | --- | --- |
| **PST** | 27.10 ± 0.73 | 0.1155 ± 0.0021 | 44.11 ± 3.14 | 0.035 ± 0.0028 |
| **PSP** | 38.48 ± 0.24 | 0.1485 ± 0.0007 | 25.78 ± 2.36 | 0.018 ± 0.0021 |
| **PSM** | 22.28 ± 1.22 | 0.1015 ± 0.0035 | 167.44 ± 9.43 | 0.146 ± 0.0084 |
| **PSE** | 52.62 ± 1.71 | 0.1895 ± 0.0049 | 149 ± 9.43 | 0.13 ± 0.0084 |
| **PSB** | 55.38 ± 0.24 | 0.1975 ± 0.0007 | 129.11 ± 0.79 | 0.1115 ± 0.0007 |

Results are expressed as mean ± SD. PST: The total methanolic extract, PSP: petroleum ether fraction, PSM: methylene chloride fraction, PSE: ethyl acetate fraction, PSB: n-butanol fraction

**2.2. GC-MS analysis of the saponifiable matter in the petroleum ether fraction of *P. sativum* L.**


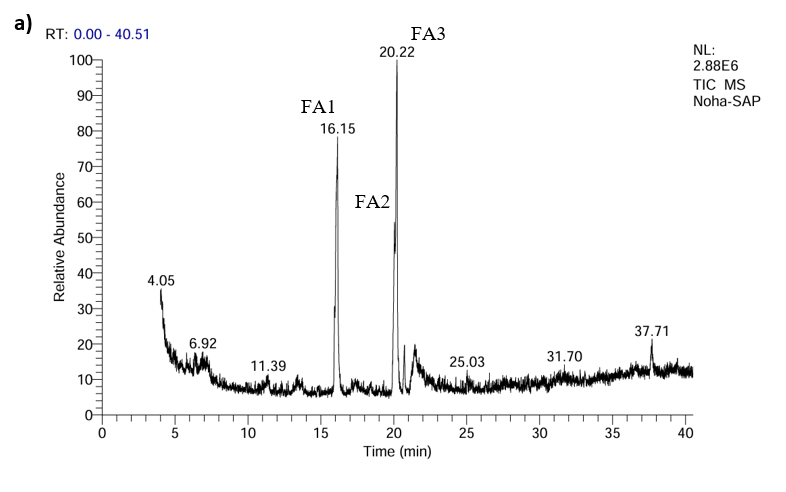


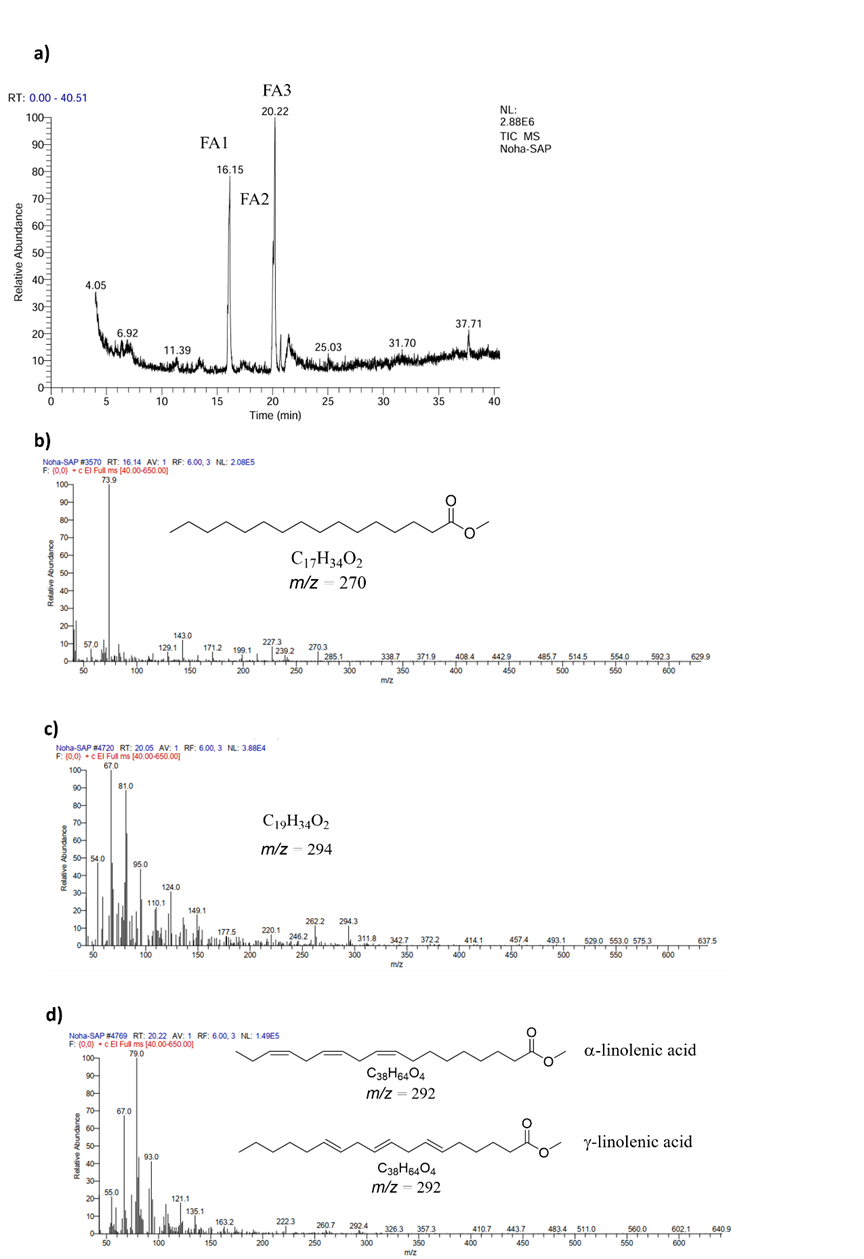


**Figure S3. a)** Gas Chromatography-Mass Spectrometry total ion chromatogram of fatty acid methyl esters tentatively identified in the aerial parts of *P. sativum* L, **b)** GC/EI-MS fragmentation spectrum of palmitic acid (**FA1**), **c)** GC/EI-MS fragmentation spectrum of Linoleic acid (**FA2**), **d)** GC/EI-MS fragmentation spectrum of linolenic acid (**FA3**).

**Table S3.** Results of GC-MS analysis of fatty acid methyl esters of *P. sativum* L.

| **Peak No.** | **R_t_ (min)** | **Name** | **[M]^+^**  ***m/z*** | **Molecular formula** | **Area (%)** | **References** |
| --- | --- | --- | --- | --- | --- | --- |
| FA1 | 16.15 | Hexadecanoic acid (palmitic acid), methyl ester | 270 | C_17_H_34_O_2_ | 22.26 | [13, 14] |
| FA2 | 20.05 | Linoleic acid methyl ester | 294 | C_19_H_34_O_2_ | 11.89 | [14, 15] |
| FA3 | 20.22 | Linolenic acid methyl ester that could be:   - α-Linolenic acid (9,12,15-octadecatrienoic acid) - γ-Linolenic acid (6,9,12-Octadecatrienoic acid) | 292 | C_19_H_32_O_2_ | 29.58 | [14, 16] |
| Total percent of identified fatty acids | | | | | 63.73% |  |
| Unsaturated fatty acids percent of identified fatty acids | | | | | 41.47% |  |
| Saturated fatty acids percent of identified fatty acids | | | | | 22.26% |  |

**2.3. LC-ESI-MS/MS Analysis of the ethyl acetate fraction of *P. sativum* L. (PSE)**

**Table S4.** Metabolic profiling of the ethyl acetate fraction of *P. sativum* (PSE) by LC-MS/MS analysis in negative ion mode.

| **peak** | **Identified Metabolite** | **RT (min)** | ***m/z***  **exp.**  **[M-H] ^-^** | ***m/z***  **theor.**  **[M-H] ^-^** | **Error**  **(ppm)** | **Molecular Formula** | **MS^2^ Fragments** | **Ref** |
| --- | --- | --- | --- | --- | --- | --- | --- | --- |
| **I**- **Flavonols and flavonol glycosides** | | | | | | | | |
| 1 | Isorhamnetin (3'-methoxy-4',5,7-trihydroxyflavonol) | 7.91 | 315.0518 | 315.051 | 2.53 | C_16_H_12_O_7_ | 300.0505, 297.0412, 269.0457, 271.0612, 246.0078, 151.0402 | [17-20] |
| 2 | Isorhamnetin-3-*O*-glucoside | 7.54 | 477.1034 | 477.1038 | -0.83 | C_22_H_22_O_12_ | 314.0386, 285.0399, 269.0366, 271.0068, 243.0358 | [19, 21] |
| 3 | Isorhamnetin-3-*O*-rutinoside | 7.19 | 623.1613 | 623.1617 | -0.64 | C_28_H_32_O_16_ | 315.0484, 300.0046, 299.0123 | [22, 23] |
| 4 | Kaempferol-7-*O*-neohesperidoside | 7.09 | 593.1507 | 593.1511 | -0.67 | C_27_H_30_O_15_ | 285.0412, 284.0336 | [24, 25] |
| 5 | Quercetin | 11.04 | 301.0349 | 301.0353 | -1.32 | C_15_H_10_O_7_ | 273.0490, 193.0138, 178.9997, 151.0036 | [26, 27] |
| 6 | Quercetin-3-*O*-arabinoside | 7.42 | 433.0770 | 433.0776 | -1.38 | C_20_H_18_O_11_ | 387.1949, 300.0189, 271.0556, 243.0644, 161.0263 | [22, 28] |
| 7 | Quercetin-3-*O*-β-D-glucopyranoside (Isoquercitrin) | 6.78 | 463.0887 | 463.0882 | 1.07 | C_21_H_20_O_12_ | 301.0328, 300.0290, 271.0165 | [29-32] |
| 8 | Quercetin-3,4'-*O*-di-β-glucopyranoside | 5.77 | 625.1418 | 625.141 | 1.27 | C_27_H_30_O_17_ | 301.0329 | [33] |
| 9 | Quercetin 3-*O*-(6″″-*O*-*cis*-*p*-coumaroyl) sophorotrioside (Pisumflavonoside I) | 6.91 | 933.2325 | 933.2313 | 1.28 | C_42_H_46_O_24_ | 787.2043, 625.1480, 463.0834, 300.0314 | [34] |
| 10 | 3,5,7-trihydroxy-4'-methoxyflavone  (Kaempferide) | 10.93 | 299.0563 | 299.0561 | 0.66 | C_16_H_12_O_6_ | 284.0354, 269.0466, 240.0428 | [23, 35] |
| **II- Flavones and flavone glycosides** | | | | | | | | |
| 11 | Acacetin-7-*O*-rutinoside  (Linarin) | 9.31 | 591.1709 | 591.1719 | -1.69 | C_28_H_32_O_14_ | 283.0626, 240.0422 | [36, 37] |
| 12 | Apigenin | 10.68 | 269.0449 | 269.0455 | -2.23 | C_15_H_10_O_5_ | 254.0564, 225.0579, 151.0037, 117.0346 | [17, 38] |
| 13 | Apigenin-7-*O*-glucoside | 8.05 | 431.0983 | 431.0983 | 0 | C_21_H_20_O_10_ | 311.0558, 269.0461, 268.0347, 211.0437, 150.9999 | [39-41] |
| 14 | Apigenin 7-*O*-neohesperidoside (Rhoifolin) | 9.16 | 577.1548 | 577.1562 | -2.42 | C_27_H_30_O_14_ | 269.0467, 268.0395, 271.0582 | [31, 42] |
| 15 | Luteolin | 7.95 | 285.0412 | 285.0404 | 3.12 | C_15_H_10_O_6_ | 270.0387, 133.0297 | [43] |
| 16 | Luteolin-7-*O*-glucoside | 7.4 | 447.0931 | 447.0936 | -1.11 | C_21_H_20_O_11_ | 285.0411, 284.0322 | [43] |
| **III-Flavanones and Flavanone Glycosides** | | | | | | | | |
| 17 | Eriodictyol-7-*O*-glucoside | 6.69 | 449.1077 | 449.1089 | -2.67 | C_21_H_22_O_11_ | 287.0922, 269.023, 151.0367, 135.0426 | [44, 45] |
| 18 | Isosakuranetin-7-*O*-neohesperidoside | 8.01 | 593.187 | 593.1875 | -0.84 | C_28_H_34_O_14_ | 285.0420, 270.0547 | [46, 47] |
| 19 | Naringenin | 10.29  10.97 | 271.0618 | 271.0611 | 2.58 | C_15_H_12_O_5_ | 165.0185, 151.0006, 119.0499, 108.0194 | [22, 25, 48] |
| 20 | Naringenin-7-*O*-glucoside  (Prunin) | 8.12 | 433.1183 | 433.114 | 9.92 | C_21_H_22_O_10_ | 271.0606, 151.0018, 119.0549 | [49] |
| **IV- Isoflavones** | | | | | | | | |
| 21 | Daidzein | 9.29 | 253.0505 | 253.0506 | -0.39 | C_15_H_10_O_4_ | 225.0548, 209.0594, 133.0305, 135.0056, 117.0363 | [50, 51] |
| 22 | Daidzein-8-*C*-glucoside  (Puerarin) | 6.64 | 415.1046 | 415.1034 | 2.89 | C_21_H_20_O_9_ | 392.0959, 369.2039, 253.0525, 179.0529 | [22, 52, 53] |
| 23 | Formononetin | 12.29 | 267.0663 | 267.0662 | 0.37 | C_16_H_12_O_4_ | 252.0439, 223.0386, 195.0450, 135.0104 | [51, 54, 55] |
| 24 | Glycitein  (Biochanin A) | 9.79 | 283.0611 | 283.0612 | -0.35 | C_16_H_12_O_5_ | 268.0325, 211.0355, 198.8978, 92.9272 | [51, 56] |
| **V- Anthocyanidin-3-*O*-glycoside** | | | | | | | | |
| 25 | Cyanidin-3-*O*-glucoside (Chrysanthemin) | 7.4 | 447.0931^*^ | 447.0927^*^ | 0.89 | C_21_H_21_O_11_^+^ | 285.0411, 284.0329, 255.0295, 227.0343 | [57, 58] |
| 26 | Cyanidin-3,5-di-*O*-glucoside (Cyanin) | 6.21 | 609.1455^*^ | 609.1455^*^ | 0 | C_27_H_31_O_16_^+^ | 447.0914, 285.0473, 284.0301, 227.0401, 255.0253 | [57, 59] |
| 27 | Malvidin-3-*O*-glucoside | 5.8 | 491.12^*^ | 491.1189^*^ | 2.23 | C_23_H_25_O_12_^+^ | 329.0676, 314.0430, 299.0204 | [60, 61] |
| 28 | Peonidin | 10.93 | 299.0563^*^ | 299.0555^*^ | 2.67 | C_16_H_13_O_6_^+^ | 284.0342, 256.0421, 227.0364 | [61, 62] |
| 29 | Peonidin-3-*O*-glucoside | 5.78 | 461.1086^*^ | 461.1084^*^ | 0.43 | C_22_H_23_O_11_^+^ | 415.1599, 299.0558, 284.0321, 256.0407, 227.0378 | [61, 63] |
| **VI- Flavanols** | | | | | | | | |
| 30 | Catechin or Epicatechin [64] | 7.4 | 289.0729 | 289.0717 | 4.15 | C_15_H_14_O_6_ | 245.0868, 204.912, 137.0263, 125.0509, 109.0331 | [38, 65-67] |
| **VII- Phenols and phenolic acids** | | | | | | | | |
| 31 | 1,2-Benzenediol (catechol) | 1.21 | 109.029 | 109.0295 | -4.58 | C_6_H_6_O_2_ | 108.0293, 91.0208, 81.0385 | [68] |
| 32 | Caffeic acid | 1.35 | 179.0348 | 179.0349 | -0.55 | C_9_H_8_O_4_ | 135.0461, 134.0394, 89.0423, 71.0140 | [31, 69, 70] |
| 33 | *p*-coumaric acid | 1.39 | 163.0406 | 163.0401 | 3.06 | C_9_H_8_O_3_ | 145.0302, 119.0497, 93.0344, 91.0567, 65.0399 | [38, 70, 71] |
| 34 | Ferulic acid | 1.42 | 193.0502 | 193.0506 | -2.07 | C_10_H_10_O_4_ | 178.0279, 149.0611, 134.0376, 117.0338, 106.0413 | [72, 73] |
| 35 | *p*-Hydroxy benzoic acid | 6.56 | 137.023 | 137.024 | -0.5 | C_7_H_6_O_3_ | 93.0322, 65.0393 | [38, 74, 75] |
| 36 | *p*-Hydroxyphenyl acetic acid | 4.51 | 151.04 | 151.04 | 0 | C_8_H_8_O_3_ | 135.0816, 107.0501, 59.0133 | [76] |
| 37 | Protocatechuic acid | 1.2 | 153.0196 | 153.0193 | 1.96 | C_7_H_6_O_4_ | 109.0306, 108.0233 | [71, 77, 78] |
| 38 | Phloridzin | 6.24 | 435.1294 | 435.1296 | -0.45 | C_21_H_24_O_10_ | 273.0758, 167.0348, 149.0239, 148.0144, 109.0294 | [43, 71] |
| 39 | Rosmarinic acid | 4.9 | 359.0779 | 359.0772 | 1.94 | C_18_H_16_O_8_ | 197.0472, 179.0360, 161.0253, 135.0453 | [38, 71] |
| **VIII- Aliphatic acids** | | | | | | | | |
| 40 | Azelaic acid | 1.24 | 187.0979 | 187.0976 | 1.60 | C_9_H_16_O_4_ | 169.0874, 143.1091, 125.0972, 97.0664 | [79-81] |
| 41 | 3-Hydroxy-3-methylglutaric acid | 1.32 | 161.0469 | 161.0455 | 8.69 | C_6_H_10_O_5_ | 117.0527, 115.0392, 103.0352, 99.0458, 59.0127 | [23, 82] |
| 42 | D- (+)-Malic acid | 1.08 | 133.0143 | 133.0142 | 0.75 | C_4_H_6_O_5_ | 115.0016, 87.0079, 72.7740 | [23, 43, 83] |

*: [M-2H]^-^


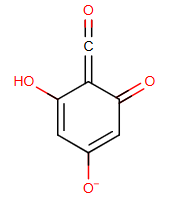

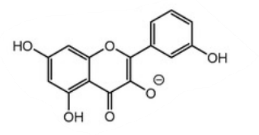

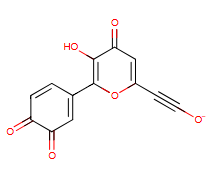

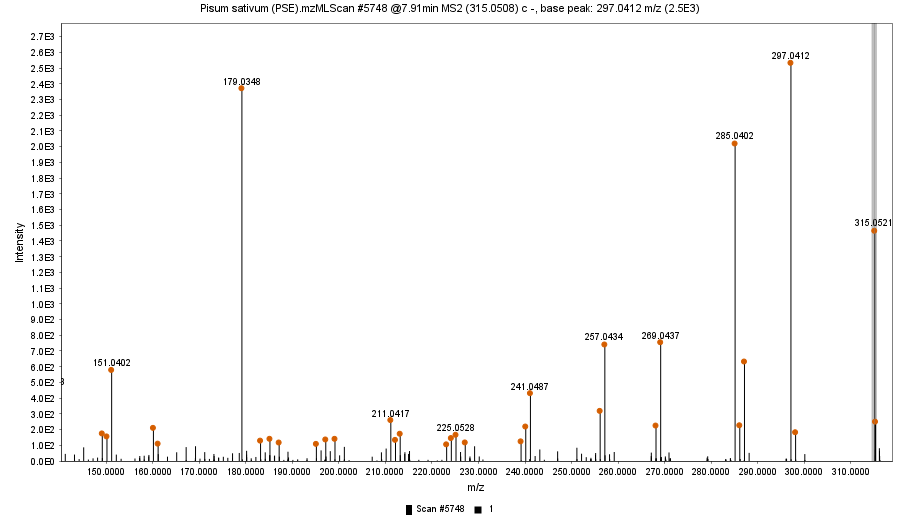


**151.0402**

**[M–CH_3_]^-^**

**300.024**

**[M–H_2_O]^-^**

**285.0402**

**257.0434**

**Figure S4.** ESI-MS/MS spectrum of isorhamnetin **(1)**.


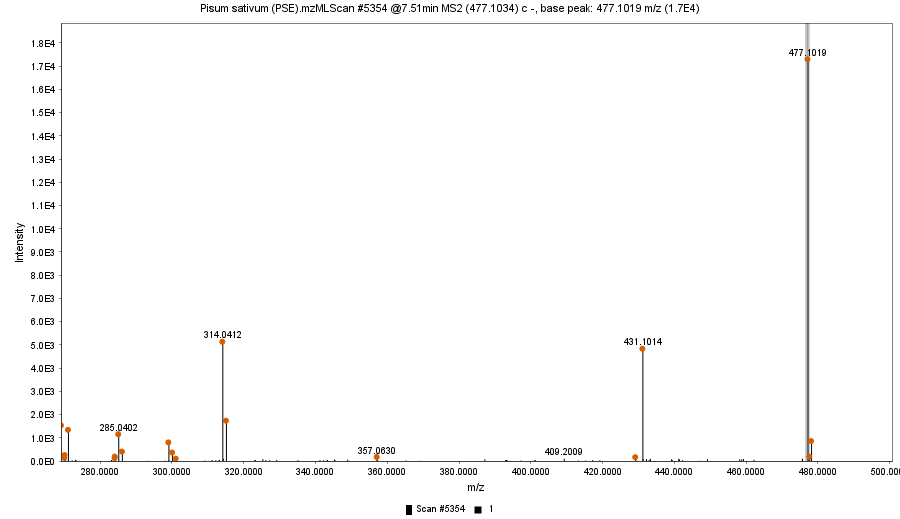

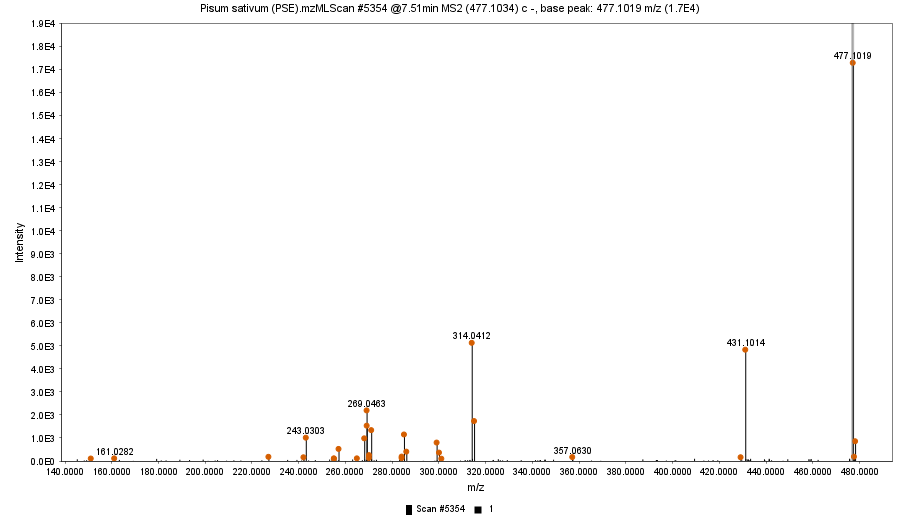


**315.048**0

**285.0402**

**357.0630**

**Figure S5.** ESI-MS/MS spectrum of isorhamnetin-3-*O*-glucoside **(2)**.


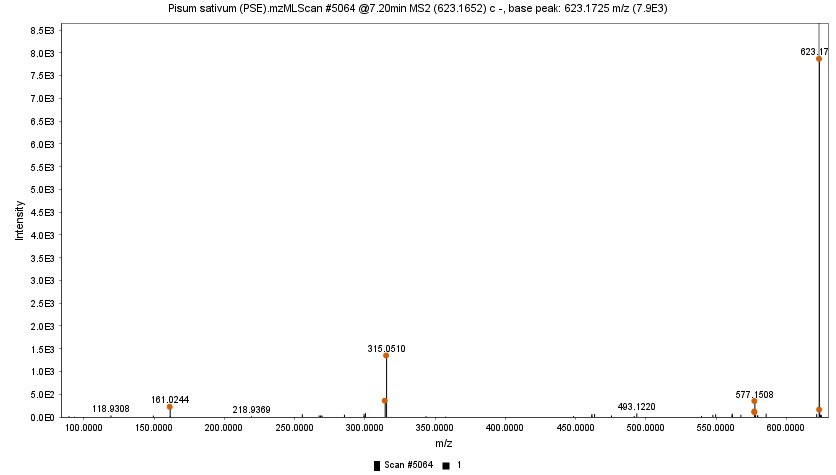


**Figure S6.** ESI-MS/MS spectrum of isorhamnetin-3-*O*- rutinoside **(3)**.


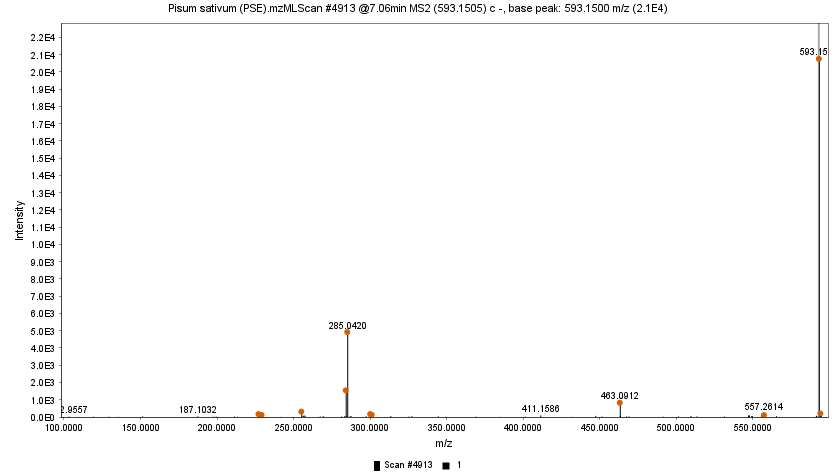


**Figure S7.** ESI-MS/MS spectrum of Kaempferol-7-*O*-neohesperidoside **(4)**.


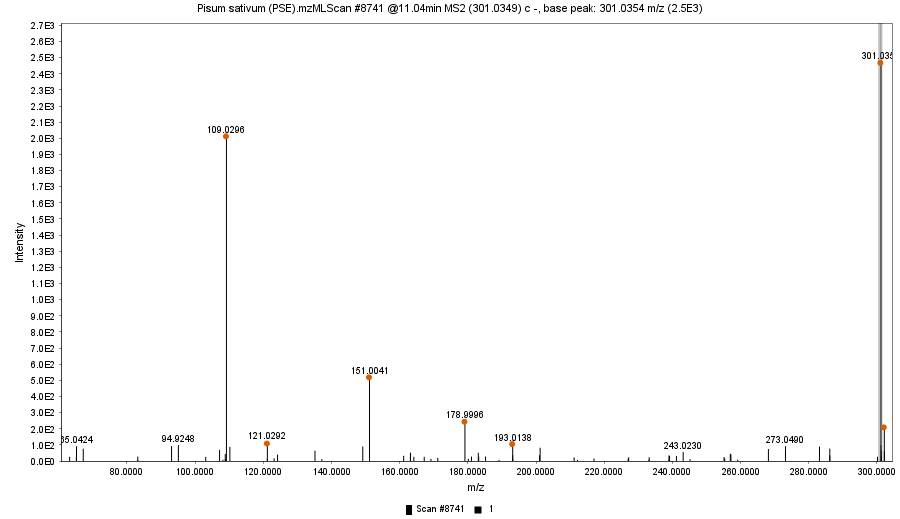


**Figure S8.** ESI-MS/MS spectrum of quercetin **(5)**.


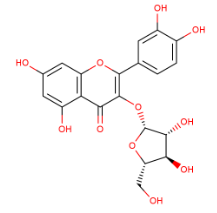

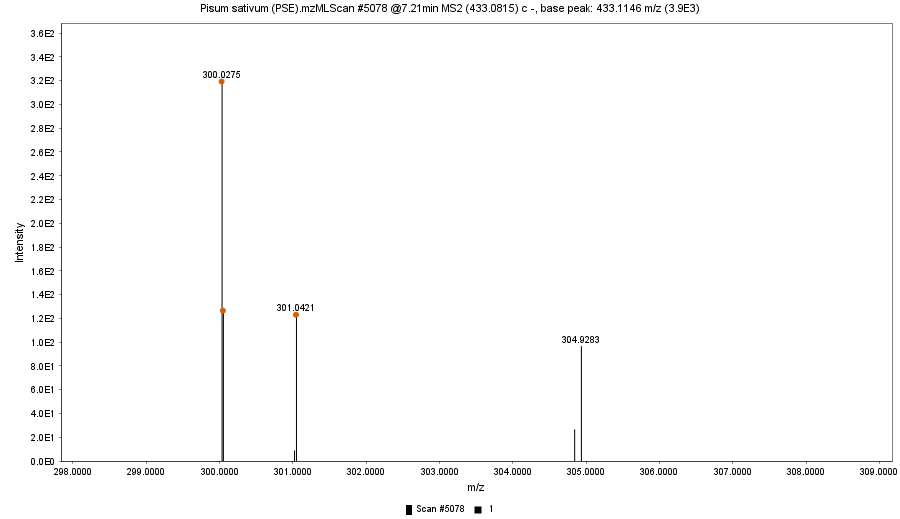

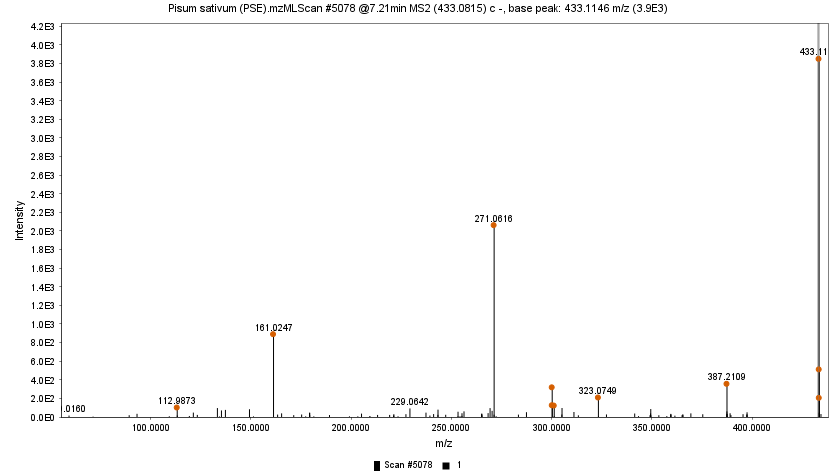


**387.2109**

**Figure S9.** ESI-MS/MS spectrum of quercetin-3-*O*-arabinoside **(6)**.


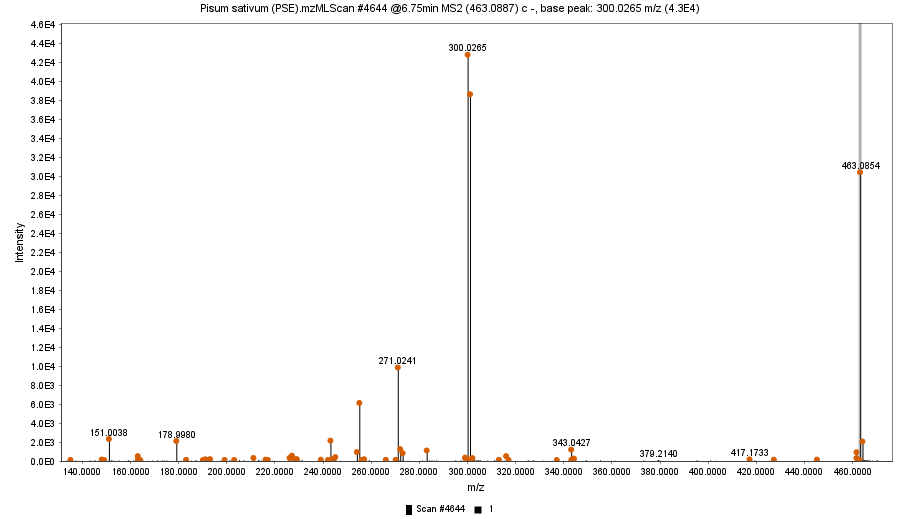


**Figure S10.** ESI-MS/MS spectrum of quercetin-3-*O*-β-D-glucopyranoside **(7)**.


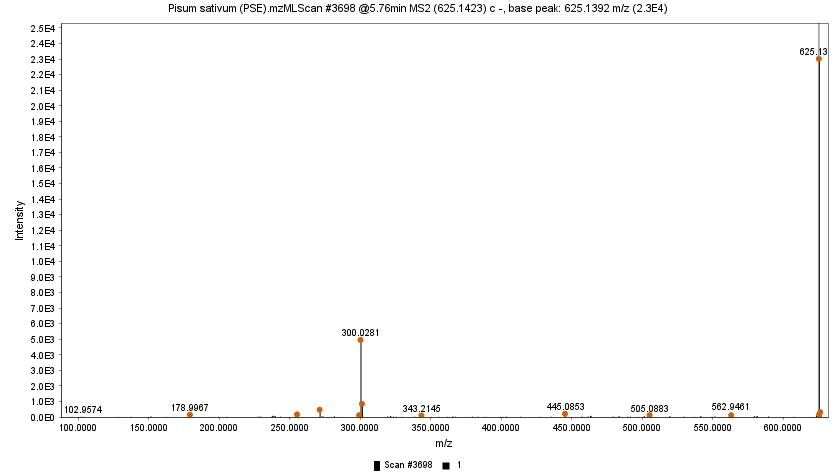


**Figure S11.** ESI-MS/MS spectrum of quercetin-3,4'-*O*-di-β-glucopyranoside **(8)**.


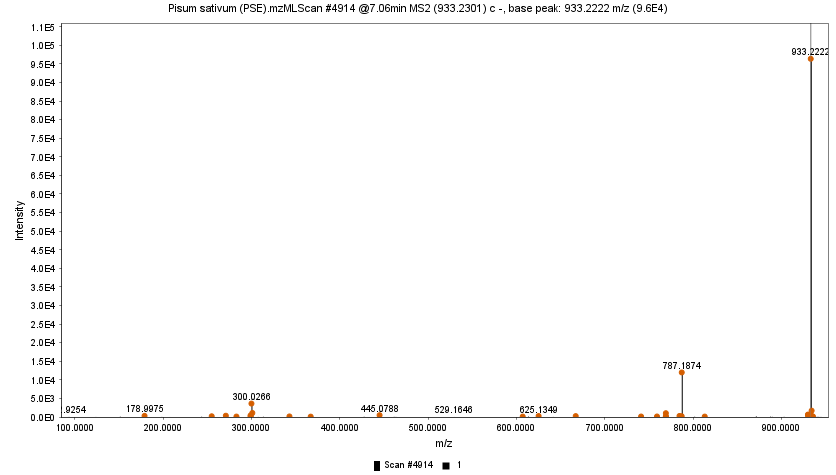


**Figure S12.** ESI-MS/MS spectrum of quercetin 3-*O*-(6″″-*O*-*cis*-*p*-coumaroyl) sophorotrioside **(9)**.

**
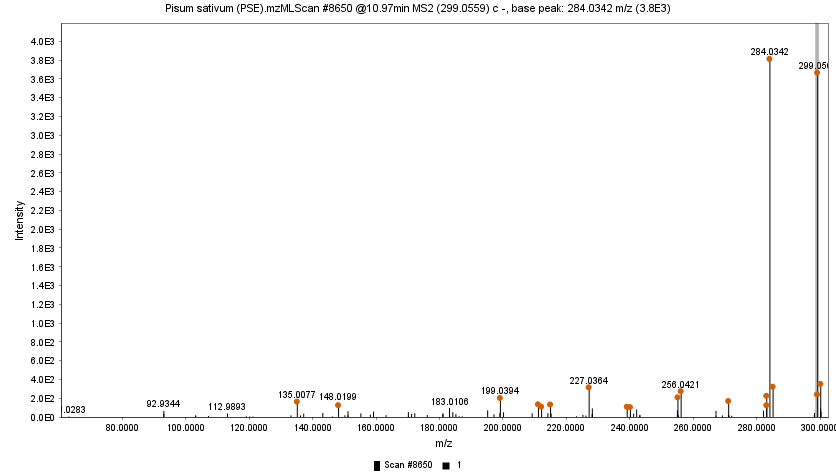
**

**Figure S13.** ESI-MS/MS spectrum of 3,5,7-trihydroxy-4'-methoxyflavone **(10)**.


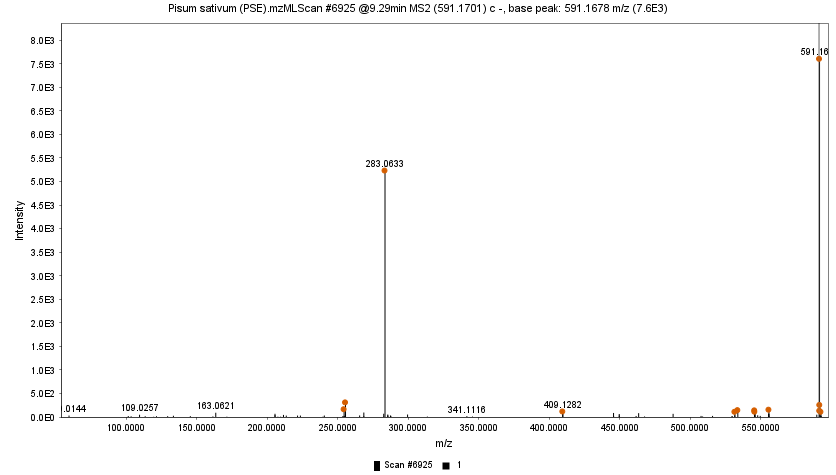


**Figure S14.** ESI-MS/MS spectrum of acacetin-7-*O*-rutinoside **(11)**.


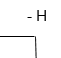

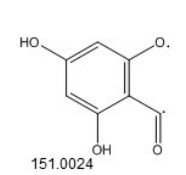

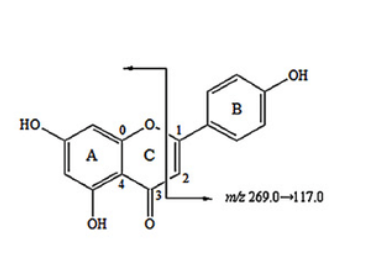

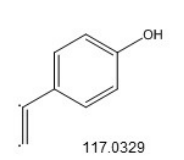

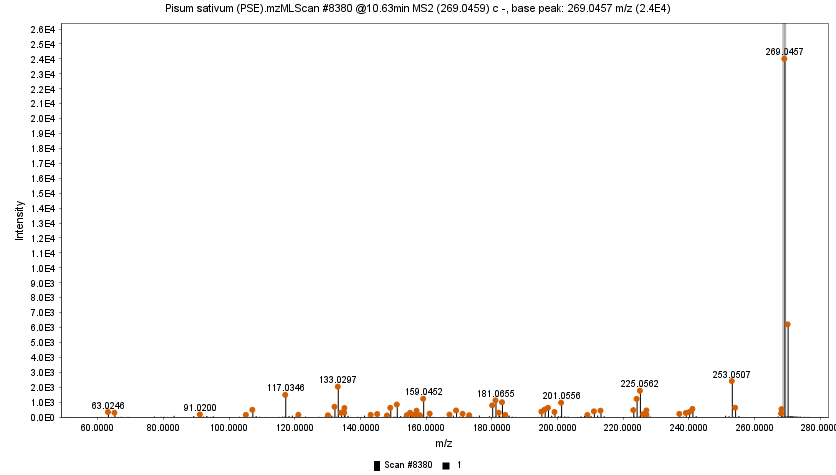


**Figure S15.** ESI-MS/MS spectrum of apigenin **(12)**.


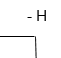

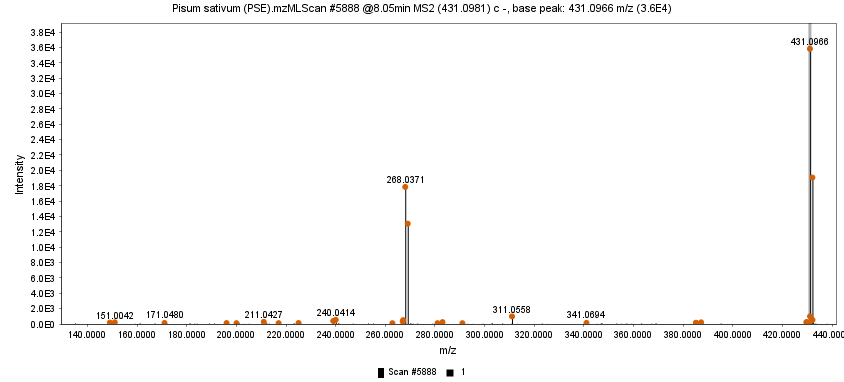


**Figure S16.** ESI-MS/MS spectrum of apigenin-7-*O*-glucoside **(13)**.


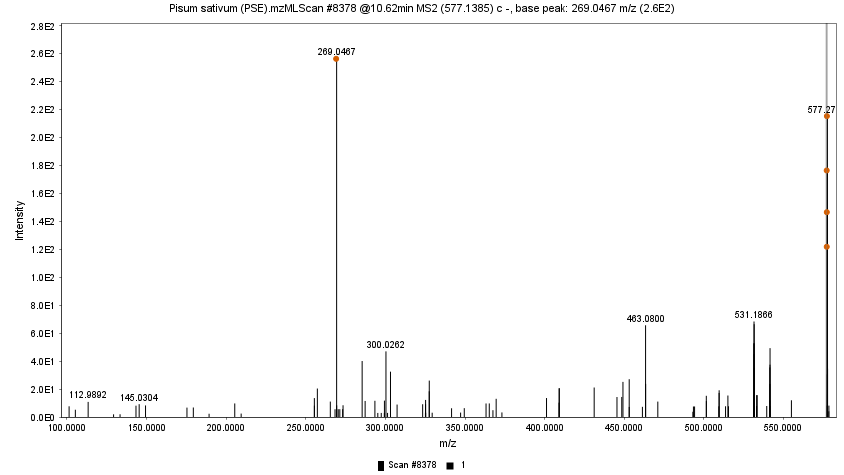


**[M-H-CO_2_]^-^**

**Figure S17.** ESI-MS/MS spectrum of apigenin 7-*O*-neohesperidoside **(14)**.


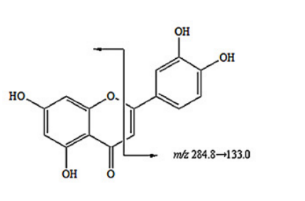

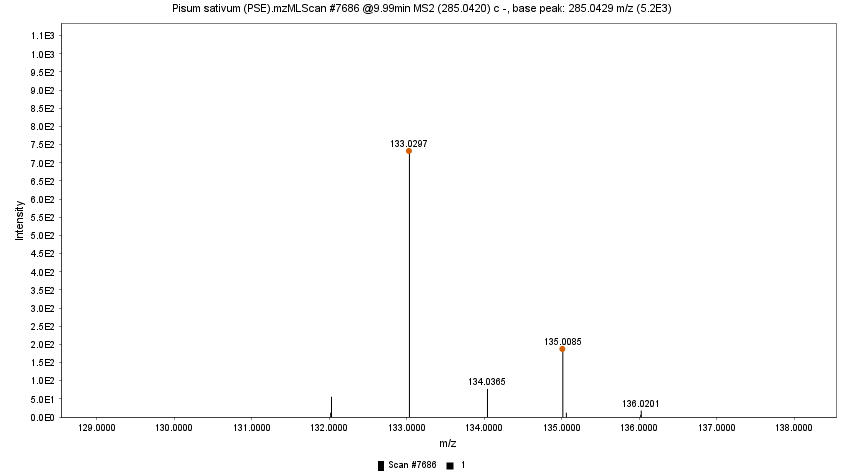

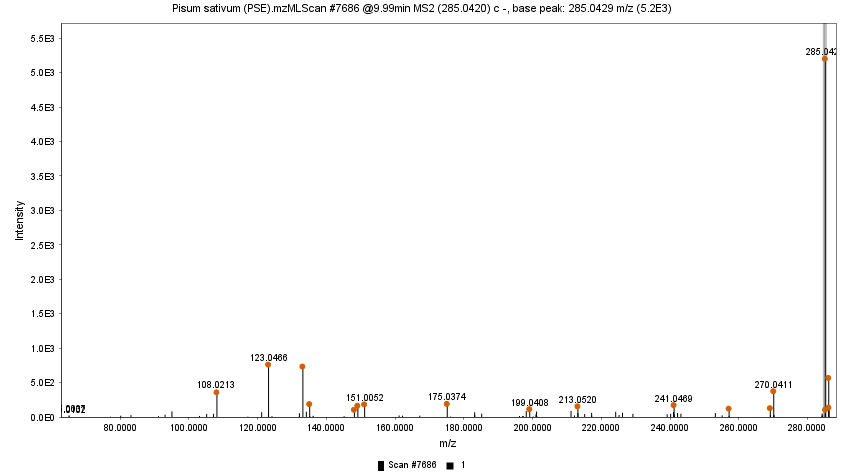


**Figure S18.** ESI-MS/MS spectrum of luteolin **(15)**.


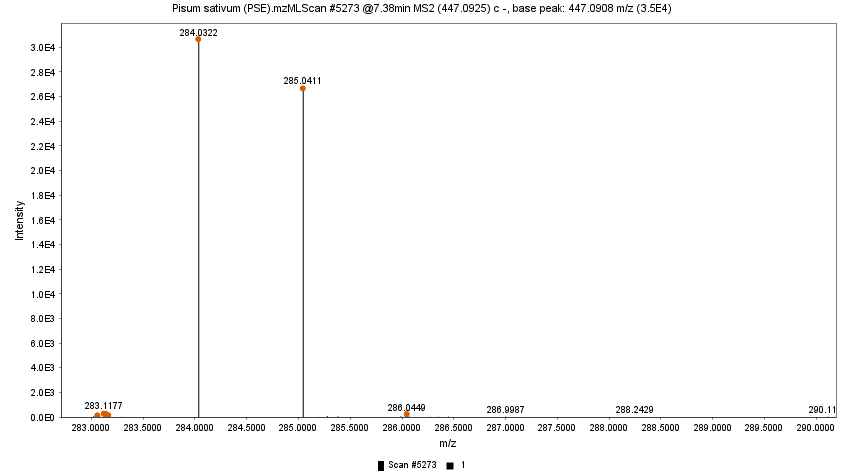

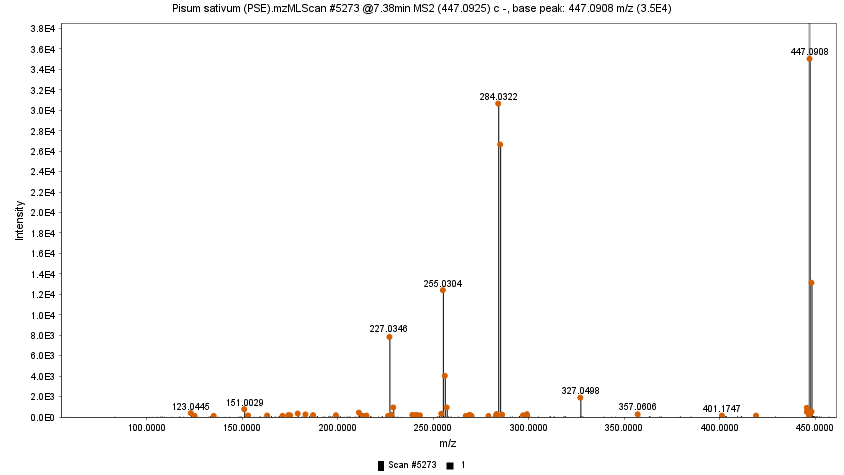


**285.0411**

**Figure S19.** ESI-MS/MS spectrum of luteolin-7-*O*-glucoside **(16)**.


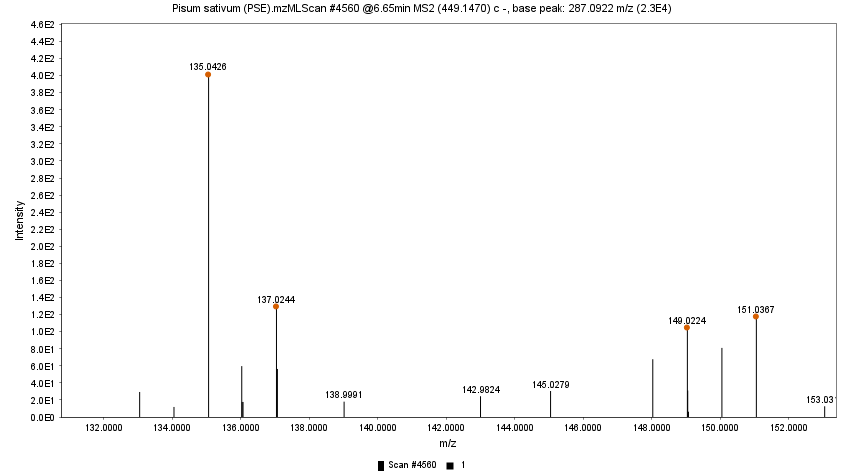

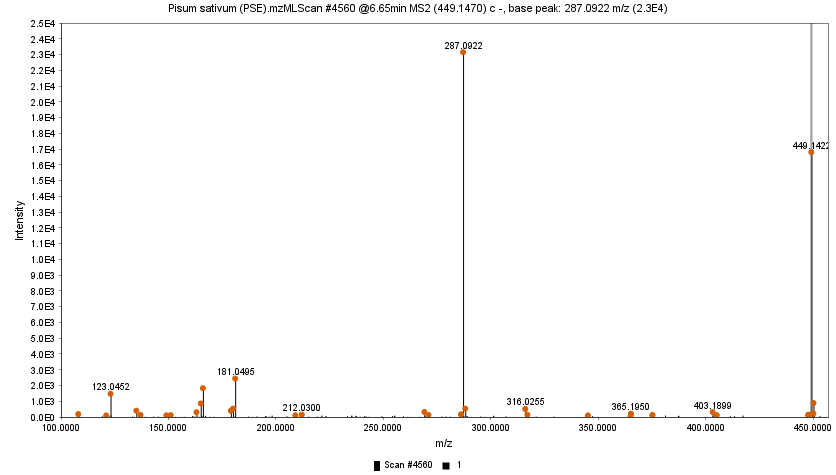


**287.0922**

**151.0367**

**135.0426**

**Figure S20.** ESI-MS/MS spectrum of eriodictyol-7-*O*-glucoside **(17)**.


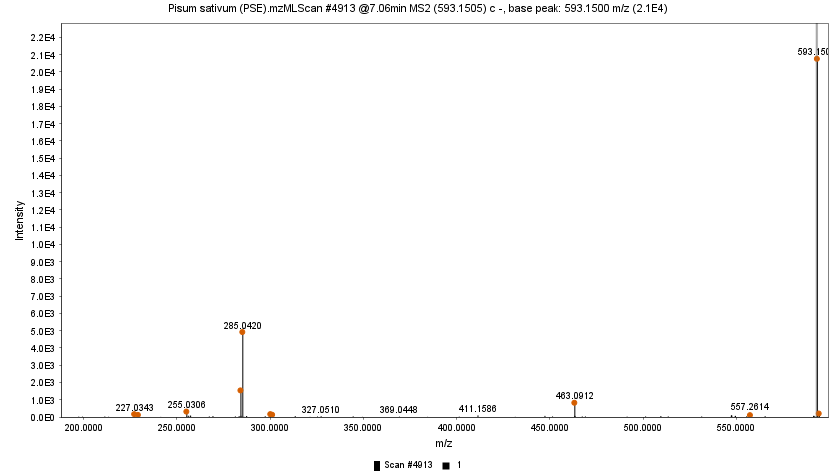


**Figure S21.** ESI-MS/MS spectrum of isosakuranetin-7-*O*-neohesperidoside **(18)**.


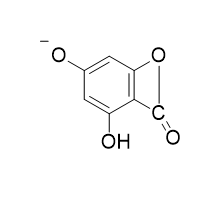

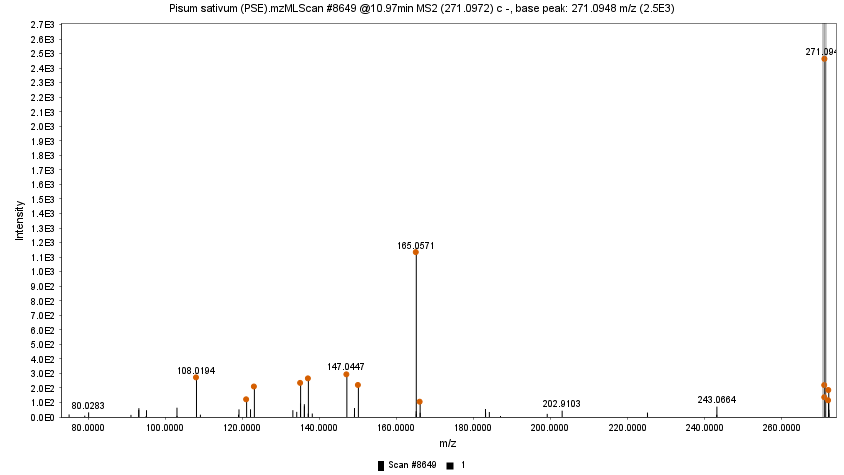


**119.0499**

**151.006**

**Figure S22.** ESI-MS/MS spectrum of naringenin **(19)**.


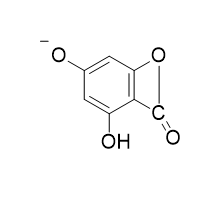

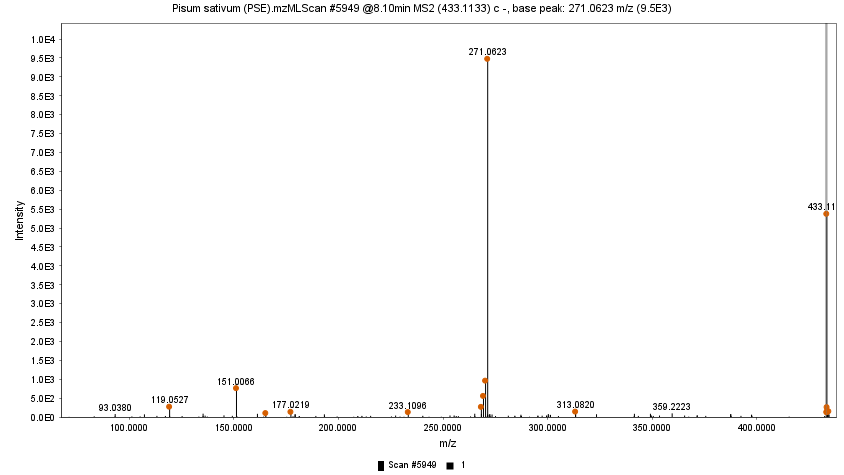


**271.0623**

**119.0527**

**Figure S23.** ESI-MS/MS spectrum of naringenin-7-*O*-glucoside **(20)**.


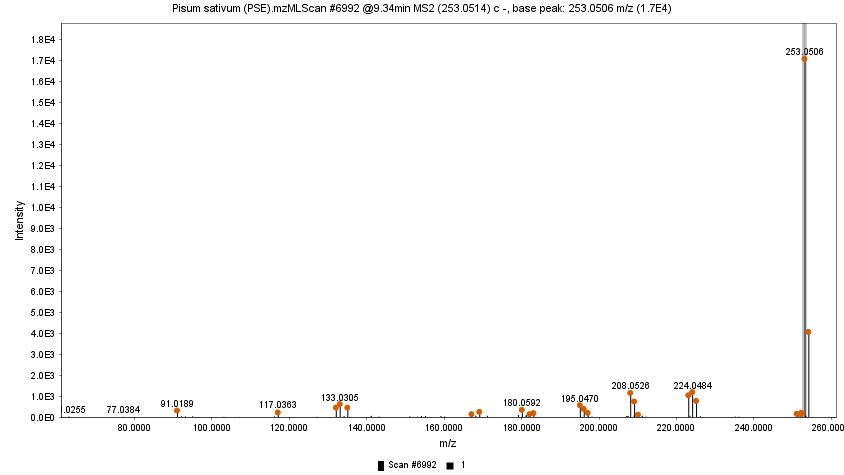


**135.0076**

**Figure S24.** ESI-MS/MS spectrum of daidzein **(21)**.


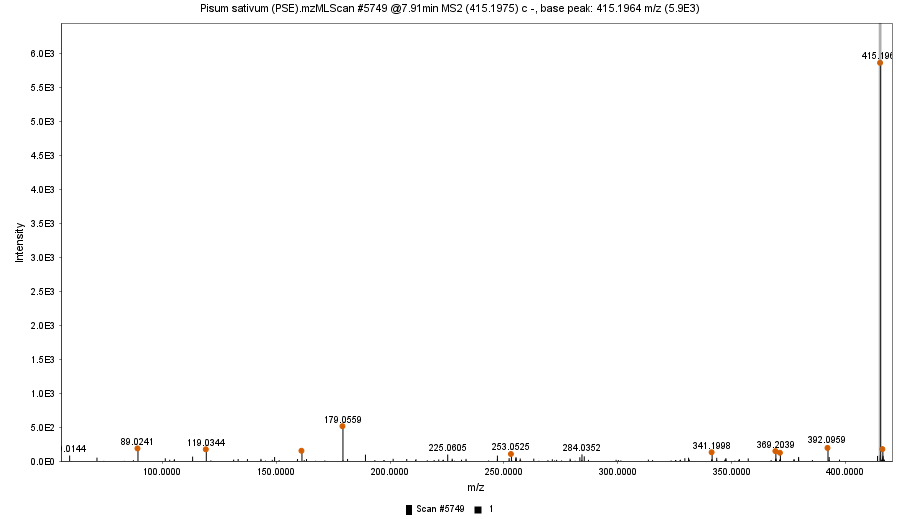


**Figure S25.** ESI-MS/MS spectrum of daidzein-8-*C*-glucoside **(22)**.


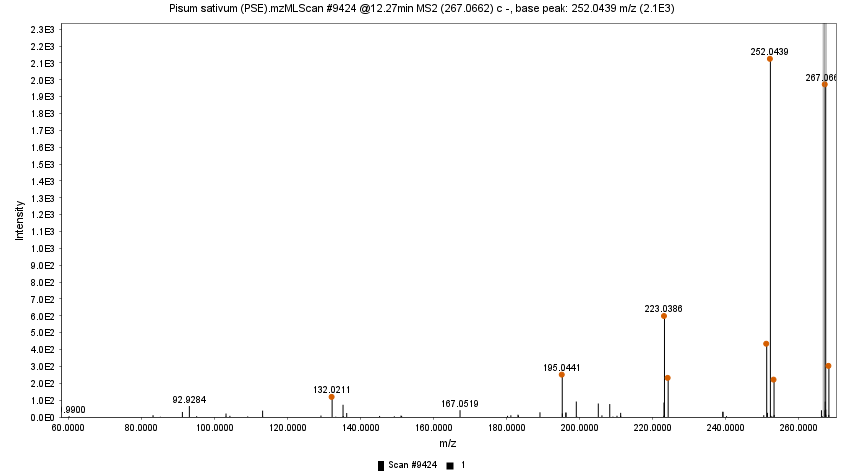


**Figure S26.** ESI-MS/MS spectrum of formononetin **(23)**.


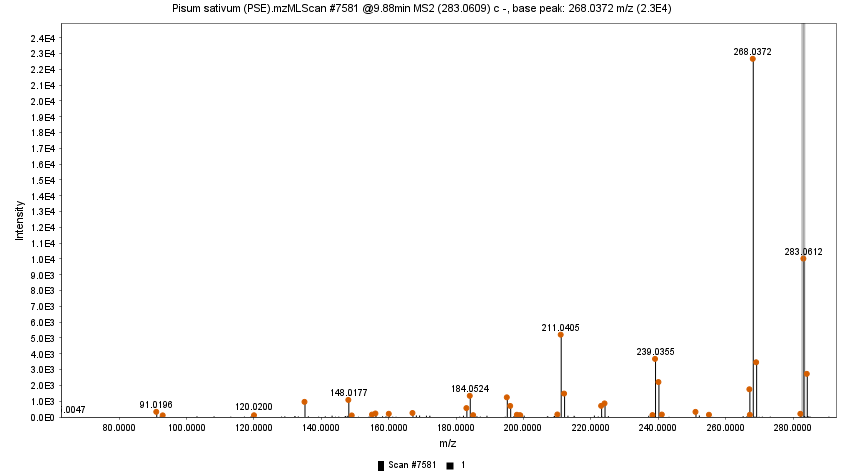


**268.0372**

**Figure S27.** ESI-MS/MS spectrum of glycitein **(24)**.


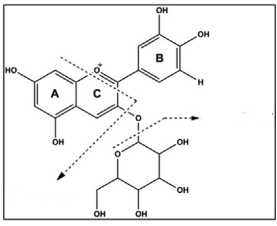

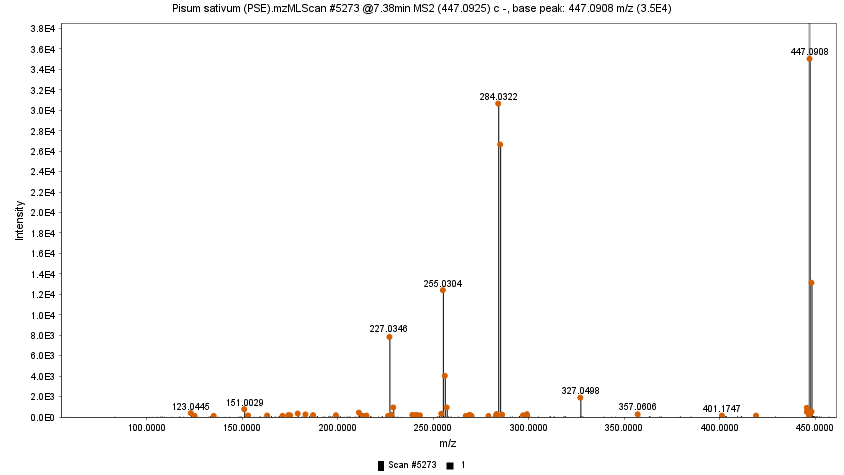


**135.0067**

**285.0411**

**Figure S28.** ESI-MS/MS spectrum of cyanidin-3-*O*-glucoside **(25)**.


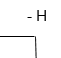

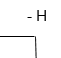

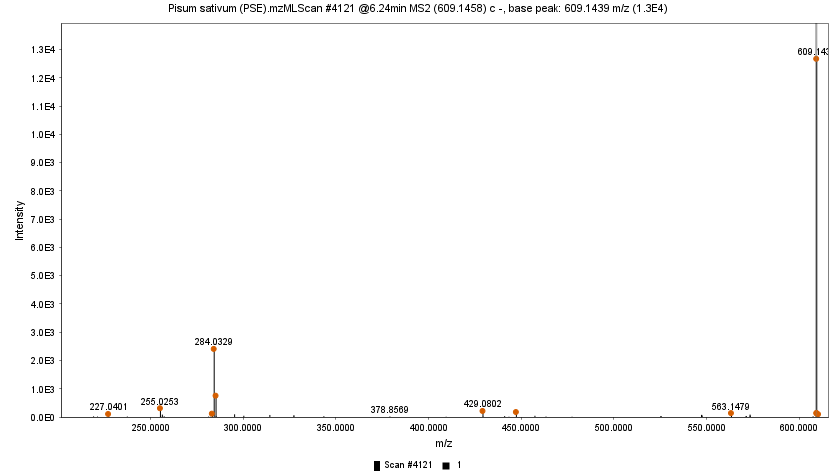


**284.0329**

**Figure S29.** ESI-MS/MS spectrum of cyanidin-3,5-di-*O*-glucoside **(26)**.


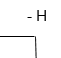

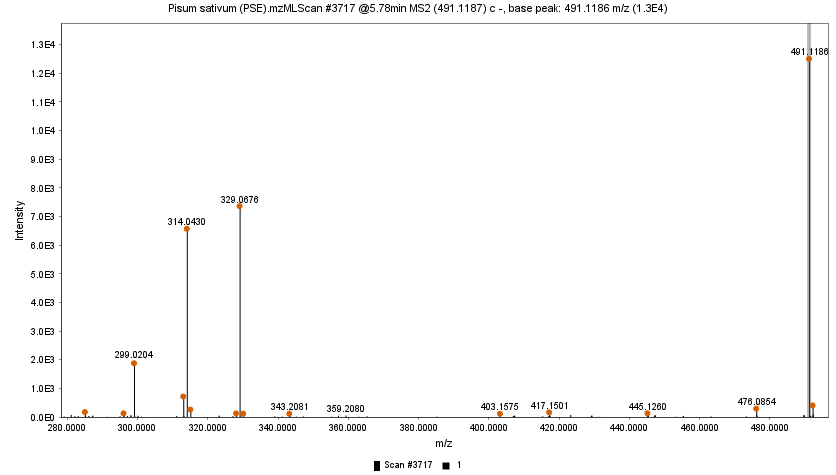


**329.0676**

**Figure S30.** ESI-MS/MS spectrum of malvidin-3-*O*-glucoside **(27)**.


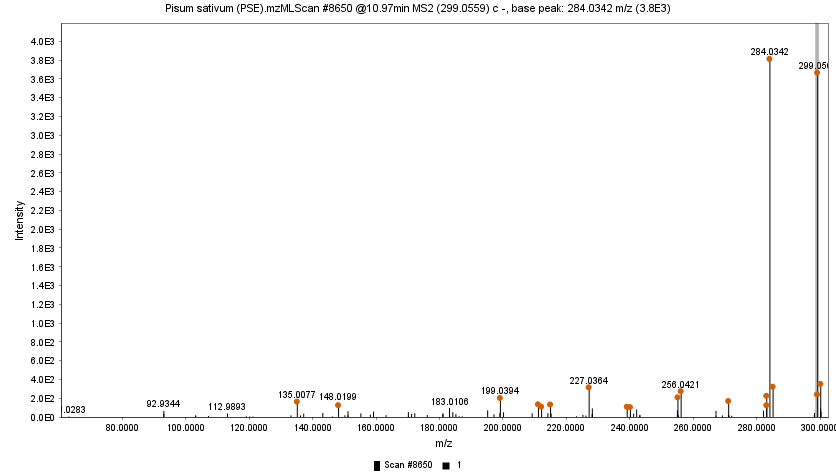


**[M-CH_3_-3CO-3H] ^–^**

NNNN

**[M-2H-CH_3_-2CO]^. –^**

**[M-2HCH_3_-CO]^. –^**

**284.0342**

**Figure S31.** ESI-MS/MS spectrum of peonidin **(28)**.


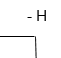

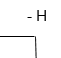

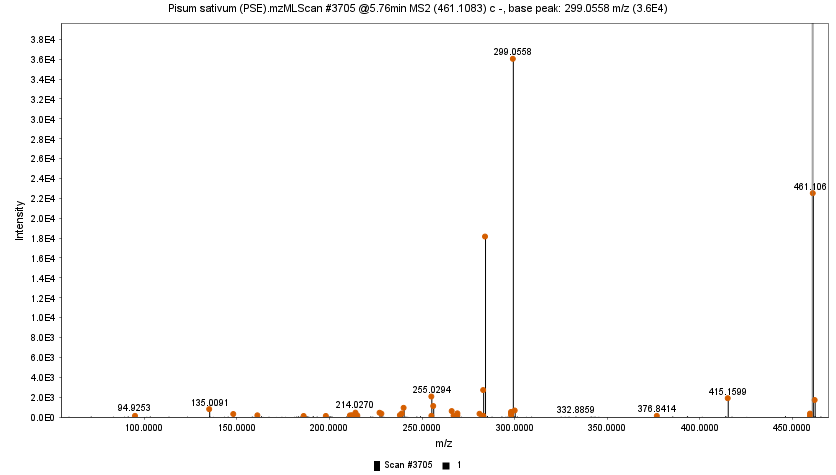


**Figure S32.** ESI-MS/MS spectrum of peonidin-3-*O*-glucoside **(29)**.


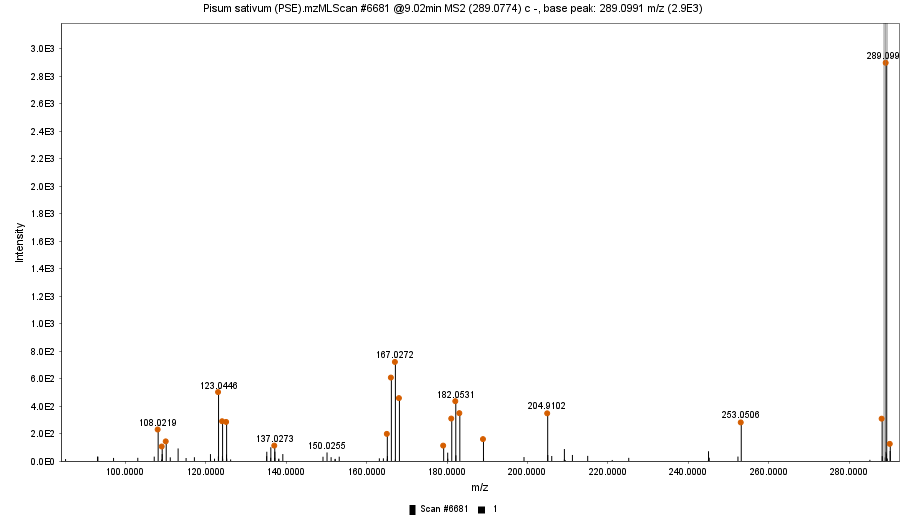


**137.0273**

**Figure S33.** ESI-MS/MS spectrum of catechin or epicatechin **(30)**.

[M − H−CO_2_] ^−^


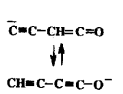

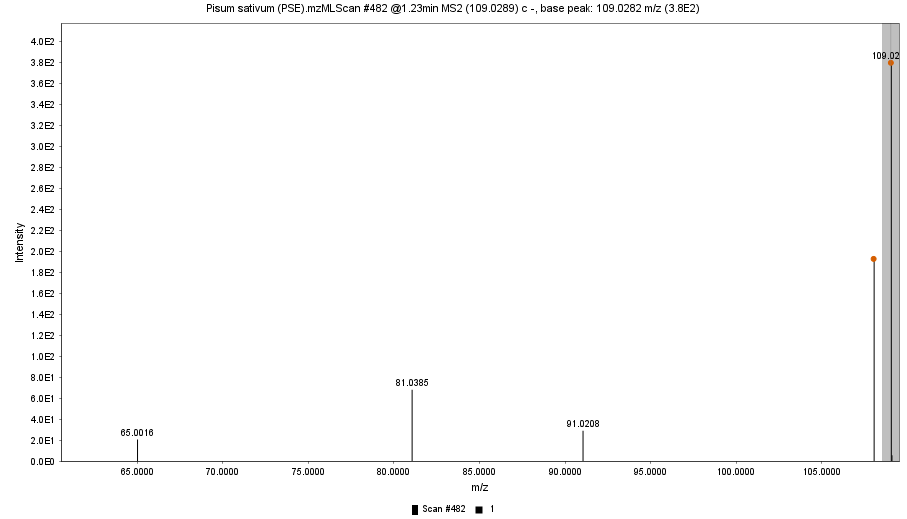


**[M-H-CO]^-^**

**[M-H-H2O]^-^**

**Figure S34.** ESI-MS/MS spectrum of 1,2-benzenediol **(31)**.


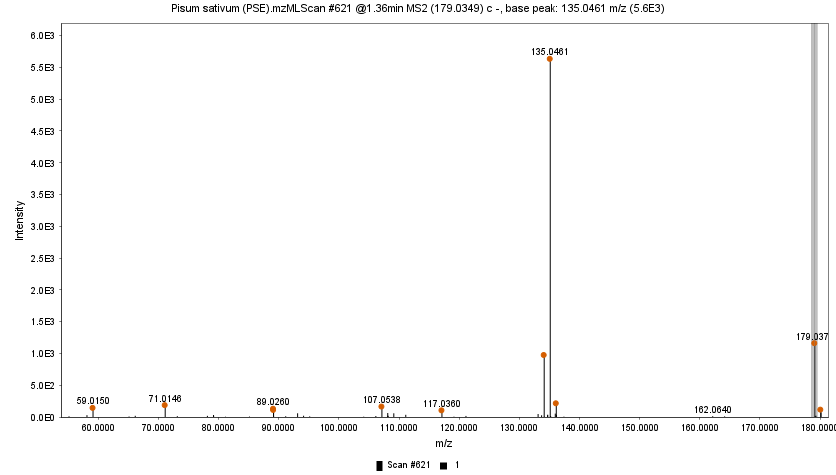


**[M-COOH]^-^**

**135.0461**

**Figure S35.** ESI-MS/MS spectrum of caffeic acid **(32)**.


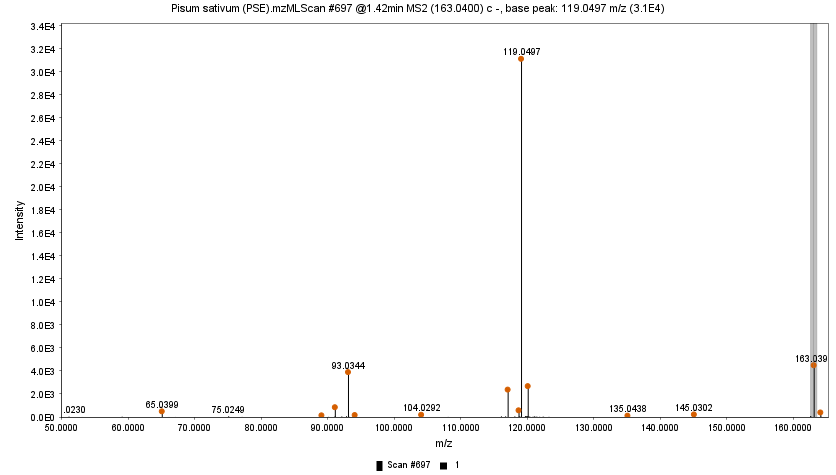


**Figure S36.** ESI-MS/MS spectrum of *p*-coumaric acid **(33)**.


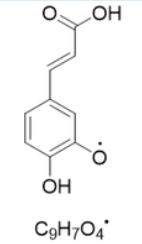

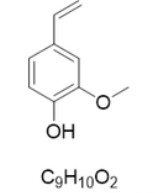

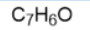

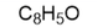

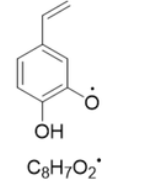

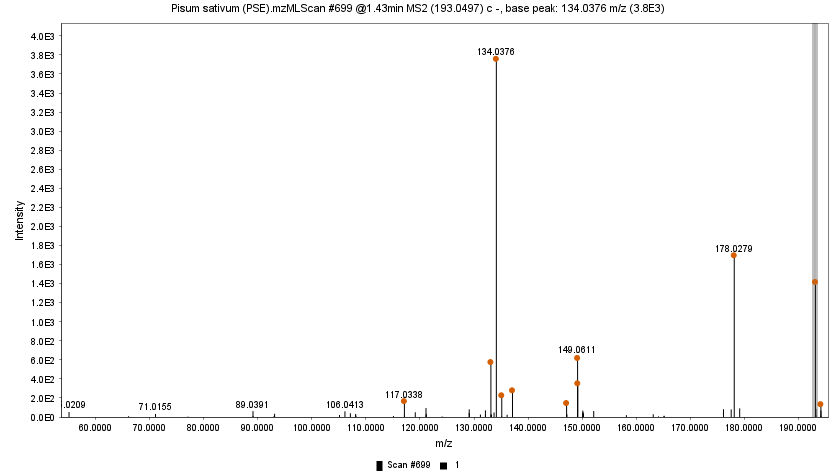


**178.0279**

**149.0611**

**134.0376**

**Figure S37.** ESI-MS/MS spectrum of ferulic acid **(34)**.


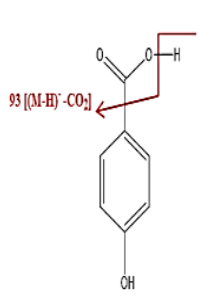

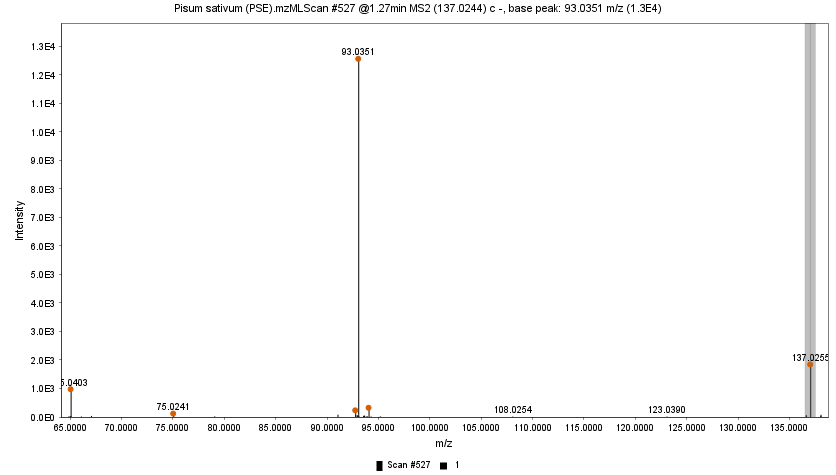


**93.0351**

**Figure S38.** ESI-MS/MS spectrum of *p*-hydroxy benzoic acid **(35)**.

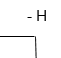

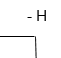

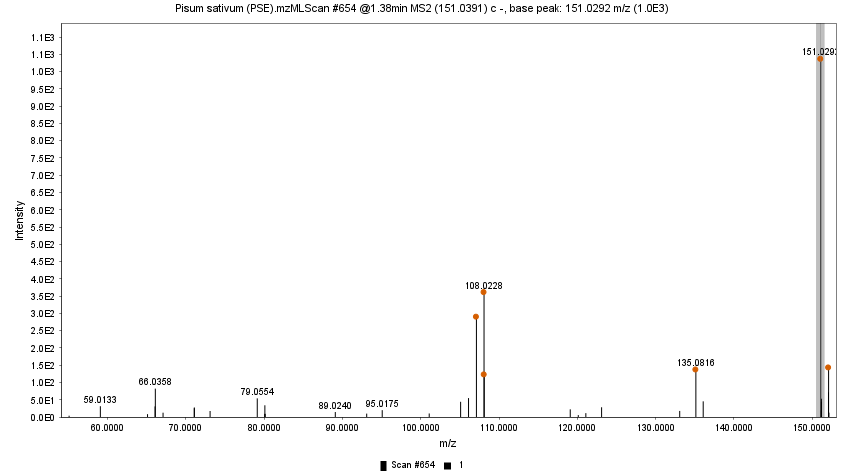


**Figure S39.** ESI-MS/MS spectrum of *p*-hydroxyphenyl acetic acid **(36)**.


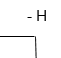

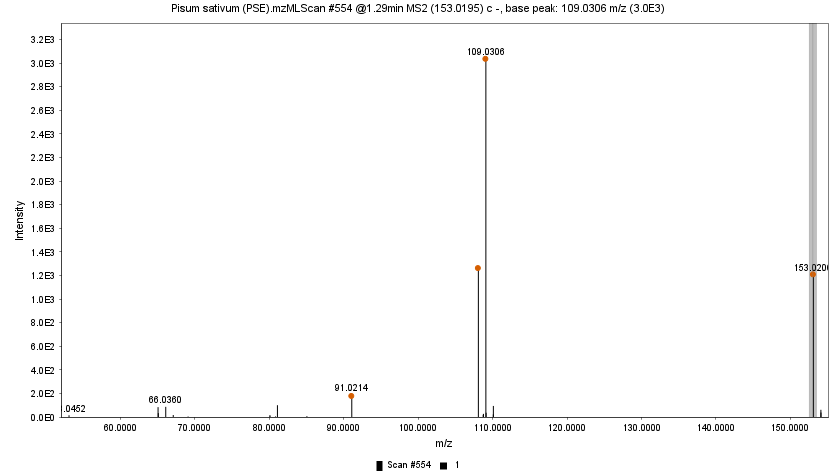


**Figure S40.** ESI-MS/MS spectrum of protocatechuic acid **(37)**.


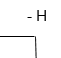

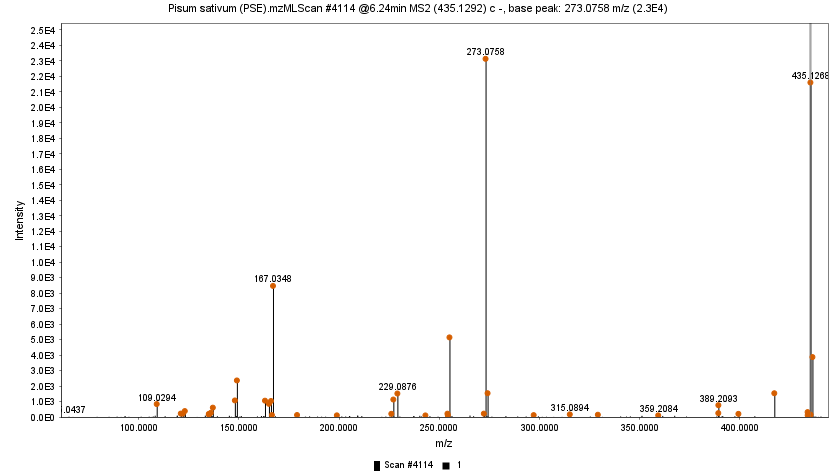


**273.0758**

**Figure S41.** ESI-MS/MS spectrum of phloridzin **(38)**.


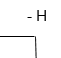

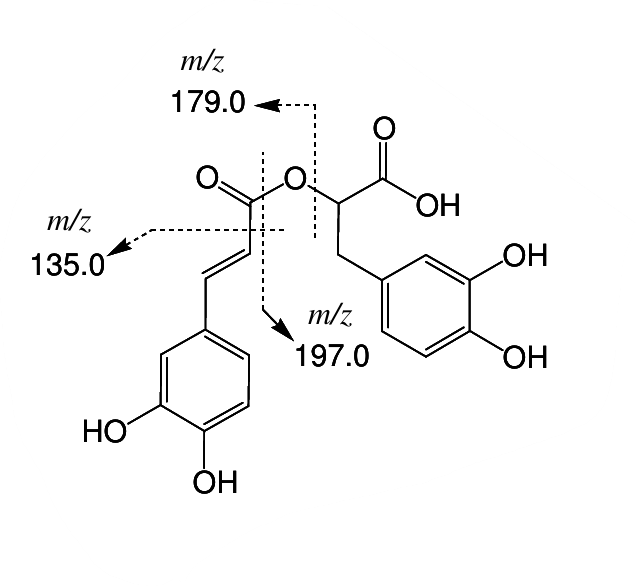

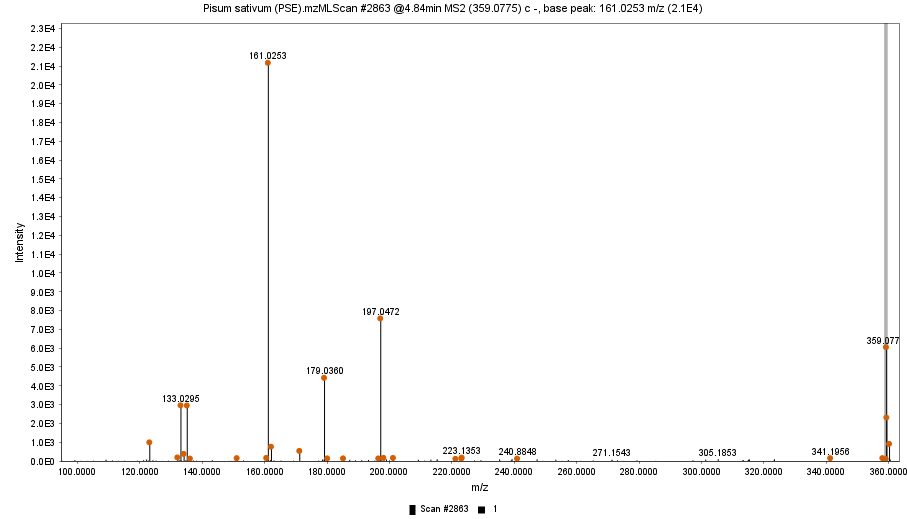
**Figure S42.** ESI-MS/MS spectrum of rosmarinic acid **(39)**.

**[179-H2O]**

**135.0453**


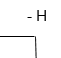

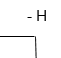

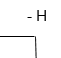

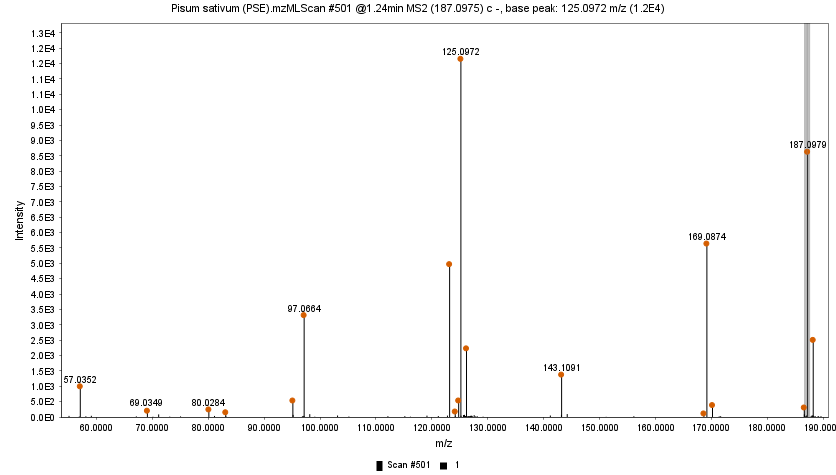


**Figure S43.** ESI-MS/MS spectrum of azelaic acid **(40)**.


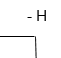

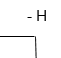

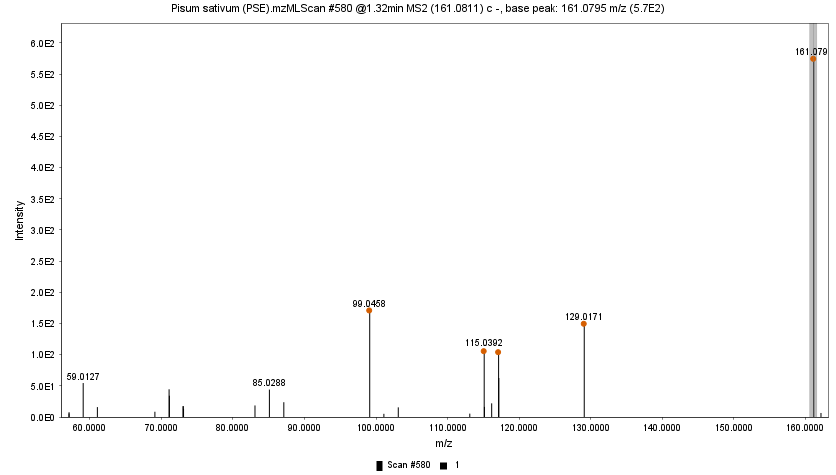


**59.0127**

**103.0352**

**Figure S44.** ESI-MS/MS spectrum of 3-hydroxy-3-methylglutaric acid **)41**(.


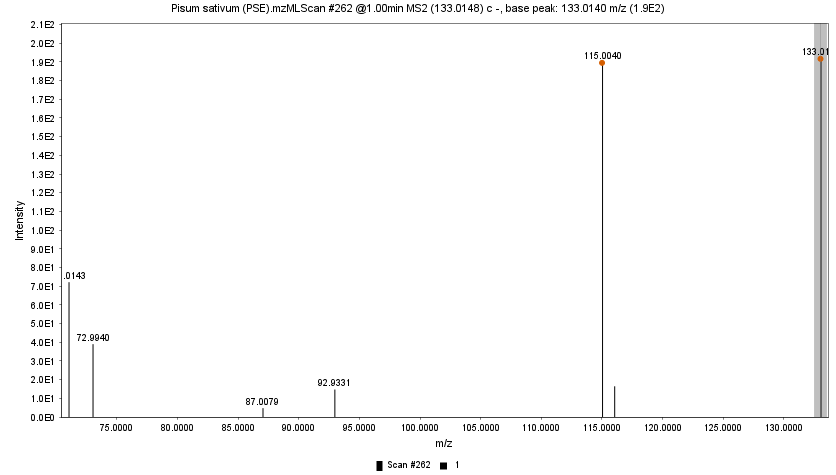


**[M-H_2_O]^-^**

**Figure S45.** ESI-MS/MS spectrum of D- (+)-malic acid **(42)**.

**2.4. Characterization of the pure compounds isolated from the petroleum ether (PSP) and ethyl acetate (PSE) fractions of *P. sativum* L.**

***1-Eicosanol* (1):** White powder (15 mg), R*_f_* = 0.54 using silica gel TLC and *n*-hexane-EtOAc (8:2, v/v) as eluting system, producing a purple color with vanillin/sulfuric acid spray reagent after heating at 110°C for 1 min. ESI-MS displayed a significant molecular ion peak [M]^+^ at *m/z* 298.6, corresponding to the molecular formula C_20_H_42_O. Its structure was identified by comparing its spectroscopic data with that reported in literature [84]. ^1^H-NMR (500 MHz, pyridine-d5) *δ*_H_: 3.88 (2H, t, H-1),1.74 (2H, m, H-2), 1.49 (2H, m, H-3), 1.25 -1.32 (16H, H-4-19), 0.87 (3H, t, *J* = 6.4, H-20). ^13^C-NMR (125 MHz, pyridine-d_5_) *δ*_C_: 62.09 (C-1; CH_2_), 33.74 (C-2; CH_2_), 32.07 (C-3; CH_2_), 29.49-29.94 (C-4-17; CH_2_), 26.49 (C-18; CH_2_), 22.89 (C-19; CH_2_), 14.23 (C-20; CH_3_).

***Stigmasterol* (2)**: White powder (50 mg), R*_f_* = 0.37 using silica gel TLC and *n*-hexane-EtOAc (8:2, v/v) as eluting system, producing a purple color with vanillin/sulfuric acid spray reagent after heating at 110°C for 1 min. EI-MS displayed significant peaks at *m/z*: 413 [M]^+^, 396, 272, 144, 106, corresponding to the molecular formula C_29_H_48_O. Its structure was identified by comparing its spectroscopic data with that reported in literature [85, 86]. ^1^H-NMR (CDCl_3_, 400 MHz) *δ*_H_: 3.54 (1H, tdd, *J* = 4.5, 4.2, 3.8 Hz, H-3), 5.36 (1H, d, *J* = 4.8 Hz, H-6), 0.93 (3H, d, *J* = 6 Hz, H-19), 5.04 (1H, m, H-20), 5.14 (1H, m, H-21), 0.83 (3H, overlapped, H-24), 0.83 (3H, overlapped, H-26), 0.83 (3H, overlapped, H-27), 0.70 (3H, s, H-28), 1.03 (3H, s, H-29). ^13^C-NMR (100 MHz, CDCl_3_-*d*) *δ*_C_: 37.27 (C-1; CH_2_), 31.67 (C-2; CH_2_), 71.82 (C-3; CH), 42.31 (C-4; CH_2_), 140.77 (C-5; C), 121.73 (C-6; CH), 31.93 (C-7; CH_2_), 31.93 (C-8; CH), 50.15 (C-9; CH), 36.52 (C-10; C), 21.10 (C-11; CH_2_), 39.79 (C-12; CH_2_), 42.26 (C-13; C), 56.78 (C-14; CH), 24.31 (C-15; CH_2_), 28.26 (C-16; CH_2_), 56.02 (C-17; CH), 40.50 (C-18; CH), 21.10 (C-19; CH_3_), 138.33 (C-20; CH), 129.29 (C-21; CH), 45.85 (C-22; CH), 26.10 (C-23; CH_2_), 11.87 (C-24; CH_3_), 29.17 (C-25; CH), 19.83 (C-26; CH_3_), 19.41 (C-27; CH_3_), 18.99 (C-28; CH_2_), 12.08 (C-29; CH_3_).

***Anhydropisatin* (3)**: this compound was isolated as colorless needle crystals (30 mg), R_ƒ_ = 0.35 using normal silica gel TLC and *n*-hexane-CH_2_Cl_2_ (1:1, v/v) as eluting system, quenched UV_254_ light, producing orange color with vanillin/sulfuric acid spray reagent after heating at 110°C for 1 min. EI-MS displayed significant peaks at *m/z* (% relative intensity): 295.7 (100), 294.9 (93.65), 294.2 (68.55), 222.5 (23.65), 251.2 (14.12), corresponding to the molecular formula C_17_H_14_O_6_. Its structure was identified by comparing its spectroscopic data with that reported in literature [87]. ^1^H-NMR (500 MHz, CDCl_3_-*d*) *δ*_H_: 7.33 (1H, d, *J* = 9 Hz, H-1), 6.50 (1H, dd, *J* = 8.5 Hz, 2.5 Hz, H-2), 6.47 (1H, d, *J* =2.5 Hz, H-4), 5.49 (2H, s, H-6), 6.99 (1H, S, H-7), 5.97 (2H, s, H-9), 6.70 (1H, s, H-11), 3.76 (3H, s, OCH_3_). ^13^C-NMR ( 125 MHz, CDCl_3_-*d*) *δ*_C_: 120.96 (C-1; CH), 107.25 (C-2; CH), 160.87 (C-3; C), 94.14 (C-4; CH), 154.97 (C-4a; C), 65.54 (C-6; -OCH_2_O-), 109.94 (C-6a; C), 119.21 (C-6b; C), 102.52 (C-7; CH), 144.78 (C-7a; C), 101.47 ( C-9; CH_2_), 145.68 (C-10a; C), 97.33 (C-11; CH), 147.74 (C-11a; C), 150.51 (C-12a; C), 106.41 (C-12b; C), 55.49 (OCH_3_).

***Pisatin* (4)**: Yellowish white semisolid (70 mg), R*_f_* = 0.38 using normal silica gel TLC and petroleum ether-EtOAc (7:3, v/v) as eluting system, quenched UV_254_ light, producing an orange color with vanillin/sulfuric acid spray reagent after heating at 110°C for 1 min. EI-MS spectrum showed [M]^+^ signal at 314.38, corresponding to the molecular formula C_17_H_14_O_6_. Its structure was identified by comparing its spectroscopic data with that reported in literature [88]. ^1^H-NMR (400 MHz, CDCl_3_-*d*) *δ*_H_: 7.37 (1H, d, *J* = 8.4 Hz, H-1) , 6.65 (1H, dd, *J* = 8.8 Hz, 2.4 Hz, H-2), 6.45 (1H, d, *J* = 2.4 Hz, H-4), 4.00 (1H, d, *J* = 12 Hz, H-6_1_), 4.17(1H, d, *J* = 11.6 Hz, H-6_2_), 6.79 (1H, s, H-7), 5.91 (2H, *J* = 1.2 Hz, H-9), 6.39 (1H, s, H-11), 5.27 (1H, s, H-12a), 3.78 (3H, s, -OCH_3_).^13^C-NMR (100 MHz, CDCl_3_-*d*) *δ*_C_: 131.83 (C-1; C), 103.05 (C-2; CH), 161.04 (C-3; C), 94.20 (C-4; CH), 155.71 (C4a; C), 69.68 (C-6; OCH_2_O), 77.07 (C- 6a; C), 118.98 (C-6b; CH), 109.81 (C-7; CH), 142.40 (C-7a; C), 101.57 (C-9; CH_2_), 149.80 (C-10a; C), 101.62 (C-11; CH), 154.47 (C-11a; C), 84.89 (C-12a; CH), 112.41(C-12b; C), 55.53 (OCH_3_).

***p-Hydroxy benzoic acid* (5)**: White powder (25 mg), R*_f_* = 0.32, using silica gel TLC and CH_2_Cl_2_-MeOH (9.5:0.5, v/v) as eluting system and quenched UV_254_ light), C_7_H_6_O_3_. EI-MS showed [M]^+^ signal at 138.00, corresponding to the molecular formula C_7_H_6_O_3_**.** Its structure was identified by comparing its spectroscopic data with that reported in literature [89]. ^1^H-NMR (400 MHz, CD_3_OD) *δ*_H_: 7.78 (2H, d, *J* = 8.4 Hz, H-2,6), 6.72 (2H, d, *J* = 8.4 Hz, H-3, 5). ^13^C-NMR (100 MHz, CD_3_OD) *δ*_C_: 121.86 (C-1; C), 131.58 (C-2, 6; CH), 114.59 (C-3, 5; CH), 161.77 (C-4; C), 168.18 (C-7; C).

***Quercetin 3-O-β-D-glucopyranoside (Isoquercetin,* 6)**: Yellow powder (20 mg), R*_f_* = 0.47, using silica gel TLC and EtOAc-MeOH-H_2_O (8:1:1, v/v) as eluting system, producing an orange color with vanillin/sulfuric acid spray reagent after heating at 110°C for 1 min. ESI-MS showed a significant peak at *m/z* 486.2 [M+Na]^+^, 462.4 [M-2H]^-^, corresponding to the molecular formula C_21_H_20_O_12_. Its structure was identified by comparing its spectroscopic data with that reported in literature [90]. ^1^H-NMR (CD_3_OD, 400 MHz) *δ*_H_: 6.10 (1H, br s, H-6), 6.29 (1H, br s, H-8), 7.61 (1H, br s, H-2), 6.77 (1H, d, *J* = 8 Hz, H-5′), 7.47 (1H, d, *J* = 8 Hz, H-6′), 5.12 (1H, d, *J* = 8 Hz, H-1''), 3.49 (1H, d, *J* = 8 Hz, H-2''), 3.46 (1H, d, overlapped, H-3''), 3.34 (1H, dd, *J* = 8, 12 Hz, H-4''), 3.25 (1H, m, H-5''), 3.60 (2H, d, *J* = 11.6 Hz, H-6''). ^13^C-NMR (100 MHz, CD_3_OD) *δ*_C_: 157.65 (C-2; C), 134.20 (C-3; C), 178.07 (C-4; C), 161.60 (C-5; C), 98.61 (C-6; CH), 164.79 (C-7; C), 93.42 (C-8; CH), 157.07 (C-9; C), 104.25 (C-10; C), 121.67 (C-1′; C), 114.66 (C-2′; CH), 144.51 (C-3′; C), 148.47 (C-4′; C), 116.19 (C-5′; CH), 121.83 (C-6′; CH), 102.92 (C-1′′; CH), 74.32 (C-2′′; CH), 76.69 (C-3′′; CH), 69.81 (C-4′′; CH), 76.95 (C-5′′; CH), 61.14 (C-6′′; CH_2_).

***Quercetin-3-O-(6****''''****-O-cis-p-coumaroyl)-sophorotrioside (Pisumflavonoside I,* 7)**: Yellow powder (30 mg), R*_f_* = 0.4 using silica gel TLC and EtOAc-MeOH-H_2_O (7.5:1.5:1, v/v) as eluting system, producing an orange color with vanillin/sulfuric acid spray reagent after heating at 110°C for 1 min. ESI-MS showed a significant peak at *m/z* 955.4 [M-2H+Na]^+^, 931.8 [M-2H]^-^, corresponding to the molecular formula C_42_H_46_O_24_. Its structure was identified by comparing its spectroscopic data with that reported in literature [34] .^1^H-NMR (CD_3_OD, 500 MHz) *δ*_H_: 6.16 (1H, br s, H-6), 6.31 (2H, overlapped, H-8), 7.57 (1H, br s, H-2′), 6.85 (1H, d, *J* = 8 Hz, H-5′), 7.61 (1H, d, *J* = 8.Hz, H-6′), 5.34 (1H, br s, H-1′′), 3.48 (1H, d, *J* = 4 Hz, H- 2′′), 3.55 (1H, dt, H-3''), 3.59 (1H, t, H, H-4''), 3.55 (1H, overlapped, H-5′′,), 3.57 (1H, overlapped, H-6''a), 3.37 (1H, d, *J* = 8 Hz, H-6''b), 4.63 (1H, d, *J* = 8 Hz, H-1′′′), 3.22 (1H, m, H-2'''), 3.66 (1H, s, H-3′′′), 3.59 (1H, t, H-4'''), 3.55 (1H, overlapped, H-5'''), 3.57 (1H, overlapped, H-6'''a), 3.41 (1H, d, *J* = 8 Hz, H-6'''b), 4.53 (1H, d, *J* = 8 Hz, H-1''''), 3.14 (1H, m, H-2''''), 3.66 (1H, overlapped, H-3''''), 3.59 (1H, t, H-4''''), 3.81 (1H, s, H-5''''), 4.23 (1H, dd, *J* = 8, 4 Hz, H-6''''a), 4.18 (1H, m, H-6''''b), 6.31 (2H, overlapped, H-C2), 7.46 (1H, d, *J* = 8 Hz, H-C3), 7.40 (2H, d, *J* = 8 Hz, H-C2', H-C6'), 6.69 (2H, d, *J* = 8 Hz, H-C3', H-C5'). ^13^C-NMR (125 MHz, CD_3_OD) *δ*_C_: 156.08 (C-2; C), 132.68 (C-3; C), 178.40 (C-4; C), 160.00 (C-5; C), 99.93 (C-6; CH), 164.46 (C-7; C), 93.72 (C-8; CH), 157.13 (C-9; C), 104.18 (C-10; C), 121.64 (C-1′; C), 116.59 (C-2′; CH), 144.64 (C-3′; C), 148.53 (C-4′; C), 114.91 (C-5′; CH), 121.71 (C-6′; CH), 99.00 (C-1′′; CH), 83.04 (C-2′′; CH), 76.24 (C-3′′; CH), 69.74 (C-4′′; CH), 76.55 (C-5′′; CH), 61.08 (C-6′′; CH_2_), 102.50 (C-1′′′; CH), 83.81 (C-2′′′; CH), 76.36 (C-3′′′; CH), 68.84 (C-4′′′; CH), 76.92 (C-5′′′; CH), 60.71 (C-6′′′; CH_2_), 104.98 (C-1′′′′; CH), 74.82 (C-2′′′′; CH), 76.24 (C-3′′′′; CH), 70.09 (C-4′′′′; CH), 74.82 (C-5′′′′; CH), 63.29 (C-6′′′′; CH_2_), 167.82 (C-C1; C), 113.74 (C-C2; CH), 145.78 (C-C3; CH), 125.73 (C-C1′; C), 130.08 (C-C2′; CH), 115.52 (C-C3′; CH), 157.37 (C-C4′; C), 115.52 (C-C5′; CH), 130.08 (C-C6′; CH).


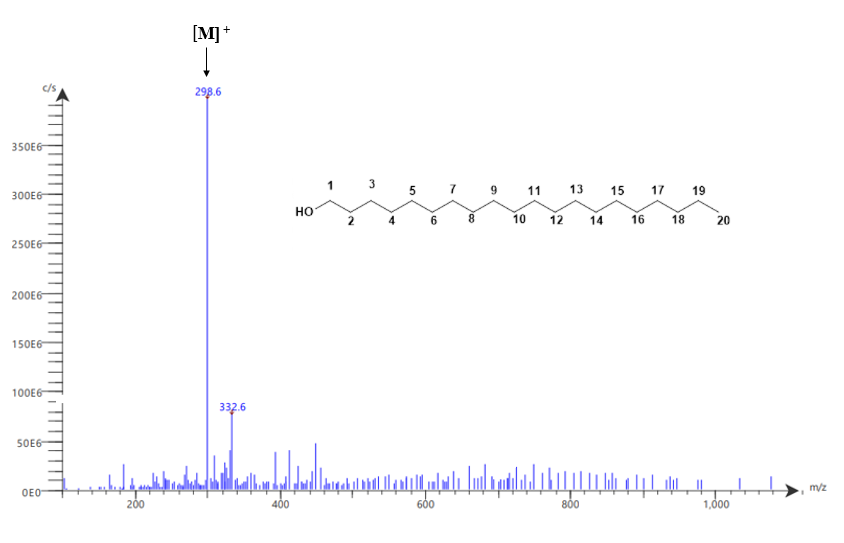


**Figure S46.** ESI-MS spectra of compound **1** (1-Eicosanol), showing [M]+ ion peak at m/z 298.6.

**
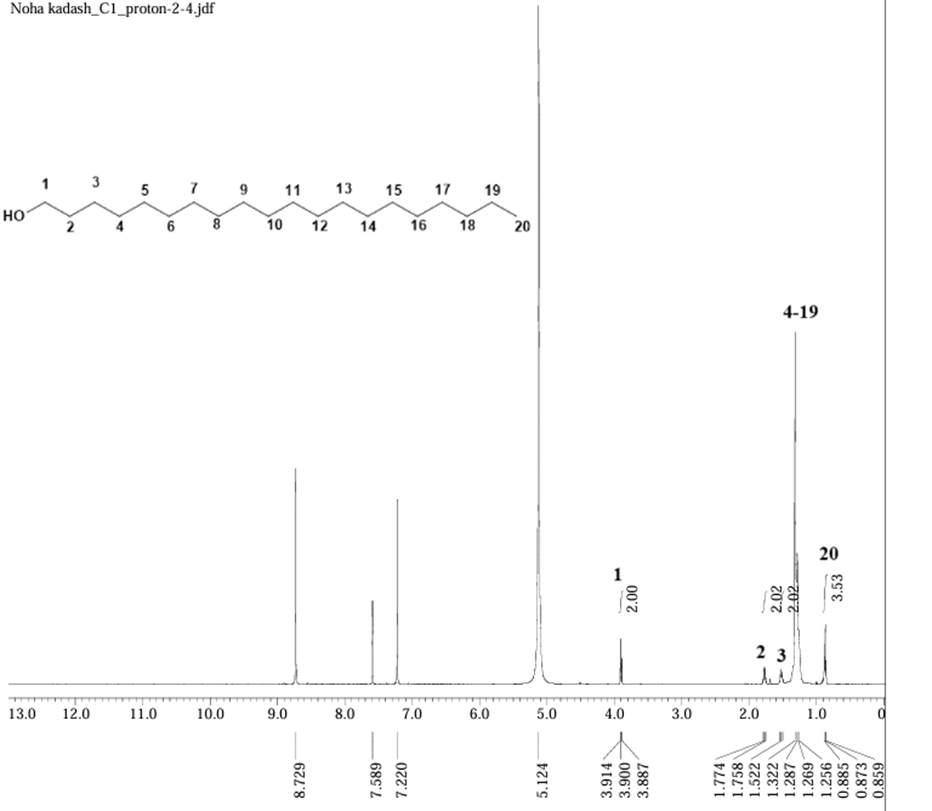
**

**Figure S47.**^1^H-NMR spectrum (500 MHz, pyridine-d5) of compound **1** (1-Eicosanol).


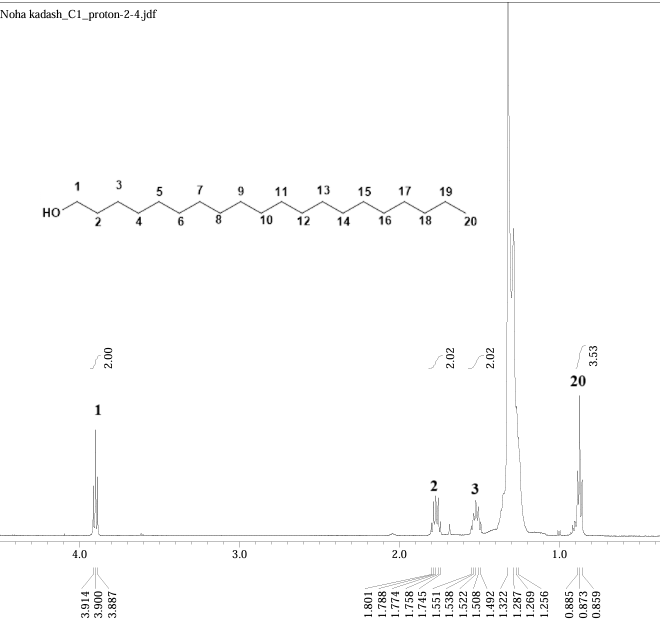


**Figure S48.** Expansions of ^1^H-NMR spectrum (500 MHz, pyridine-d5) of compound **1** (1-Eicosanol) from *δ* 0.5−4.4 ppm.


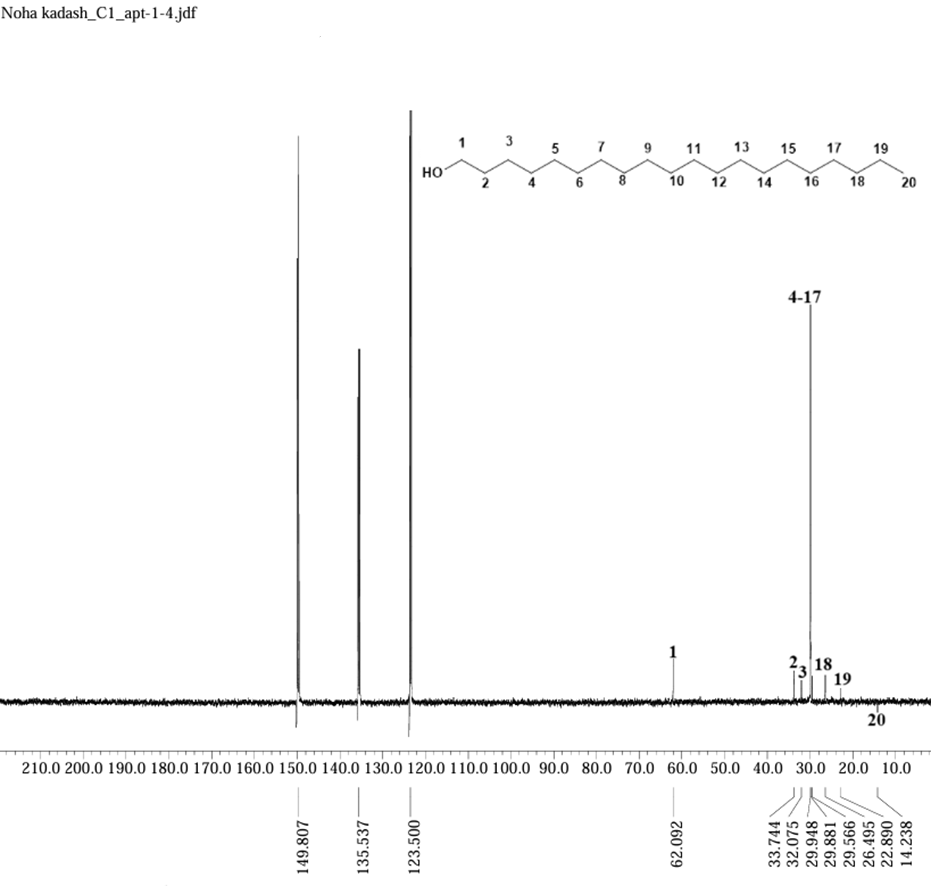
**Figure S49.** APT spectrum (125 MHz, Pyridine-d5) of compound **1** (1-Eicosanol).


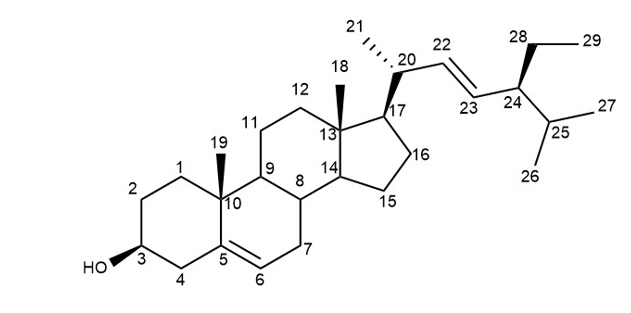

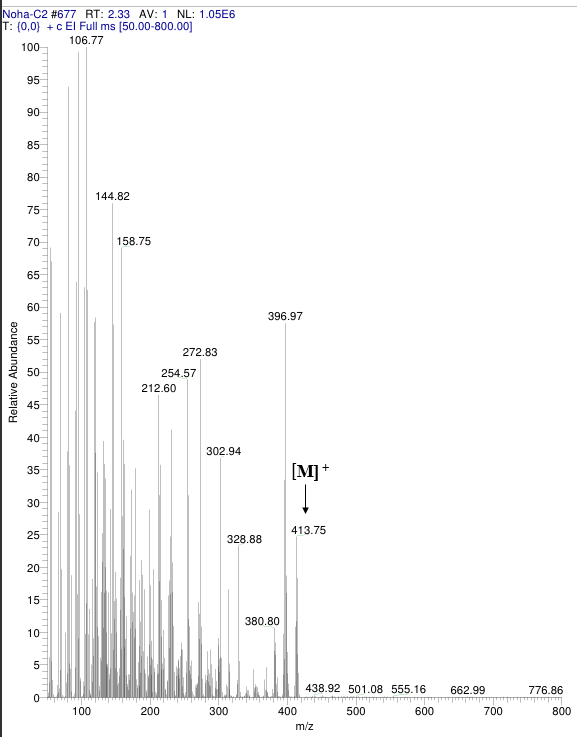


**Figure S50.** EI-MS spectra of compound **2** (Stigmasterol), showing [M]^+^ ion peak at *m/z* 413.


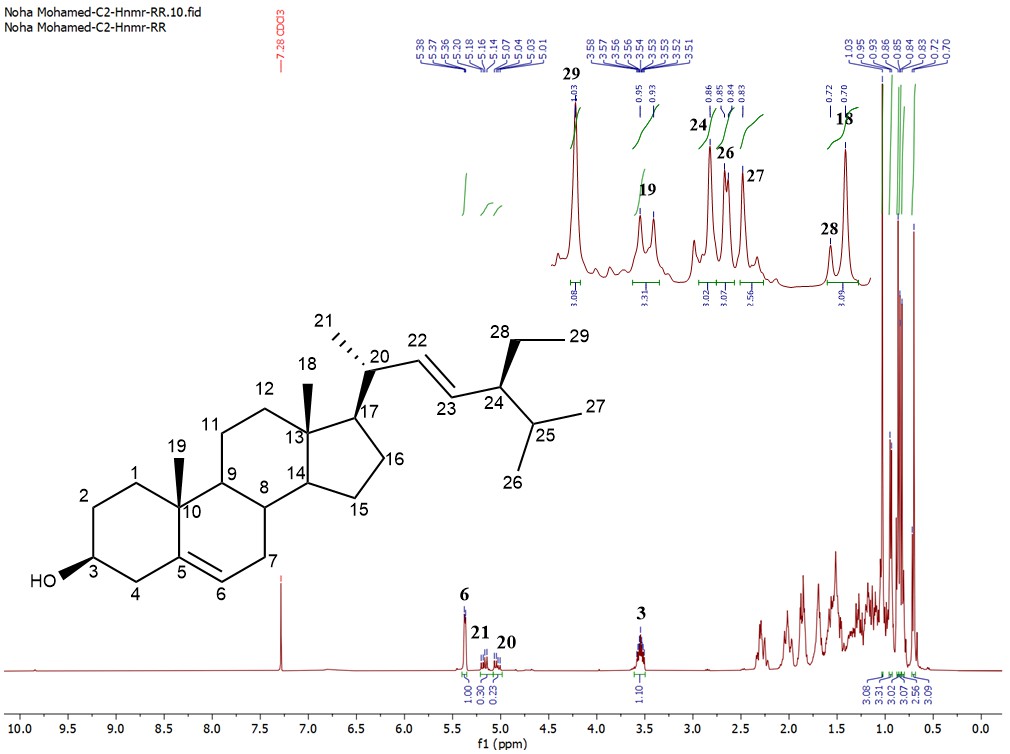


**Figure S51.** ^1^H-NMR Spectrum (CDCl_3_, 400 MHz) of compound **2** (Stigmasterol)**.**


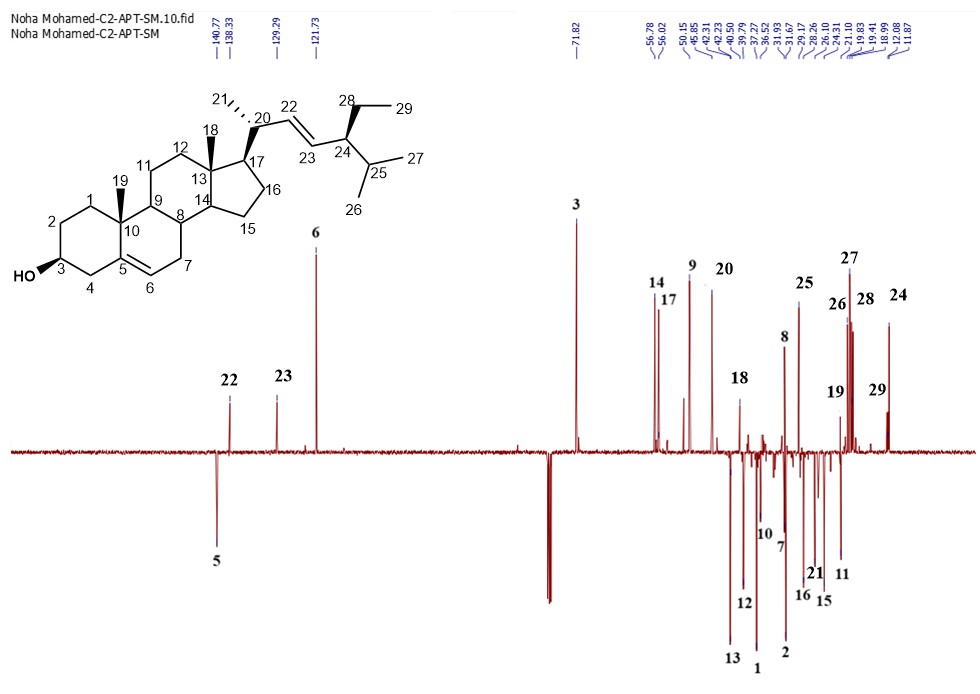


**Figure S52.** APT Spectrum (CDCl_3_, 100 MHz) of compound **2** (Stigmasterol)**.**


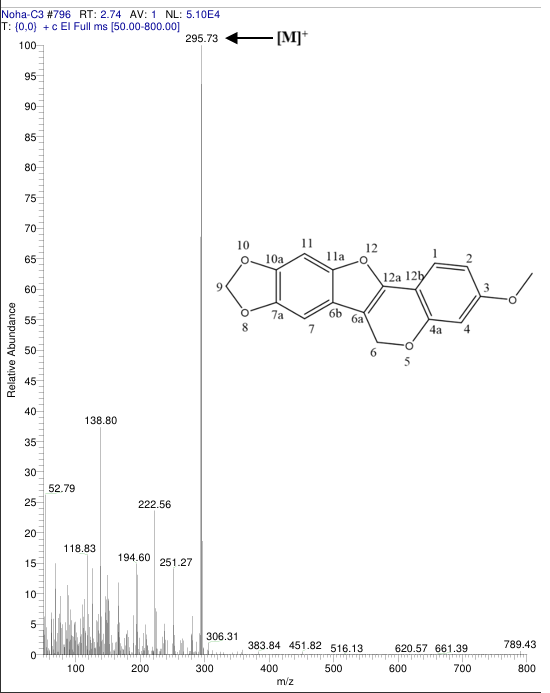


**Figure S53.** EI-MS spectra of compound **3** (Anhydropisatin), showing [M]^+^ ion peak at *m/z* 295.73.


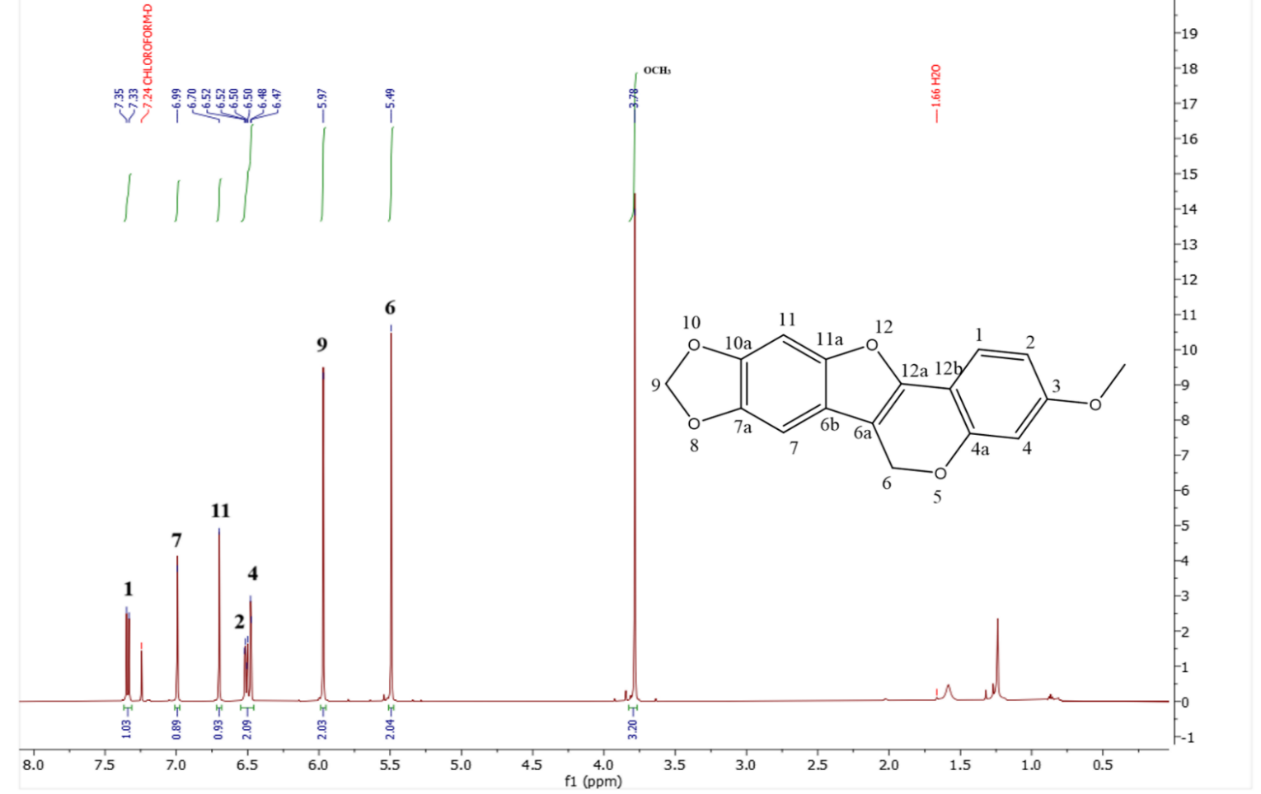


**Figure S54.** ^1^H-NMR Spectrum (CDCl_3_, 500MHz) of compound **3** (Anhydropisatin)**.**

**(a) (b)**


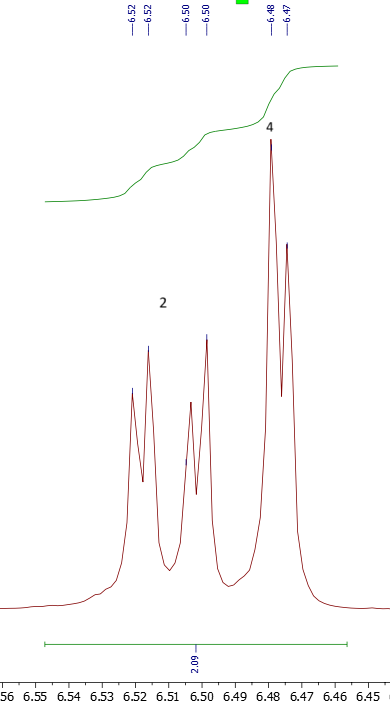

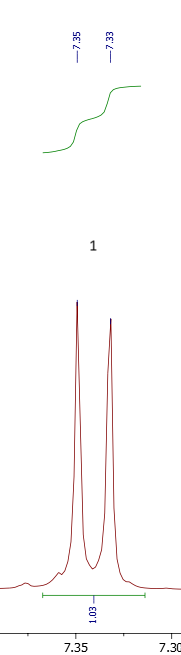


**Figure S55.** Expansions of ^1^H-NMR spectrum (500 MHz, CDCl_3_) of compound **3** (Anhydropisatin); (a) From δ 6.45−6.55 ppm and (b) from δ 7.30−7.35 ppm.


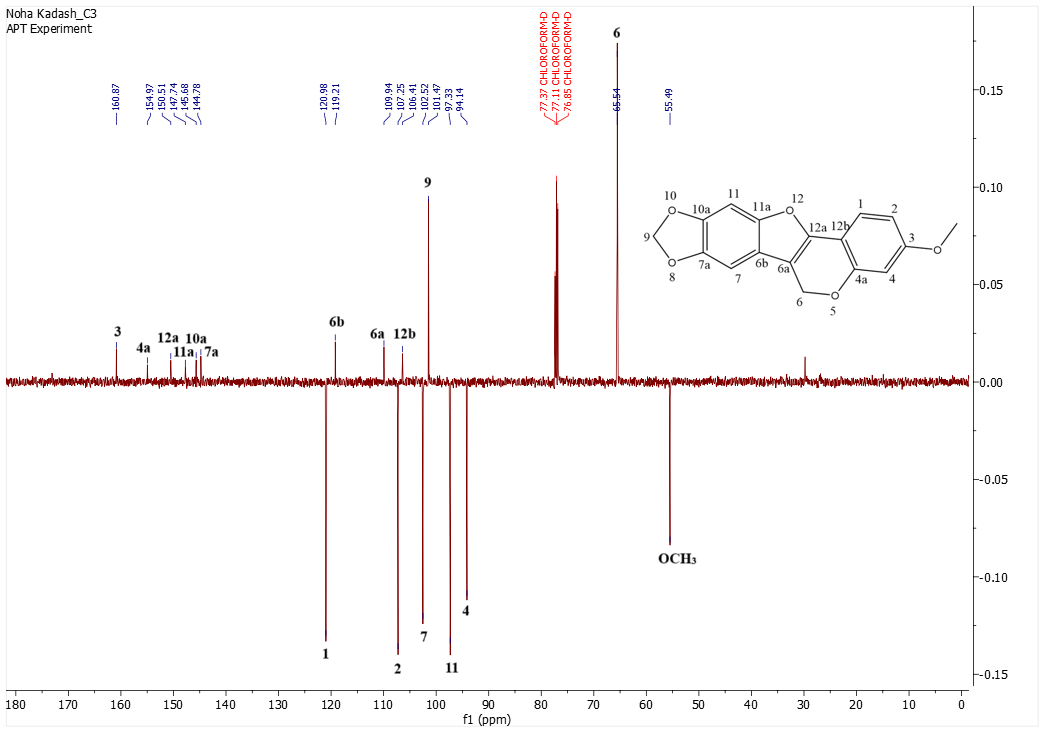
**Figure S56.** APT spectrum (CDCl_3_, 100 MHz) of compound **3** (Anhydropisatin)**.**

**Figure S57.** EI-MS spectra of compound 4 (pisatin), showing [M]^+^ ion peak at *m/z* 314.38.

**Figure S58.** ^1^H-NMR Spectrum (CDCl_3_, 400 MHz) of compound **4** (pisatin)**.**

**Figure S59.** Expansions of ^1^H-NMR spectrum (400 MHz, CDCl_3_) of compound **4** (pisatin) from δ 6.30−7.05 ppm.

**Figure S60.** APT spectrum (CDCl_3_, 100 MHz) of compound **4** (pisatin)**.**

**Figure S61.** EI-MS spectra of compound **5** (*p*-Hydroxy benzoic acid), showing [M]^+^ ion peak at *m/z* 138.00.

**Figure S62.** ^1^H-NMR spectrum (400 MHz in CD_3_OD) of compound **5** (*p*-Hydroxy benzoic acid)**.**

**Figure S63.** APT spectrum (CD_3_OD, 100 MHz) of compound **5** (*p*-Hydroxy benzoic acid)**.**

**Figure S64.** ESI-MS spectra of compound **6 (**quercetin-3-*O*-*β*-D-glucopyranoside), showing [M+Na]^+^ ion peak at *m/z* 486.2.

**Figure S65.** ESI-MS spectra of compound **6 (**quercetin-3-*O*-*β*-D-glucopyranoside), showing [M-2H]^-^ ion peak at *m/z* 462.4.

**Figure S66.** ^1^H-NMR spectrum (400 MHz in CD_3_OD) of compound **6 (**quercetin-3-*O*-*β*-D-glucopyranoside)**.**

**Figure S67.** Expansions of ^1^H-NMR spectrum (400 MHz, CD_3_OD) of compound **6 (**quercetin-3-*O*-*β*-D-glucopyranoside); from δ 5.00−7.8 ppm.

**Figure S68.** Expansions of ^1^H-NMR spectrum (400 MHz, CD_3_OD) of compound **6 (**quercetin-3-*O*-*β*-D-glucopyranoside); from δ 2.95−3.70 ppm.

**Figure S69.** APT spectrum (CD_3_OD, 100 MHz) of compound **6 (**quercetin-3-*O*-*β*-D-glucopyranoside)**.**

**Figure S70.** ESI-MS spectra of compound **7 (**quercetin-3-*O*-(6''''-*O*-*cis*-*p*-coumaroyl)-sophorotrioside), showing [M-2H]^-^ ion peak at *m/z* 931.8.

**Figure S71.** ESI-MS spectra of compound **7 (**quercetin-3-*O*-(6''''-*O*-*cis*-*p*-coumaroyl)-sophorotrioside), showing [M-2H+Na]^+^ ion peak at *m/z* 955.4.

**Figure S72.** ^1^H-NMR spectrum (500 MHz in CD_3_OD) of compound **7 (**quercetin-3-*O*-(6''''-*O*-*cis*-*p*-coumaroyl)-sophorotrioside)**.**

**Figure S73.** Expansions of ^1^H-NMR spectrum (500 MHz, CD_3_OD) of compound **7 (**quercetin-3-*O*-(6''''-*O*-*cis*-*p*-coumaroyl)-sophorotrioside); from δ 6−7.8 ppm.

**Figure S74.** Expansions of ^1^H-NMR spectrum (500 MHz, CD_3_OD) of compound **7 (**quercetin-3-*O*-(6''''-*O*-*cis*-*p*-coumaroyl)-sophorotrioside); from δ 3−6.5 ppm.

**Figure S75.** APT spectrum (CD_3_OD, 125 MHz) of compound **7 (**quercetin-3-*O*-(6''''-*O*-*cis*-*p*-coumaroyl)-sophorotrioside).

**Figure S76.** Expansions of ^13^C-NMR spectrum (125 MHz, CD_3_OD) of compound **7 (**quercetin-3-*O*-(6''''-*O*-*cis*-*p*-coumaroyl)-sophorotrioside); from δ 60−78 ppm.

**2.5.** **Evaluation of the immunomodulatory activity of** **pure compounds 3, 4, 6, and 7 isolated from PSP and PSE fractions of *P. sativum* L.**

*2.5.1. Impact on cell viability* *of human monocyte cells (CRL-9855™)*

**Figure S77.** Cell metabolic activity (MTS assay) of human monocyte cells (CRL-9855™) treated with LPS alone or in combination with dexamethasone (Dexa) or different concentrations (1, 10, 20 μM) of compounds **3**, **4**, **6**, **7**. Data are presented as mean ± SEM relative to control (100%).

*2.5.2. Impact of compounds 3, 4, 6, and 7 on Reactive Oxygen Species (ROS) generation in human monocytes*

**Figure S78.** Flow cytometry assay of intracellular ROS levels using CM-H2DCFDA as the ROS probe in monocytes following LPS (50 ng/mL) pre-treatment and subsequent exposure to the tested compounds (5 and 10 µM) for 24 hours.

*2.5.3.* *Impact* *of compounds 3, 4, 6, and 7 on cell viability in* *HaCaT Keratinocytes*

**Figure S79.** Cell metabolic activity of HaCaT Keratinocytes treated with different concentrations (0.5, 1, 5, 10, 20, 40, 80 μM) of compounds **3**, **4**, **6**, and **7**. Cell viability was assessed using crystal violet assay. Data is presented as mean ± SEM relative to control (100%).

**2.6.** ***In-silico* prediction of pisatin molecular targets and molecular docking analysis**

*2.6.1.* *Molecular target prediction and Network pharmacology*

**Figure S80**. Enrichment analysis of terms related to wound healing and targets associated with pisatin activity. (A) KEGG, (B) biological processes, (C) molecular functions, and (D) cellular components.

**Figure S81.** (a) Protein–protein interaction network constructed from the most relevant targets associated with the activity of pisatin. (b) The top 5 genes included in KEGG pathways (Sorted by the number of KEGG pathways related to gene. The greater the degree of overlap of genes, the more the same pathways involved. Lines with different colors indicate different gene-pathway relationships.

*2.6.2. Molecular Docking analysis*

**Figure S82.** Molecular interactions of pisatin with the PI3K complex (PDB: 3HHM). Structural model of Pisatin (CPK green) interacting with the PI3K heterodimer: catalytic subunit p110α (Chain A, red) and regulatory subunit p85α (blue).

**Table S5.** Blind Docking Results of pisatin in Different Binding Sites in the PI3K Complex

| **ID** | | **Cavities volume**  **(Å^3^)** | **Docking Site coordinate (X, Y, Z)** | | | **Box size**  **(X, Y, Z)** | | | **Vina score** |
| --- | --- | --- | --- | --- | --- | --- | --- | --- | --- |
| **Site-1** | | 7352 | 65.531 | 78.837 | 91.418 | 35 | 29 | 35 | -4.9 |
| **Site-2** | **P1** | 32498 | 72.044 | 51.162 | 80.793 | 35 | 35 | 35 | -8.9 |
|  | **P2** | 3805 | 89.520 | 79.007 | 92.443 | 21 | 30 | 21 | -7.9 |

**References**

1. Resende, M., et al., *Novel phytoalexins including elemental sulphur in the resistance of cocoa (Theobroma cocoaL.) to Verticillium wilt (Verticillium dahliaeKleb.).* Physiological and molecular plant pathology, 1996. **48**(5): p. 347-359.

2. El-Said, M. and M. Amer, *Oils, fats, waxes and surfactants.* Anglo Egyptian Book Shop, Cairo, 1965: p. 130-132.

3. Ebrahimzadeh, M.A., F. Pourmorad, and A.R. Bekhradnia, *Iron chelating activity, phenol and flavonoid content of some medicinal plants from Iran.* African journal of Biotechnology, 2008. **7**(18).

4. Biglari, F., A.F. AlKarkhi, and A.M. Easa, *Antioxidant activity and phenolic content of various date palm (Phoenix dactylifera) fruits from Iran.* Food chemistry, 2008. **107**(4): p. 1636-1641.

5. Sawicka-Gutaj, N., et al., *Publication ethics of human studies in the light of the Declaration of Helsinki–a mini-review.* Journal of Medical Science, 2022. **91**(2): p. e700-e700.

6. Luttmann, W., et al., *Der experimentator: immunologie*. 2014: Springer-Verlag.

7. Seeligmüller, N., *Faster isolation of PBMC using Ficoll-Paque. Plus in the Eppendorf® centrifuge 5920 R.* Germany, Hamburg: Eppendorf AG, 2016.

8. Ishiyama, M., et al., *A new sulfonated tetrazolium salt that produces a highly water-soluble formazan dye.* Chemical and Pharmaceutical Bulletin, 1993. **41**(6): p. 1118-1122.

9. Miranda, K.M., M.G. Espey, and D.A. Wink, *A rapid, simple spectrophotometric method for simultaneous detection of nitrate and nitrite.* Nitric oxide, 2001. **5**(1): p. 62-71.

10. Sameh, M., et al., *Integrated multiomics analysis to infer COVID-19 biological insights.* Scientific Reports, 2023. **13**(1): p. 1802.

11. Hegazy, M.M., et al., *Biological and chemical evaluation of some African plants belonging to Kalanchoe species: Antitrypanosomal, cytotoxic, antitopoisomerase I activities and chemical profiling using ultra-performance liquid chromatography/quadrupole-time-of-flight mass spect.* Pharmacognosy Magazine, 2021. **17**(73).

12. El Hamaky, N.F., et al., *Novel quinazolin-2-yl 1, 2, 3-triazole hybrids as promising multi-target anticancer agents: Design, synthesis, and molecular docking study.* 2024. **148**: p. 107437.

13. Izzo, F.C., *20th century artists' oil paints: a chemical-physical survey.* 2011.

14. Villalobos Solis, M.I., et al., *Fatty acid profiling of the seed oils of some varieties of field peas (Pisum sativum) by RP-LC/ESI-MS/MS: towards the development of an oilseed pea.* Food Chem, 2013. **139**(1-4): p. 986-93.

15. Mazurek, B., M. Chmiel, and B. Górecka, *Fatty acids analysis using gas chromatography-mass spectrometer detector (GC/MSD)-method validation based on berry seed extract samples.* Food Analytical Methods, 2017. **10**: p. 2868-2880.

16. Hejazi, L., et al., *Discrimination among geometrical isomers of α-linolenic acid methyl ester using low energy electron ionization mass spectrometry.* Journal of the American Society for Mass Spectrometry, 2009. **20**: p. 1272-1280.

17. Zhou, L., J. Li, and C. Yan, *Simultaneous determination of three flavonoids and one coumarin by LC–MS/MS: Application to a comparative pharmacokinetic study in normal and arthritic rats after oral administration of Daphne genkwa extract.* Biomedical Chromatography, 2018. **32**(7): p. e4233.

18. Šibul, F., et al., *Phenolic profile, antioxidant and anti-inflammatory potential of herb and root extracts of seven selected legumes.* Industrial Crops and Products, 2016. **83**: p. 641-653.

19. Du, L.-y., et al., *Analysis of the metabolites of isorhamnetin 3-O-glucoside produced by human intestinal flora in vitro by applying ultraperformance liquid chromatography/quadrupole time-of-flight mass spectrometry.* Journal of agricultural and food chemistry, 2014. **62**(12): p. 2489-2495.

20. Jiang, C. and P.J. Gates, *Systematic Characterisation of the Fragmentation of Flavonoids Using High-Resolution Accurate Mass Electrospray Tandem Mass Spectrometry.* Molecules, 2024. **29**(22): p. 5246.

21. Mejri, F., et al., *In vitro and in vivo biological properties of pea pods (Pisum sativum L.).* Food Bioscience, 2019. **32**: p. 100482.

22. Attallah, N.G., et al., *Elucidation of phytochemical content of Cupressus macrocarpa leaves: in vitro and in vivo antibacterial effect against methicillin-resistant Staphylococcus aureus clinical isolates.* Antibiotics, 2021. **10**(8): p. 890.

23. Abdl Aziz, F.T., A.S. Temraz, and M.A. Hassan, *Metabolites profiling by LC-ESI-MS/MS technique and in-vitro antioxidant activity of Bauhinia madagascariensis Desv. and Bauhinia purpurea L. aerial parts cultivated in Egypt: A comparative study.* Azhar International Journal of Pharmaceutical and Medical Sciences, 2024. **4**(1): p. 169-188.

24. Ma, Y.-L., et al., *Internal glucose residue loss in protonated O-diglycosyl flavonoids upon low-energy collision-induced dissociation.* Journal of the American Society for Mass Spectrometry, 2000. **11**(2): p. 136-144.

25. Zilani, M.N.H., et al., *Chemical composition and pharmacological activities of Pisum sativum.* BMC complementary and alternative medicine, 2017. **17**: p. 1-9.

26. Neugart, S., S. Rohn, and M. Schreiner, *Identification of complex, naturally occurring flavonoid glycosides in Vicia faba and Pisum sativum leaves by HPLC-DAD-ESI-MSn and the genotypic effect on their flavonoid profile.* Food Research International, 2015. **76**: p. 114-121.

27. Koolen, H.H., et al., *Antioxidant, antimicrobial activities and characterization of phenolic compounds from buriti (Mauritia flexuosa L. f.) by UPLC–ESI-MS/MS.* Food Research International, 2013. **51**(2): p. 467-473.

28. Regos, I., A. Urbanella, and D. Treutter, *Identification and quantification of phenolic compounds from the forage legume sainfoin (Onobrychis viciifolia).* Journal of Agricultural and Food Chemistry, 2009. **57**(13): p. 5843-5852.

29. Seida, A.A., et al., *Bioassay-guided fractionation of a hepatoprotective and antioxidant extract of pea by-product.* Natural product research, 2015. **29**(16): p. 1578-1583.

30. Ferreres, F., et al., *Acylated flavonol sophorotriosides from pea shoots.* Phytochemistry, 1995. **39**(6): p. 1443-1446.

31. Ouyang, H., et al., *Identification and quantification analysis on the chemical constituents from traditional mongolian medicine flos scabiosae using uhplc–dad–q-tof-ms combined with uhplc–qqq-ms.* Journal of chromatographic science, 2016. **54**(6): p. 1028-1036.

32. Zhou, C., et al., *A sensitive LC–MS–MS method for simultaneous quantification of two structural isomers, hyperoside and isoquercitrin: application to pharmacokinetic studies.* Chromatographia, 2011. **73**: p. 353-359.

33. Ding, X., et al., *Metabolomics studies on cytoplasmic male sterility during flower bud development in soybean.* International Journal of Molecular Sciences, 2019. **20**(12): p. 2869.

34. Murakami, T., et al., *Medicinal foodstuffs. XXV. Hepatoprotective principle and structures of ionone glucoside, phenethyl glycoside, and flavonol oligoglycosides from young seedpods of garden peas, Pisum sativum L.* Chemical and pharmaceutical bulletin, 2001. **49**(8): p. 1003-1008.

35. Jiang, Z., et al., *Simultaneous determination of kaempferide, kaempferol and isorhamnetin in rat plasma by ultra-high performance liquid chromatography-tandem mass spectrometry and its application to a pharmacokinetic study.* Journal of the Brazilian chemical society, 2018. **29**(3): p. 535-542.

36. Parejo, I., et al., *Separation and characterization of phenolic compounds in fennel (Foeniculum vulgare) using liquid chromatography− negative electrospray ionization tandem mass spectrometry.* Journal of agricultural and food chemistry, 2004. **52**(12): p. 3679-3687.

37. Mohammed, M.M., et al., *Chemical Composition and Liver Protection of the Methanol Extract of Fortunella margarita (Lour.) Swingle Leaves.*

38. Stanisavljević, N.S., et al., *Identification of seed coat phenolic compounds from differently colored pea varieties and characterization of their antioxidant activity.* Archives of biological sciences, 2015. **67**(3): p. 829-840.

39. Tadić, V., et al., *The estimation of the traditionally used yarrow (Achillea millefolium L. Asteraceae) oil extracts with anti-inflamatory potential in topical application.* Journal of ethnopharmacology, 2017. **199**: p. 138-148.

40. Chahbani, A., et al., *Microwave drying effects on drying kinetics, bioactive compounds and antioxidant activity of green peas (Pisum sativum L.).* Food Bioscience, 2018. **25**: p. 32-38.

41. Riyazuddin, M., et al., *Simultaneous quantification of five biomarkers in ethanolic extract of Cassia occidentalis Linn. stem using liquid chromatography tandem mass spectrometry: application to its pharmacokinetic studies.* RSC advances, 2020. **10**(8): p. 4579-4588.

42. Benayad, Z., C. Gómez-Cordovés, and N.E. Es-Safi, *Identification and quantification of flavonoid glycosides from fenugreek (Trigonella foenum-graecum) germinated seeds by LC–DAD–ESI/MS analysis.* Journal of Food Composition and Analysis, 2014. **35**(1): p. 21-29.

43. Dueñas, M., I. Estrella, and T. Hernández, *Occurrence of phenolic compounds in the seed coat and the cotyledon of peas (Pisum sativum L.).* European Food Research and Technology, 2004. **219**: p. 116-123.

44. Krasteva, I. and S. Nikolov, *Flavonoids in Astragalus corniculatus.* Química Nova, 2008. **31**: p. 59-60.

45. Álvarez-Fernández, M.A., et al., *Composition of nonanthocyanin polyphenols in alcoholic-fermented strawberry products using LC–MS (QTRAP), high-resolution MS (UHPLC-Orbitrap-MS), LC-DAD, and antioxidant activity.* Journal of agricultural and food chemistry, 2015. **63**(7): p. 2041-2051.

46. Attallah, N.G., et al., *Elucidation of the metabolite profile of Yucca gigantea and assessment of its cytotoxic, antimicrobial, and anti-inflammatory activities.* Molecules, 2022. **27**(4): p. 1329.

47. Han, X., et al., *Naringenin-7-O-glucoside protects against doxorubicin-induced toxicity in H9c2 cardiomyocytes by induction of endogenous antioxidant enzymes.* Food and Chemical Toxicology, 2008. **46**(9): p. 3140-3146.

48. Zheng, Y., et al., *Integrating pharmacology and gut microbiota analysis to explore the mechanism of Citri reticulatae pericarpium against reserpine-induced spleen deficiency in rats.* Frontiers in Pharmacology, 2020. **11**: p. 586350.

49. Abu‐Reidah, I.M., et al., *UHPLC‐ESI‐QTOF‐MS‐based metabolic profiling of Vicia faba L.(Fabaceae) seeds as a key strategy for characterization in foodomics.* Electrophoresis, 2014. **35**(11): p. 1571-1581.

50. Lapčı́k, O., et al., *Immunoanalysis of isoflavonoids in Pisum sativum and Vigna radiata.* Plant Science, 1999. **148**(2): p. 111-119.

51. Piersen, C., et al., *Chemical and biological characterization and clinical evaluation of botanical dietary supplements: a phase I red clover extract as a model.* Current medicinal chemistry, 2004. **11**(11): p. 1361-1374.

52. El-Shial, E.M., et al., *Elucidation of natural components of Gardenia Thunbergia Thunb. Leaves: effect of methanol extract and Rutin on non-alcoholic fatty liver disease.* Molecules, 2023. **28**(2): p. 879.

53. Veitch, N.C., *Isoflavonoids of the Leguminosae.* Natural Product Reports, 2009. **26**(6): p. 776-802.

54. Taha, K., et al., *The pericarp of Pisum sativum L.(Fabaceae) as a biologically active waste product.* Planta Medica, 2011. **77**(12): p. PJ22.

55. Fathoni, A., A. Candraditya, and T. Rudiana, *Antioxidant activity and identification of flavonoid compounds in Patat.* J. Pendidik. Kim, 2022. **14**: p. 149-156.

56. Han, X., et al., *Comprehensive compositional assessment of bioactive compounds in diverse pea accessions.* Food Research International, 2023. **165**: p. 112455.

57. Statham, C.M., R. Crowden, and J. Harborne, *Biochemical genetics of pigmentation in Pisum sativum.* Phytochemistry, 1972. **11**(3): p. 1083-1088.

58. Gong, E.S., et al., *Identification of key phenolic compounds responsible for antioxidant activities of free and bound fractions of blackberry varieties' extracts by boosted regression trees.* Journal of the Science of Food and Agriculture, 2022. **102**(3): p. 984-994.

59. Barnes, J.S. and K.A. Schug, *Structural characterization of cyanidin-3, 5-diglucoside and pelargonidin-3, 5-diglucoside anthocyanins: Multi-dimensional fragmentation pathways using high performance liquid chromatography-electrospray ionization-ion trap-time of flight mass spectrometry.* International Journal of Mass Spectrometry, 2011. **308**(1): p. 71-80.

60. Lin, L.-Z., et al., *The polyphenolic profiles of common bean (Phaseolus vulgaris L.).* Food chemistry, 2008. **107**(1): p. 399-410.

61. Yang, L.-C. and S.-F. Chen, *Quantification of anthocyanosides in grapes by QuEChERS and biphenyl-UHPLC tandem mass spectrometry.* Journal of Food and Drug Analysis, 2022. **30**(3): p. 382.

62. Fahim, J.R., E.Z. Attia, and M.S. Kamel, *The phenolic profile of pea (Pisum sativum): a phytochemical and pharmacological overview.* Phytochemistry Reviews, 2019. **18**: p. 173-198.

63. Zhong, X., et al., *Comparative analysis of transcriptome and metabolome explores the underlying mechanism of pod color variation in pea (Pisum sativum L.).* Journal of Plant Biochemistry and Biotechnology, 2024: p. 1-13.

64. Guo, F., et al., *Phenolics of green pea (Pisum sativum L.) hulls, their plasma and urinary metabolites, bioavailability, and in vivo antioxidant activities in a rat model.* Journal of Agricultural and Food Chemistry, 2019. **67**(43): p. 11955-11968.

65. Kondo, K., et al., *Mechanistic studies of catechins as antioxidants against radical oxidation.* Archives of Biochemistry and Biophysics, 1999. **362**(1): p. 79-86.

66. Stöggl, W., C. Huck, and G.K. Bonn, *Structural elucidation of catechin and epicatechin in sorrel leaf extracts using liquid‐chromatography coupled to diode array‐, fluorescence‐, and mass spectrometric detection.* Journal of separation science, 2004. **27**(7‐8): p. 524-528.

67. Xu, Y., et al., *The impact of citrus-tea cofermentation process on chemical composition and contents of Pu-erh tea: An integrated metabolomics study.* Frontiers in nutrition, 2021. **8**: p. 737539.

68. Kerwin, J.L., *Negative ion electrospray mass spectrometry of polyphenols, catecholamines and their oxidation products.* Journal of mass spectrometry, 1996. **31**(12): p. 1429-1439.

69. Stanisavljević, N.S., et al., *Identification of phenolic compounds from seed coats of differently colored European varieties of pea (Pisum sativum L.) and characterization of their antioxidant and in vitro anticancer activities.* Nutrition and cancer, 2016. **68**(6): p. 988-1000.

70. Pearson, J.L., et al., *The liquid chromatographic determination of chlorogenic and caffeic acids in Xu Duan (Dipsacus asperoides) raw herb.* International Scholarly Research Notices, 2014. **2014**(1): p. 968314.

71. Hossain, M.B., et al., *Characterization of phenolic composition in Lamiaceae spices by LC-ESI-MS/MS.* Journal of agricultural and food chemistry, 2010. **58**(19): p. 10576-10581.

72. Sosulski, F.W. and K.J. Dabrowski, *Composition of free and hydrolyzable phenolic acids in the flours and hulls of ten legume species.* Journal of Agricultural and Food Chemistry, 1984. **32**(1): p. 131-133.

73. Karonen, M. and J.-M. Pihlava, *Identification of Oxindoleacetic acid conjugates in quinoa (Chenopodium quinoa Willd.) seeds by high-resolution UHPLC-MS/MS.* Molecules, 2022. **27**(17): p. 5629.

74. Fang, N., S. Yu, and R.L. Prior, *LC/MS/MS characterization of phenolic constituents in dried plums.* Journal of Agricultural and Food Chemistry, 2002. **50**(12): p. 3579-3585.

75. El-sayed, M., et al., *UPLC-ESI-MS/MS profile of the ethyl acetate fraction of aerial parts of Bougainvillea'Scarlett O'Hara'cultivated in Egypt.* Egyptian Journal of Chemistry, 2021. **64**(2): p. 793-806.

76. Singh, B., et al., *Phenolic composition and antioxidant potential of grain legume seeds: A review.* Food research international, 2017. **101**: p. 1-16.

77. Troszyńska, A. and B. Bałasińska, *Antioxidant activity of crude tannins of pea (Pisum sativum L.) seed coat and their hypocholesterolemic effect in rats.* Polish journal of food and nutrition sciences, 2002. **52**(3): p. 33-38.

78. Weesepoel, Y., et al., *Protocatechuic acid levels discriminate between organic and conventional wheat from Denmark.* Chimia, 2016. **70**(5): p. 360-360.

79. CT, S., et al., *Chemical profiling of a polyherbal formulation by tandem mass spectroscopic analysis with multiple ionization techniques.* Future Journal of Pharmaceutical Sciences, 2020. **6**: p. 1-8.

80. Mukhtarova, L.S., et al., *Hydroperoxide lyase cascade in pea seedlings: Non-volatile oxylipins and their age and stress dependent alterations.* Phytochemistry, 2011. **72**(4-5): p. 356-364.

81. Zhao, Q., et al., *Chemometrics strategy coupled with high resolution mass spectrometry for analyzing and interpreting comprehensive metabolomic characterization of hyperlipemia.* RSC Advances, 2016. **6**(113): p. 112534-112543.

82. Tadera, K., et al., *Isolation and structure of a new metabolite of pyridoxine in seedlings of Pisum sativum L.* Agricultural and Biological Chemistry, 1983. **47**(6): p. 1357-1359.

83. Hinners, P., K.C. O’Neill, and Y.J. Lee, *Revealing individual lifestyles through mass spectrometry imaging of chemical compounds in fingerprints.* Scientific reports, 2018. **8**(1): p. 5149.

84. Ji, S.-H., et al., *Isolation and Identification of Secondary Metabolites from the Ovary of Nelumbo nucifera.* Journal of Life Science, 2016. **26**(10): p. 1196-1201.

85. Basak, G.K., et al., *Phytochemical and Antimicrobial Activity of the Leaves of Croton Bonplandianum Bail.* Journal of Advanced Scientific Research, 2021. **12**(01): p. 53-56.

86. Negm, W.A., et al., *Hepatoprotective, cytotoxic, antimicrobial and antioxidant activities of Dioon spinulosum leaves Dyer Ex Eichler and its isolated secondary metabolites.* Natural Product Research, 2021. **35**(23): p. 5166-5176.

87. Ozaki, Y., K. Mochida, and S.-W. Kim, *Total synthesis of sophorapterocarpan A, maackiain, and anhydropisatin: application of a 1, 3-Michael-Claisen annulation to aromatic synthesis.* Journal of the Chemical Society, Perkin Transactions 1, 1989(7): p. 1219-1224.

88. Kato-Noguchi, H., *Isolation and identification of an allelopathic substance in Pisum sativum.* Phytochemistry, 2003. **62**(7): p. 1141-1144.

89. Wang, M., et al., *A 4-hydroxybenzoic acid-mediated signaling system controls the physiology and virulence of Shigella sonnei.* Microbiology Spectrum, 2023. **11**(3): p. e04835-22.

90. Amado, N.G., et al., *Isoquercitrin isolated from Hyptis fasciculata reduces glioblastoma cell proliferation and changes β-catenin cellular localization.* Anti-cancer drugs, 2009. **20**(7): p. 543-552.
